# Radical Ring Opening Polymerization of Cyclic Ketene Acetals Derived From d-Glucal

**DOI:** 10.1021/acsmacrolett.3c00397

**Published:** 2023-10-12

**Authors:** Craig Hardy, Martin E. Levere, Gabriele Kociok-Köhn, Antoine Buchard

**Affiliations:** † Department of Chemistry, University of Bath, Claverton Down, Bath, BA2 7AY, United Kingdom; ‡ Materials and Chemical Characterisation Facility (MC^2^), University of Bath, Claverton Down, Bath, BA2 7AY, United Kingdom; § University of Bath Institute for Sustainability, Claverton Down, Bath, BA2 7AY, United Kingdom

## Abstract

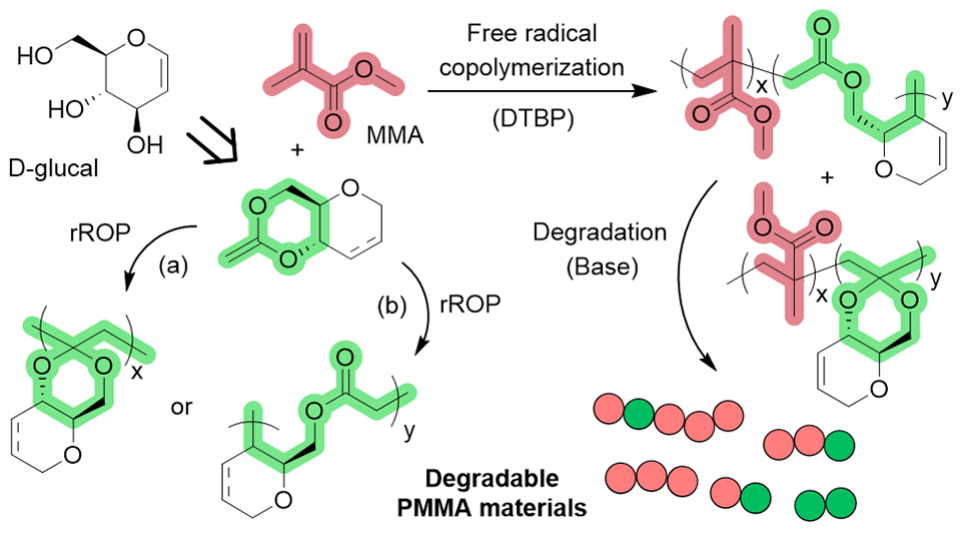

A cyclic ketene acetal (CKA) derived from d-glucal was synthesized, and its polymerization using free radicals has been investigated. NMR analysis of the resulting polymers revealed the formation of polyacetal–polyester copolymers, with up to 78% of ester linkages formed by radical ring-opening polymerization (rROP). Conversely, the polymerization of the monomer-saturated analogue only produced acetal linkages, demonstrating that the alkene functionality within the d-glucal pyranose ring is essential to promote ring-opening and ester formation, likely via the stabilization of an allyl radical. The thermal properties of the polymers were linked to the ratio of the ester and acetal linkages. Copolymerization with methyl methacrylate (MMA) afforded statistically PMMA-rich copolymers (66–98%) with linkages prone to hydrolytic degradation and decreased glass-transition temperatures. The retention of the pseudoglucal alkene function offers opportunities to functionalize further these bioderived (co)polymers.

Amid our reliance on fossil resources, the persistence of most synthetic polymers and the challenges still associated with their waste management have proven costly to the environment.^1−4^ As a result, polymers derived from renewable resources are of significant interest, including because they usually feature oxygenated linkages that often favor degradability and chemical recycling.^4−6^ Ionic, organocatalytic, or coordination–insertion ring-opening polymerization (ROP)^7^ are among several well-established methods to generate such biobased polymers, e.g., poly(lactic acid) (PLA).^8,9^ However, for such an ROP, monomers must contain polarized functional groups in order for heterolytic cleavage to occur. Radical ring-opening polymerization (rROP) does not possess the same limitations and has recently been explored as a potential alternative.^10−13^ Among the monomers amenable to rROP, cyclic ketene acetals (CKA) are electron-rich vinyl monomers that offer a route toward aliphatic polyesters akin to established polymers such as PLA, polyglycolide, and polycaprolactone.^11,14^ The rROP of CKAs toward polyesters have been exploited for the synthesis of homopolymers,^15−19^ block copolymers,^20−23^ and statistical copolymers with vinylic monomers, allowing in the latter the introduction of degradable units within the polymer backbone.^24−27^ CKA copolymers have also been applied in the polymerization-induced self-assembly of degradable nanoparticles.^28^

Compared to other monomers used in rROP (e.g., vinyl cycloalkanes, cyclic vinyl ethers),^11^ the relative ease of CKA synthesis from widely available diols (including bioderived 1,4-butanediol, and diethylene glycol) is advantageous.^29^ Yet, the most investigated monomers remain by far 2-methylene-1,3-dioxepane (MDO), 5,6-benzo-2-methylene-1,3-dioxepane (BMDO), and 2-methylene-4-phenyl-1,3-dioxolane (MPDL). Indeed, one challenge in the rROP of CKAs is the probability of the 1,2-vinyl addition over ring opening, which does not lead to ester linkages.^11^

We, among others, have been exploring sugar-derived polymers as a renewable, structurally diverse, and functionalizable alternative to polymers produced from fossil-based resources.^30−44^ In particular, the use of tri-*O*-acetyl-d-glucal, a commercial derivative of d-glucose, as synthetic precursor for novel monomers, was pioneered by Wooley and co-workers^45^ and has allowed the incorporation of an alkene group into sugar-derived polymer structures.^36,46^ In the context of CKA rROP, our hypothesis was that this internal alkene functionality would favor ring fragmentation by stabilizing radicals and promoting the formation of ester linkages. Herein, we report the synthesis and rROP of a novel CKA monomer derived from d-glucal and its copolymerization with methyl methacrylate (MMA), yielding vinyl copolymers prone to hydrolytic degradation.

Expanding upon previously developed methods for the synthesis of CKAs, as well as previous work within our group,^25,36^ CKA **1** was synthesized in four high-yielding synthetic steps from tri-*O*-acetyl-d-glucal, via Ferrier rearrangement, deprotection under Zemplén conditions, acid-catalyzed transacetalization, and cyclization by dehydrobromination (Scheme 1; 52% overall yield).

**Scheme 1:**
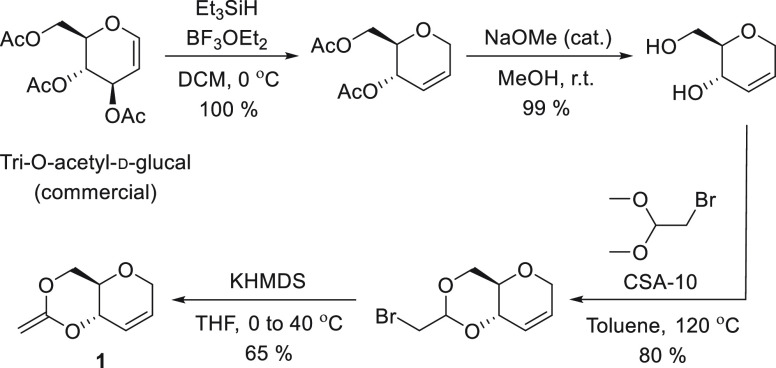
Synthesis of CKA Monomer **1**

When using triethylsilane in the Ferrier rearrangement, the resulting pseudoglucal exists only as a single anomer, allowing for simpler purification and characterization. In addition, when methanol and propan-2-ol were used as nucleophiles, later transacetalization reactions consistently failed. Transacetalization with dimethyl bromoacetal was performed at 120 °C, using (1*S*)-(+)-10-camphorsulfonic acid (CSA-10) as catalyst. The molecular structure and stereochemistry of the bicyclic bromoacetal was further corroborated by single crystal X-ray crystallography (Figure S13). Potassium bis(trimethylsilyl)amide (KHMDS) was used for the dehydrobromination step, as a strong and non-nucleophilic base, to circumvent the formation of the nucleophilic substitution side product (a major impurity when using *t*-BuOK). After precipitation in anhydrous pentane at −78 °C, cyclic ketene acetal **1**, was isolated as a colorless oil and characterized by multinuclear NMR and FT-IR spectroscopies. Like previously published CKAs, **1** displays low stability toward air and moisture, due to a proficiency in undergoing acid catalyzed polymerization.^11,47^ However, it is possible to store **1** for several days under an argon atmosphere in anhydrous solution.

To test our hypothesis, the saturated counterpart, CKA **2**, was synthesized following an adapted procedure, including the hydrogenation of the 4,6-diol using Pd/C under H_2_ (Scheme S1, 59% overall yield). Indeed, as shown below (Table 1), upon addition of a radical (initiator or propagating chain) to the C–C double bond of the CKA, the resultant radical could either propagate instantly to form an acetal linkage or cascade further C–O cleavage by ring fragmentation toward forming a secondary radical, stabilized by resonance for **1** (**1a** ↔ **1b**), but not for **2**. Therefore, **2** is expected to predominantly undergo 1,2-addition, resulting in cyclic acetal linkages, while **1** should encourage ring fragmentation and ester linkages.

**Table 1:**
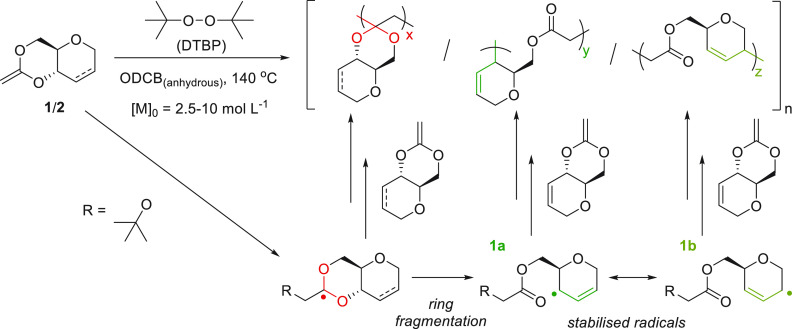
Radical Ring-Opening Polymerization of **1** and **2** with DTBP^a^

entry	[**M**]	[**M**]_0_:[I]_0_^b^	[**M**]_0_ (mol L^–1^)	conv.^c^ (%)	*F*_E_/*F*_A_^e^	*M*_n,SEC_^f^	*Đ*_M_^f^	*T*_g_^g^ (°C)	*T*_d5%_ (°C)
1^h^	**1**	50:1	8.8	>99	23/77	3.1	2.17	78	206
2	**1**	100:1	1.9	>99	78/22	1.2	1.44	65	173
3	**1**	100:1	3.2	>99	65/35	4.7	2.11	68	210
4^i^	**1**	100:1	3.2	>99	78/22	2.4	1.92	65	205
5	**1**	100:1	4.7	>99	56/44	4.3	2.53	73	204
6^i^	**1**	100:1	4.7	>99	43/57	5.1	2.37	72	230
7	**2**	100:1	5	>99^d^	0/100	1.0	1.66	83	215
8	**2**	100:1	10	>99^d^	0/100	1.4	1.83	93	214

a Reactions were carried out over 20 h at 140 °C, under an Ar atmosphere, in anhydrous ODCB with [**M**]_0_ = 1.9–10 mol L^–1^ (**M** = **1** or **2**), unless stated otherwise.

b I = DTBP.

c Monomer conversion to polymer, calculated by ^1^H NMR spectroscopy by relative integration of the methylene signal of **1** (δ_H_ = 3.85 ppm, d, 2H) and the alkyl proton signal(s) of poly(**1**) (δ_H_ = 2.85–2.28 and/or 2.17–2.02, m, 2H).

d Monomer conversion to polymer, calculated by ^1^H NMR spectroscopy by relative integration of the methylene signal of **2** (δ_H_ = 3.85 ppm, s, 2H) and the alkyl proton signal of poly(**2**) (δ_H_ = 2.72–2.06, m, 2H).

e Polymer composition between ester (*F*_E_) and acetal (*F*_A_) linkages, determined using ^13^C{^1^H} NMR spectroscopy, based on the relative integration of signals corresponding to ester (δ_C_ = 170–172 ppm) and acetal linkages (δ_C_ = 113 ppm).

f Number-average molar mass and dispersity (*M*_n,SEC_, *Đ*_M_), calculated by SEC relative to polystyrene standards in a THF eluent, units in kg mol^–1^.

g Values obtained from DSC second heating cycle.

h Polymerization was carried out in solvent-free over 20 h at 120 °C, under an argon atmosphere.

i Repeat reactions, showing deviation in *F*_E_/*F*_A_.

The free radical polymerization of **1** was first conducted without solvent at various temperatures (70–120 °C), using di-*tert*-butyl peroxide (DTBP) or 2,2′-azobis(2-methyl-propionitrile) (AIBN) as initiators (Table S1). The reactions were successful in converting **1** into oligomeric and polymeric species, but only low molar mass species could be isolated. A rapid increase in the viscosity of the reaction medium was observed, preventing stirring, and likely leading to premature chain termination events.

Therefore, polymerizations were next carried out in solution to mitigate the increase in viscosity. In addition, previous studies have found that dilution can favor the ring fragmentation pathway of CKAs.^19,48^ As a reduction in initial monomer concentration ([**M**]_0_) may decrease the overall reactivity of the monomer, all solution-based polymerizations were carried out at 140 °C, using DTBP as radical initiator due to its superior half-life at this temperature compared to AIBN. Lower temperatures have also been shown to lead to decreased ring-fragmentation.^11,49^ Initial tests were performed in high boiling point solvents, *p*-xylene and 1,2-dichlorobenzene (ODCB), with ODCB proving to be more suitable to achieve higher molar mass polymers (Table S2). As reported in Table 1, free radical polymerization of **1** in the ODCB at 140 °C, at initial monomer concentrations between 3.2 and 4.7 mol L^–1^, proceeded readily, with quantitative conversion of the monomer within 20 h. Size exclusion chromatography (SEC) confirmed the polymeric nature of the products. As expected from free radical polymerizations, limited control and broad distributions were seen (*Đ*_M_ = 1.3–2.5), but higher molar mass polymers were obtained compared to solvent-free reactions (up to 5100 g mol^–1^; Table 1, entry 6). Molar mass limitations are likely due to a low rate of polymerization (*k*_p_), combined with frequent termination events and other side reactions. Polymeric series with expected repeat units of *m*/*z* 154, consistent with poly(**1**), were also detected by MALDI-ToF mass spectrometry, yet no end-group could be identified (Figure S50, for Table 1, entry 5). Compound **2** could also be polymerized under similar conditions, albeit to more moderate molar masses.

Initial insight into the structure of the polymers produced from **1** and **2** was gained by FT-IR spectroscopy. Compared to monomer **1** (Figure S19), poly(**1**) displayed new strong absorption bands at 1732 cm^–1^, corresponding to carbonyl vibrational modes while retaining strong acetal (C–O) frequencies at 1090 cm^–1^ (Figures S37, S46, and S47). The relative intensity of these vibrations varied depending on the polymerization reactions used and hinted at different microstructures, as later confirmed by NMR spectroscopy (*vide infra*). In contrast, the IR spectrum of poly(**2**) did not feature strong C=O stretching frequencies, with only a weak absorption band 1750 cm^–1^ (Figure S61) that could suggest traces of ester linkages, below the sensitivity of NMR spectroscopy (*vide infra*).

NMR analysis first revealed the retention of the internal alkene in poly(**1**) (δ_c_ = 124–129 ppm; δ_H_ = 5.57–5.98 ppm (2H), e.g., Figures S33 and S34), in line with the lack of reactivity of the pseudoglucal that we observed under free polymerization conditions. Then, ^13^C{^1^H} NMR spectroscopy was used to determine the microstructure of the polymer chains and the selectivity toward ester or acetal linkages within the polymer backbone (Figure 1). While both polymers showed evidence of carbohydrate moieties, the ^13^C{^1^H} NMR spectra of poly(**1**) indeed displayed three distinctive environments at δ_C_ ≈ 113, 170, and 172 ppm, corresponding to polyacetal (R_2_CO_2_) and polyester (C(O)O) linkages, respectively. DOSY NMR analysis (Figure S45) revealed the incorporation of both linkages within poly(**1**). The presence of two polyester environments was attributed to the possibility of either **1a** or **1b** radicals forming new C–C bonds from carbon atoms in position 4 or 2 or the pyranose ring, respectively. In stark contrast, poly(**2**) only displayed a solitary acetal environment at δ_C_ ≈ 101 ppm. Quantitative ^13^C{^1^H} NMR spectra were then generated to measure the relative intensities of the aforementioned signals.

**Figure 1:**
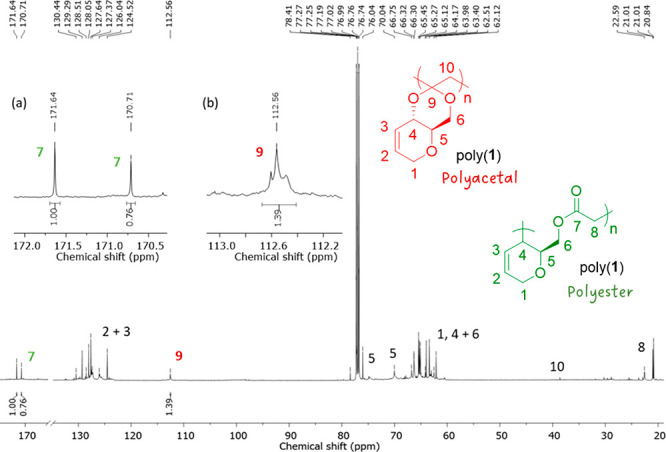
^13^C{^1^H} NMR spectrum (CDCl_3_) of poly(**1**): (a) ester (C(O)O) linkages and (b) acetal (R_2_CO_2_) linkages.

The polymerization of **2** only produced acetal linkages, while **1** always resulted in some ring fragmentation, demonstrating that ring-opening of **1**/**2** into primary alkyl radicals was not favored and confirming our hypothesis that the internal alkene functionality is essential to promote the formation of ester linkages, likely by stabilizing secondary radicals (Scheme S3). A minimum of 23% of ester linkages was seen in poly(**1**) when the reaction was performed with no solvent (Table 1, entry 1), and up to 78% of ester linkages were obtained under dilute conditions (Table 1, entry 2). Despite some variation between experiments due to a lack of polymerization control, decreasing [**1**]_0_ from 4.7 to 1.9 mol L^–1^ clearly favored the ROP of the CKA monomer and increased the ratio of ester linkages in poly(**1**) (Table 1, entries 2–6). However, below 1.9 mol L^–1^, the overall reactivity decreased, so that only oligomeric species were isolated.

In comparison to established CKA monomers, such as MPDO and BMDO, known for the propensity to undergo ring fragmentation, selectivity toward rROP and ester linkages is lower. However, against other six-membered rings or 2-methylene-1,3-dioxe-5-pene (which features an internal alkene function), this selectivity is comparable (Table S7).^11^ Nonetheless, further investigation of the polymerization process is required to achieve selective polyester formation. It is also worth noting that the occurrence of branching is a possibility that was not investigated here, and that linear architectures have been assumed for both polymers.^50^

Poly(**1**) and poly(**2**) were both shown to be amorphous by differential scanning calorimetry (DSC). Poly(**2**) displayed glass transition temperatures (*T*_g_s) of 83 and 93 °C for polymers of *M*_n_ 1000 g mol^–1^ (*Đ*_M_ 1.66) and 1400 g mol^–1^ (*Đ*_M_ 1.83), respectively (Table 1, entries 7–8; Figure S64). For poly(**1**), a correlation was identified between the *T*_g_ and the ratio of ester and acetal linkages (Table 1, Figure S51). *T*_g_ was reduced from 78 to 65 °C as the incorporation of esters increased from 23 to 78%, respectively (Figure 2a). Thermogravimetric analysis (TGA) also revealed thermal degradation (*T*_d5%_) to vary with the poly(**1**) structure (Table 1, Figures S52–S56). However, no trend was identified, even when considering variations in molar masses. In contrast, for poly(**2**), TGA revealed *T*_d5%_ to consistently occur at 214–215 °C (Figure S65).

**Figure 2:**
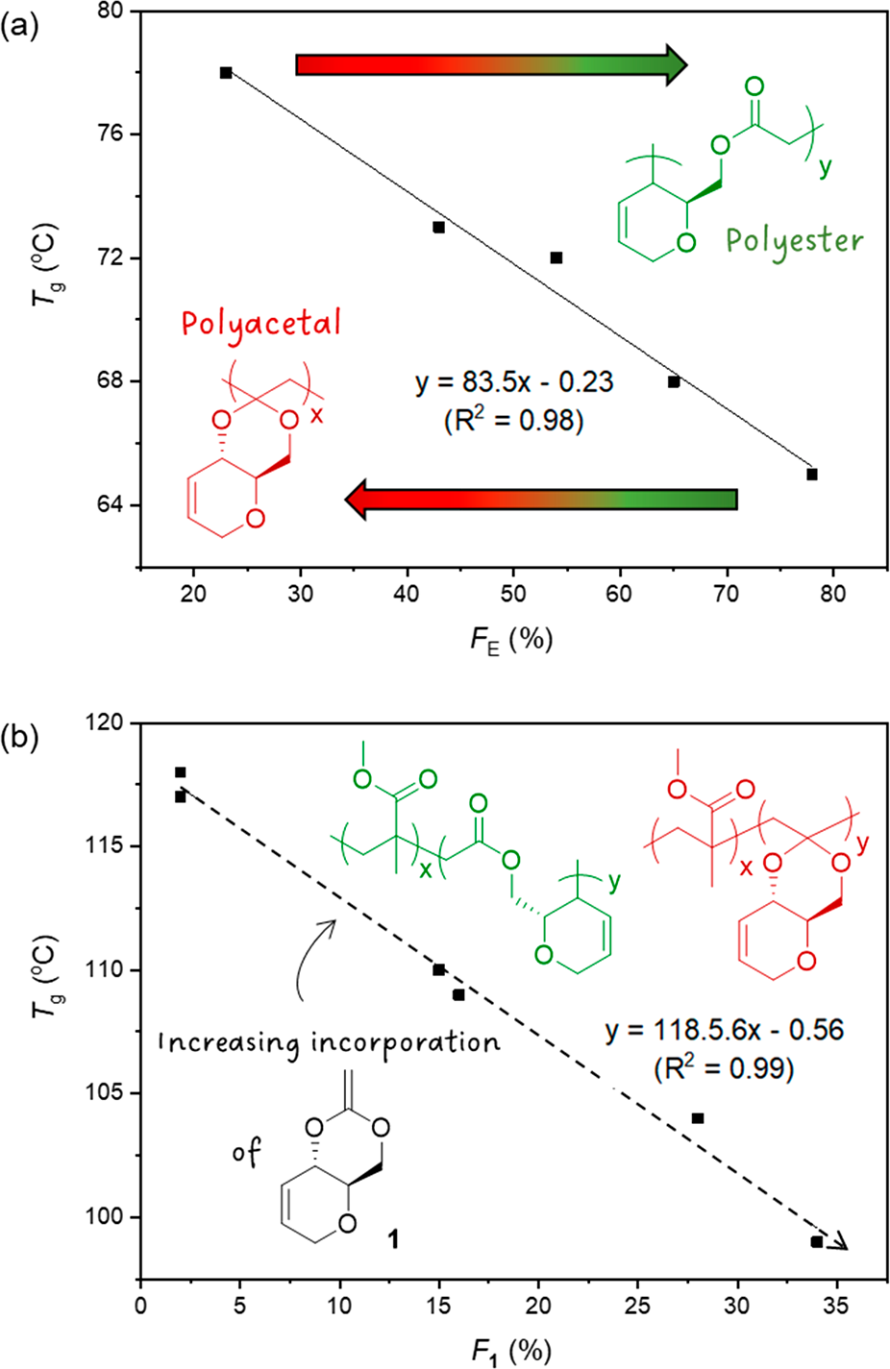
Glass transition temperature of (a) poly(**1**) vs the molar fraction of ester linkages in the polymer (*F*_E_); (b) poly(**1**-*co*-MMA) vs the molar fraction of **1** in the copolymer (*F*_**1**_).

Next, the one-pot copolymerization of **1** with various amount of methyl methacrylate (MMA) was investigated (Table 2). At 140 °C and a 100:1 [**M**_total_]_0_:[DTBP]_0_ ratio, MMA and **1** were quantitatively converted within 20 h. After precipitation in diethyl ether, DOSY NMR and SEC analysis revealed the formation of copolymers with high molar masses (*M*_n_ 12600–39900 g mol^–1^) and broad distributions (*Đ*_M_ 2.0–2.6), as expected from free radical polymerization. The carbonyl region of the copolymers’ ^13^C{^1^H} NMR spectra featured a cluster of MMA ester signals between δ_C_ 176–179 ppm, as well other quaternary resonances at δ_C_ 170–172 and 101–103 ppm, consistent with poly(**1**) ester and acetal linkages, respectively (Figures S68 and S69). Further analysis (not possible for the 10/90 *f*_**1**_/*f*_MMA_ copolymer due to the low intensity signal) revealed the copolymerization to produce predominantly acetal linkages, with a maximum of 36% of ester linkages observed for a 50/50 *f*_**1**_/*f*_MMA_ monomer feed (Table 2, entry). All copolymers also retained **1**’s internal alkene functionality (e.g., Figures S67 and S68). Despite both monomers being fully converted, the incorporation of **1** into the precipitated copolymer always proved to be lower than the feed. NMR (Figures S84–S86) and GPC (Figures S87–S89) analyses of the supernatant obtained postprecipitation revealed oligomeric species incorporating the remainder of **1**, sign of uncontrolled polymerization and termination events. Based on monomer incorporation, we hypothesize that MMA is more reactive than **1**. From the observation of multiple ester and acetal environments, we also believe a statistical copolymer structure is most likely.

**Table 2:**
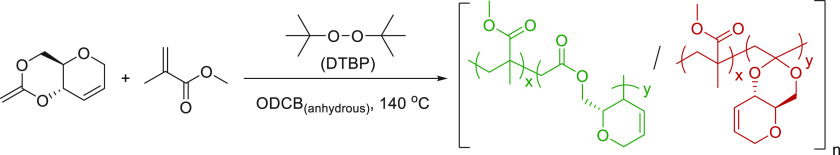
Radical Ring-Opening Copolymerization of **1** and MMA^a^

entry	*f***_1_**/*f*_MMA_	**1** conv.^b^ (%)	MMA conv.^c^ (%)	*F*_E_/*F*_A_^d^	*M*_n,SEC_^e^	*Đ*_M_^e^	*F***_1_**/*F*_MMA_^f^	yield^g^ (%)	*T*_g_^h^ (°C)	*T*_d5%_ (°C)
1	10/90	99	99		39.9	2.59	2/98	74	117	286
2	30/70	99	99	28/72	24.4	2.01	16/84	72	109	274
3	50/50	99	99	36/64	12.6	2.43	33/67	69	99	238

a Reactions were carried out over 20 h at 140 °C, under an argon atmosphere, in anhydrous ODCB with [**1**]_0_ = 4.8 mol L^–1^ and [**M**_total_]_0_:[DTBP]_0_ = 100:1.

b Monomer conversion to polymer, calculated based on the relative integration of the methylene proton signal of **1** (δ_H_ = 3.85, d, 2H) and the resultant alkyl proton signal of poly(**1**-*co*-MMA) (δ_H_ = 2.82–2.36, m, 2H), in the ^1^H NMR spectrum.

c Monomer conversion to polymer, calculated based on the relative integration of MMA and PMMA alkyl protons in the ^1^H NMR spectrum.

d Polymer composition between ester (*F*_E_) and acetal (*F*_A_) linkages, determined using ^13^C{^1^H} NMR spectroscopy when possible.

e Number-average molar mass and dispersity (*M*_n,SEC_, *Đ*_M_), calculated by SEC relative to polystyrene standards in THF eluent, units in kg mol^–1^.

f Copolymer compositions determined by integration of the ^1^H NMR spectra of the purified copolymers.

g Isolated polymer yield, oligomeric species were also separated during isolation.

h Values obtained from DSC second heating cycle.

All poly(**1**-*co*-MMA)s were shown to be amorphous by DSC analysis (Table 2, Figure S79). A linear trend could be identified between the *T*_g_s of the copolymers and the incorporation of **1** (Figure 2b). Thus, as the incorporation of **1** decreased from 33 to 2%, the *T*_g_ increased from 99 to 117 °C. TGA analysis revealed that the copolymers possessed excellent thermal stability with *T*_d5%_ between 238–286 °C (Table 2, Figures S80–S82) and although the incorporation of **1** led to a decrease in thermal stability, no obvious trend was identified. In comparison, the *T*_g_ and *T*_d,max_ of atactic PMMA, produced as part of the study via free radical polymerization, were 105 and 361 °C, respectively (*M*_n,SEC_ 75700 g mol^–1^; *Đ*_M_ 1.82; Figures S93 and S94).

Building on previous studies surrounding the degradation of statistical copolymers between vinylic monomers and CKAs,^20,25−27,51^ the hydrolytic degradation of poly(**1**-*co*-MMA) was investigated (Table 3). Dissolved in THF, poly(**1**-*co*-MMA) could be readily degraded via the addition of NaOH/KOH (1 mol L^–1^ in H_2_O or MeOH) at room temperature. After 4 h, the reaction mixtures were analyzed by SEC and a decrease in molar mass was observed for each copolymer, which correlated well with the quantity of **1** incorporated. Analysis of the degradation products proved difficult due to their limited solubility, although ^1^H NMR spectroscopy pointed toward a breakdown of the polymer at the CKA units, highlighted by the broadening of sugar signals and intact PMMA species (Figures S96–S99).

**Table 3:**
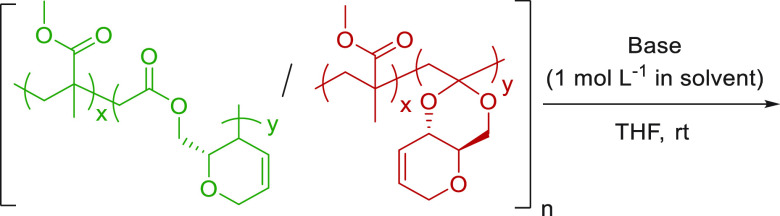
Hydrolytic Degradation of Poly(**1**-*co*-MMA)

entry	*F***_1_**^b^	base	solvent	final *M*_n,SEC_ [*Đ*_M_]^c^	% *M*_n_ change^d^
1	0	NaOH	H_2_O	77.1 [1.83]	0
2	0	NaOH	MeOH	76.7 [1.83]	0
3	0.16	NaOH	H_2_O	19.9 [1.72]	18
4	0.16	NaOH	MeOH	16.2 [2.72]	34
5	0.16	KOH	MeOH	12.1 [1.76]	50
6	0.33	NaOH	H_2_O	6.3 [2.31]	50
7	0.33	NaOH	MeOH	3.0 [1.67]	78
8	0.33	KOH	MeOH	1.3 [1.88]	90

a Degradations were performed for 4 h at room temperature.

b Molar fraction of **1** incorporated into poly(**1**-*co*-MMA).

c Number-average molar mass and dispersity (*M*_n,SEC_, *Đ*_M_), calculated by SEC relative to polystyrene standards in THF eluent, units given in kg mol^–1^.

d Percentage variation compared to the (co)-polymer original *M*_n,SEC_.

Control experiments were also carried out. Under identical conditions, SEC revealed that poly(**1**) is fully degraded to oligomeric products (Figure S95). In contrast, no change was observed for PMMA, supporting the hypothesis that degradation is due to the CKA content of the copolymer and that degradable PMMA materials have been achieved. Under basic conditions, it is likely that the chain cleavage takes place at the ester linkages. It is, however, worth noting that degradation of acetal linkages has been shown to be possible via acid hydrolysis.^52,53^

In summary, two novel sugar-derived CKA monomers possessing either a saturated or an unsaturated pyranose ring have been synthesized. Their ring-opening polymerization has been investigated, and the resulting polymers characterized. Confirming our original hypothesis, the alkene functionality of d-glucal is necessary toward ring-fragmentation and the formation of ester linkages. This unsaturated monomer was also copolymerized with MMA toward the formation of hydrolytically degradable statistical copolymers, with minimal disruption of PMMA thermal properties. This strategy, although effective, still requires further investigation to enable higher incorporation of ester linkages and subsequently further degradability of PMMA materials. Importantly, **1** is a rare example of a CKA monomer that bears an additional alkene function and polymerizes readily (as opposed to, for example, 2-methylene-1,3-dioxe-5-pene^54^). Evidence suggests that throughout polymerization this functionality remains untouched, opening avenues for combining degradability with postpolymerization functionalization^45^ and fine-tuning of the material properties, including degradability.

## Supporting Information Available

Experimental methods and synthetic procedures; Spectroscopic and crystallographic data for **1**, **2**, and intermediates; SEC traces, spectroscopic and thermal (TGA, DSC, DMA) data for the polymers; MALDI-ToF spectrum for poly(**1**) (PDF)
CCDC 2234496 (CIF)

## Supplementary Material

mz3c00397_si_001.pdf

mz3c00397_si_002.cif
