# Supplementary material for: Radical Ring Opening Polymerization of Cyclic Ketene Acetals Derived From d-Glucal
Source: ACS Macro Lett. 2023 Oct 12;12(11):1443–9. doi: 10.1021/acsmacrolett.3c00397 (PMC10666543; doi:10.1021/acsmacrolett.3c00397)
Supplement: Supplementary file 1 — mz3c00397_si_001.pdf [file mz3c00397_si_001.pdf]

Supporting Information for

Radical Ring Opening Polymerization of Cyclic Ketene Acetals Derived From D-Glucal

Craig A. Hardy,[a] Martin E. Levere,[b] Gabriele Kociok-Köhn[b] and Antoine Buchard*[a,c]

[a]Department of Chemistry, University of Bath, Claverton Down, Bath BA2 7AY, UK.

[b]Materials and Chemical Characterisation Facility (MC2), University of Bath, Claverton Down, Bath, BA2 7AY, UK.

[c]University of Bath Institute for Sustainability, Claverton Down, Bath, BA2 7AY, UK.

Email: a.buchard@bath.ac.uk

Table of Contents

Materials and Methods **S4**

General Procedures and Analytical Data **S6**

1. **Synthesis and Characterization of Cyclic Ketene Monomer, 1** **S6**

   Synthesis of (2R,3S)-3-acetoxy-3,6-dihydro-2H-pyran-2-yl)methyl acetate S6

   NMR analysis of (2R,3S)-3-acetoxy-3,6-dihydro-2H-pyran-2-yl)methyl acetate S7

   Synthesis of (2R,3S)-2-(hydroxymethyl)-3,6-dihydro-2H-pyran-3-ol S8

   NMR analysis of (2R,3S)-2-(hydroxymethyl)-3,6-dihydro-2H-pyran-3-ol S9

   Synthesis of (4aR,8aS)-2-(bromomethyl)-4,4a,6,8a-tetrahydropyrano-[3,2-d][1,3]-dioxine S10

   NMR analysis of (4aR,8aS)-2-(bromomethyl)-4,4a,6,8a-tetrahydropyrano-[3,2-d][1,3]-dioxine S11

   Crystal diffraction data and structure refinement for (4aR,8aS)-2-(bromomethyl)-4,4a,6,8a-tetra-hydropyrano-[3,2-d][1,3]-dioxine (CCDC Number - 2234496) S14

   Synthesis of (4aR,8aS)-2-methylene-4,4a,6,8a-tetrahydropyrano[3,2-d][1,3]dioxine (**1**) S16

   NMR analysis of (4a*R*,8a*S*)-2-methylne-44a,6,8a-tetrahydropyrano[3,2-*d*][1,3]dioxine (**1**) S16

   FT-IR analysis of (4a*R*,8a*S*)-2-methylne-44a,6,8a-tetrahydropyrano[3,2-*d*][1,3]dioxine (**1**) S19

2. **Synthesis and Characterization of Cyclic Ketene Monomer, 2** **S20**

   Synthesis of (2R,3S)-2-(hydroxymethyl)tetrahydro-2H-pyran-3-ol S20

   NMR analysis of (2R,3S)-2-(hydroxymethyl)tetrahydro-2H-pyran-3-ol S21

   Synthesis of (4aR,8aS)-2-(bromomethyl)hexahydropyrano[3,2-d][1,3]dioxine S23

   NMR analysis of (4aR,8aS)-2-(bromoethyl)hexahydropyrano[3,2-d][1,3]dioxine S23

   Synthesis of (4aR,8aS)-2-methylene-4,4a,6,8a-tetrahydropyrano[3,2-d][1,3]dioxine (**2**) S26

   NMR analysis of (4aR,8aS)-2-methylene-4,4a,6,8a-tetrahydropyrano[3,2-d][1,3]dioxine (**2**) S26

3. **Free radical polymerization of 1 (solvent-free conditions)** **S29**

   NMR analysis of the poly(**1**) (solvent-free conditions) S30

   FT-IR analysis of poly(**1**) (solvent-free conditions) S32

4. **Free radical polymerization of 1 (in solution)** **S33**

   NMR analysis of poly(**1**) (solution polymerization) S34

   FT-IR analysis of poly(**1**) (solution polymerization) S38

   Representative size-exclusion chromatography analysis of poly(**1**) S39

   MALDI ToF MS analysis of poly(**1**) S40

5. **Thermal analysis of poly(1)** **S41**

6. **Free radical polymerization of 2 (in solution)** **S45**

   NMR analysis of poly(**2**) (solution polymerization) S45

   FT-IR analysis of poly(**2**) (solution polymerization) S47

   Representative size-exclusion chromatography analysis of poly(**2**) S48

   Thermal analysis of poly(**2**) S48

7. **One-pot copolymerization of 1 with methyl methacrylate** **S50**

   NMR analysis of poly(**1**-*co*-MMA) S51

   FT-IR analysis of poly(**1**-*co*-MMA) S56

   Representative size-exclusion chromatography analysis of poly(**1**-*co*-MMA) S57

Thermal analysis of poly(**1**-*co*-MMA) S58
NMR analysis of poly(**1**-*co*-MMA) supernatant S60
Representative size-exclusion chromatography analysis of poly(**1**-*co*-MMA) supernatant S62
Thermal analysis of poly(**1**-*co*-MMA) supernatant S63
Thermal analysis of PMMA S65
8. Polymer degradation S67
Hydrolytic degradation of poly(**1**) S67
Hydrolytic degradation of poly(**1**-*co*-MMA) S67
Complementary data S72
Mechanistic considerations S72
Selectivity towards ROP of common CKA monomers S73
References S74

Materials and Methods

Unless otherwise stated, all starting materials and reagents were obtained from Sigma-Aldrich, Acros Organics or Alfa Aesar and used without further purification. All solvents were obtained from either Fisher Scientific or VWR Chemicals, except for anhydrous solvents, which were purchased from Sigma-Aldrich or Acros Organics and used without further purification. Tri-*O*-acetyl-D-glucal was purchased from Carbosynth. Where appropriate, the progress of reactions was monitored by thin layer chromatography using silica coated aluminium plates (Kieselgel 60G F254) purchased from VWR Chemicals and visualized using a potassium permanganate ($KMnO_4$) stain. The purification of intermediates and final products was accomplished by flash column chromatography, using silica gel (Fluka, pore size 60 Å, 70-230 mesh, 63-200 μm), and the purity of the final compounds was determined by NMR spectroscopy.

NMR spectra were primarily recorded on a Brucker-400/500 spectrometer operating at a frequencies of 400/500 MHz (^{1}H) and 101/126 MHz (^{13}C). The NMR spectra were recorded in $CDCl_3$, relative to reference points of the deuterated solvent. Chemical shifts (δ) are quoted in ppm and coupling constants (*J*) are quoted in Hertz. Abbreviations used to describe the multiplicity of the peaks observed are defined as follows: s = singlet, d = doublet, t = triplet, m = multiplet, dd = doublet of doublets and so on. A Bruker Avance III (500 MHz) instrument with nitrogen-cooled Prodigy cryoprobe was used to generate quantitative spectra of the polymers and copolymers: pulse sequence - zgpg30, temperature - 298 K, number of scans - 8192, receiver gain - 191.2 bits, relaxation delay - 2.000, acquisition time - 1.1010 sec and spectral width - 29761.9 Hz.

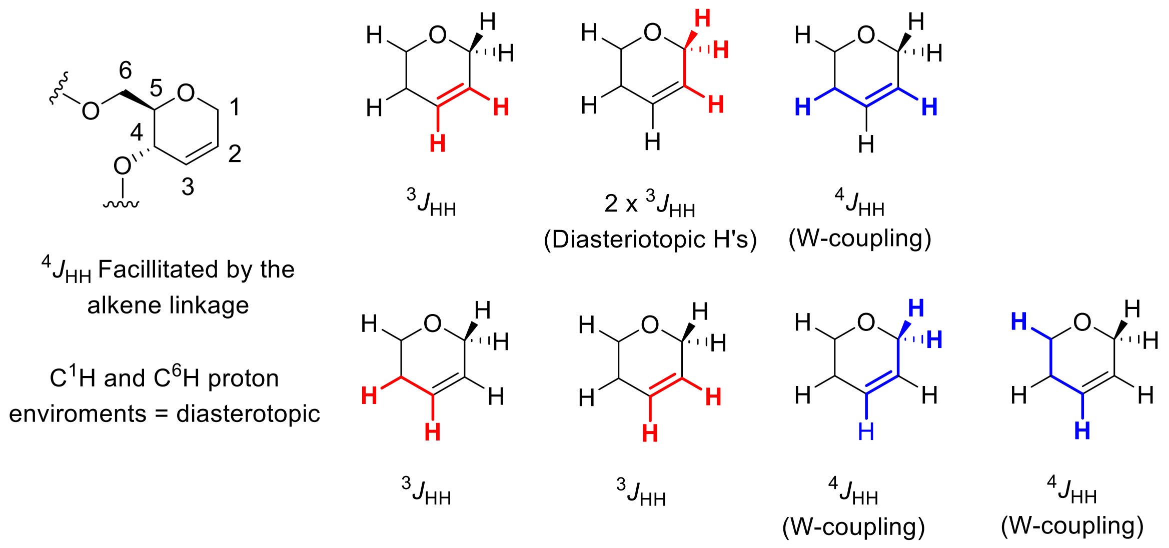

Figure S1. Depiction of possible coupling constants for the C^{2}H and C^{3}H proton environments, based upon splitting patterns observed within recorded ^{1}H NMR spectra.

Size-exclusion chromatography (SEC) was carried out using THF eluent. Multi analysis software was used to process the data. Polymer samples were dissolved at a concentration of 1 mg mL^{-1}. Samples were recorded on an Agilent 1260 Infinity series instrument at 1 mL min^{-1} at 35 °C using two PLgel 5 μm MIXED-D 300 × 7.5 mm columns in series. Samples were detected with a differential refractive index (RI) detector.

Number-average molecular weight ($M_{n,SEC}$), and dispersities, ($Đ_M$ (M_w/M_n)) were calculated against a polystyrene calibration (11 polystyrene standards of narrow molecular weight, ranging from M_w 615–568000 Da).

Differential scanning calorimetry (DSC) was carried out using a MicroSC multicell calorimeter from Setaram; the Calisto program was employed to collect and process the data. The measurement cell and the reference cell were both a 1 mL Hastelloy C cell; a mass of 2–5 mg of polymeric material was loaded into the measurement cell with the reference cell empty. The experiments were performed under N_2 and the sample heated and cooled at a rate of 10 K min^{-1} unless otherwise stated. A second heating and cooling cycle was carried out immediately following completion of the first, unless otherwise stated. Data was plotted using Origin 2018.

Thermogravimetric analysis (TGA) was carried out using A Setsys Evolution TGA 16/18 from Setaram; the Calisto program was employed to collect and process the data. The sample was loaded into a 170 μL alumina crucible and the analytical chamber purged with argon (200 mL min^{-1}) for 20 minutes prior to starting the analysis. The sample was then heated under an argon flow (20 mL min^{-1}) from 30 to 600 °C at a rate of 10 °C min^{-1}, unless otherwise stated.

FT-IR analysis was carried out using a PerkinElmer Inc. Spectrum 100 FT-IR Spectrometer. Universal ATR enabling wavelengths from 650-4000 cm−1 (15 μm to 2.5 μm). Data was plotted using Origin 2018.

Single-Crystal X-ray Diffraction (XRD) analysis was carried out by Dr Gabriele Kociok-Kohn on a Nonius Kappa CCD diffractometer using Cu-K$_\alpha$ radiation (λ = 1.54184 Å) at 150 K.

MALDI-TOF Mass Spectra (MALDI ToF MS) were recorded on a Bruker Autoflex Speed MALDI-TOF Mass Spectrometer equipped with a 2 kHz SmartBeam-II laser, and samples were loaded on a 384-well ground steel plate. The spectra were analysed using Autoflex analysis software. MS Spectra were recorded in positive ion reflectron mode. The instrument was calibrated with peptide standards (Bruker Peptide Calibrant II) ranging from m/z 1046.54 to 3147.47. A solution of trans-2-[3(4-tert-Butylphenyl)-2-methyl-2propenylidene (DCTB) in THF was prepared at a concentration of 15 mg mL^{-1}; a solution of polymer in THF was prepared at 15mg mL^{-1}; and a solution of sodium trifluoroacetate in THF was prepared at 6 mg mL^{-1} in Eppendorf tubes. These three solutions were then mixed in a 5/2/1 (v/v/v) ratio in a Eppendorf tube and 0.5 μL spotted onto a ground steel MALDI-TOF MS plate to dry before being inserted into the instrument chamber. The sample was analysed in linear positive ionisation mode and laser power, detector gain and detection range adjusted to obtain and optimal spectrum from the summed spectra. The substrate did not ionise easily, requiring 100% laser power and a high detector gain setting. The data was analysed using the Flex Analysis software, version 3.4 (build 76). The molar mass distributions were obtained through analysis of the data in the Polytools software package 1.31.

General Procedures and Analytical Data

1. Synthesis and Characterization of Cyclic Ketene Monomer, 1

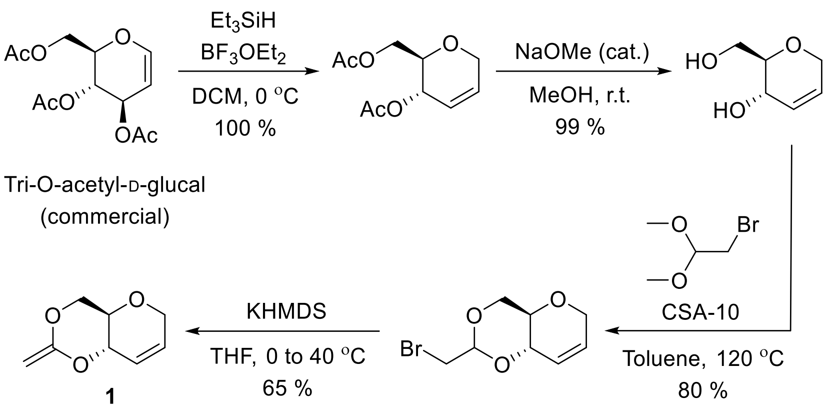

Scheme S1. Synthesis of CKA monomer **1**.

Synthesis of (2R,3S)-3-acetoxy-3,6-dihydro-2H-pyran-2-yl)methyl acetate

A solution of tri-O-acetyl-D-glucal (12.0 g, 44.1 mmol, 1.0 equiv.) in anhydrous DCM (50 mL) was cooled to 0 °C, under argon, and treated with triethylsilane (8.45 mL, 52.9 mmol, 1.2 equiv.), followed by $BF_3 \cdot OEt_2$ (ca. 48%, BF_3) (5.6 mL, 44.1 mmol, 1.0 equiv.). The reaction was stirred at 0 °C for 3 h. Once complete the reaction was quenched with $NaHCO_3$ (1 mol L^{-1}, 100 mL) and the organic phase was extracted using DCM (2 x 50 mL), washed with brine (100 mL) and water (2 x 100 mL), dried over $MgSO_4$, and the solvent was concentrated in *vacuo* to yield a colourless oil, which was used directly in the next reaction without further purification (9.44 g, 100 %). *Spectroscopic data was consistent with the literature.*[1]

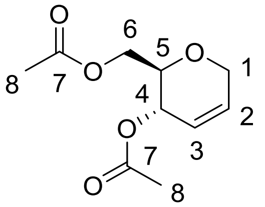

δ_H (400 MHz; chloroform-d): 5.93 (C^3H, 1H, dddd, J_{HH} = 10.3, 2.6, 2.5, 1.8 Hz), 5.74 (C^2H, 1H, dddd, J_{HH} = 10.3, 2.3, 2.3, 2.2 Hz), 5.28-5.19 (C^4H, 1H, m), 4.23-4.18 (C^1H, C^6H, 3H, m), 4.16 (C^6H, 1H, dd, J_{HH} = 12.1, 5.9 Hz), 3.71 (C^5H, 1H, ddd, J_{HH} = 8.6, 5.9, 2.9 Hz), 2.08 (C^7H, 3H, s), 2.06 (C^7H, 3H, s) ppm; δ_C (101 MHz; chloroform-d): 171.0 (C^7), 170.4 (C^7), 129.6 (C^3), 124.3 (C^2), 73.9 (C^5), 65.4 (C^4), 65.2 C^1), 63.4 (C^6), 21.2 (C^8), 20.9 (C^8) ppm.

NMR analysis of (2R,3S)-3-acetoxy-3,6-dihydro-2H-pyran-2-yl)methyl acetate

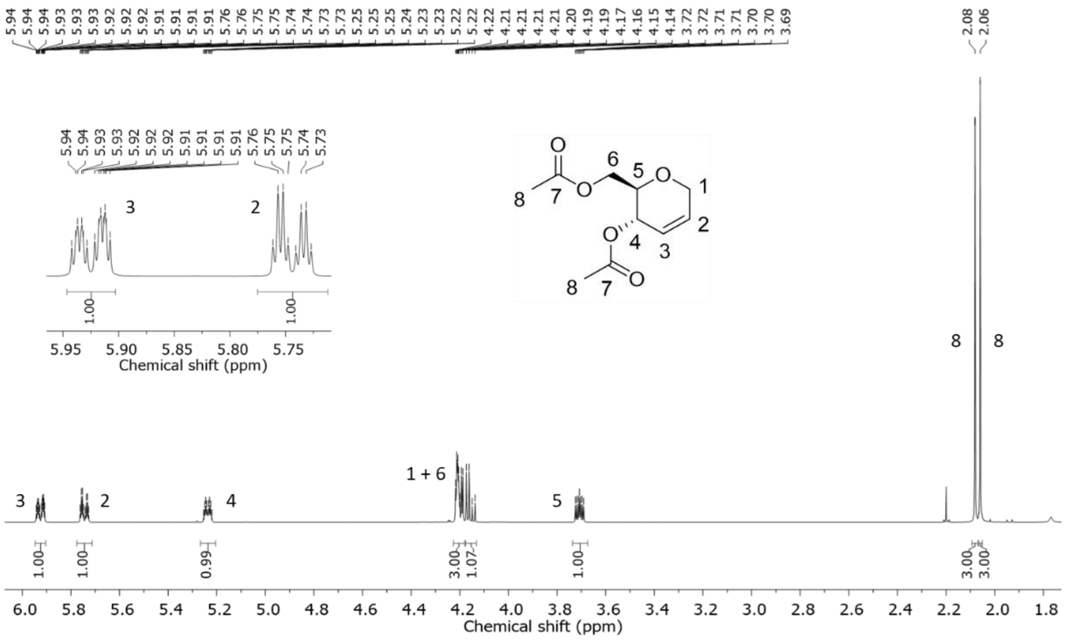

Figure S2. Annotated ^{1}H NMR spectrum ($CDCl_3$) of (2R,3S)-3-acetoxy-3,6-dihydro-2H-pyran-2-yl)methyl acetate.

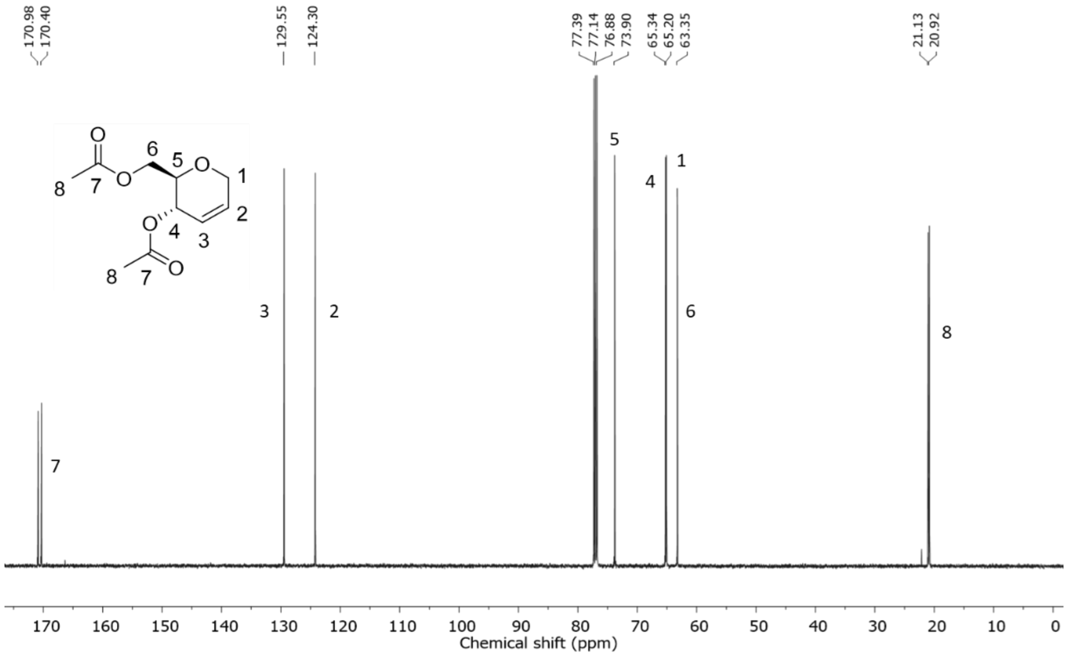

Figure S3. Annotated $^{13}C\{^{1}H\}$ NMR spectrum ($CDCl_3$) of (2R,3S)-3-acetoxy-3,6-dihydro-2H-pyran-2-yl)methyl acetate.

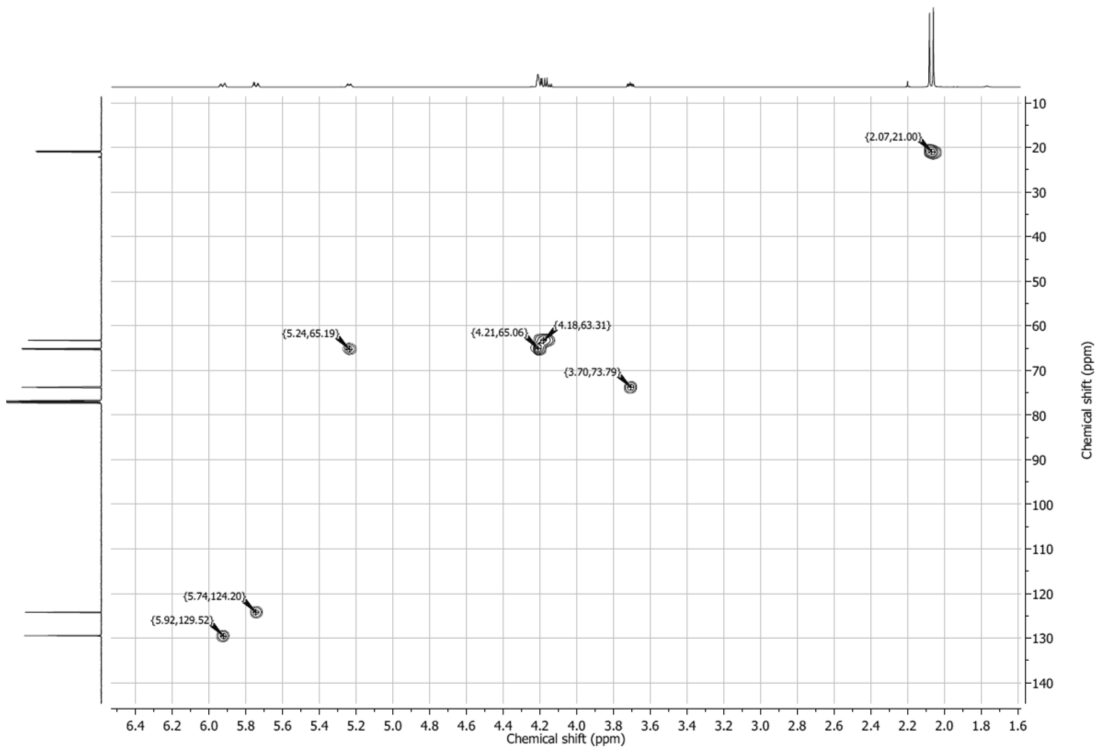

Figure S4. HSQC ($^1H–^{13}C$) NMR spectrum ($CDCl_3$) of (2R,3S)-3-acetoxy-3,6-dihydro-2H-pyran-2-yl)methyl acetate.

Synthesis of (2R,3S)-2-(hydroxymethyl)-3,6-dihydro-2H-pyran-3-ol

A solution of (2R,3S)-3-acetoxy-3,6-dihydro-2H-pyran-2-yl)methyl acetate (9.40 g, 43.9 mmol, 1.0 equiv.) in anhydrous methanol (50 mL) was treated with a solution of NaOMe (0.237 g, 4.39 mmol, 0.1 equiv.) in anhydrous methanol (2 mL) under argon and stirred at room temperature for 3 h. Once complete, ammonium chloride (0.47 g, 8.78 mmol, 0.2 equiv.) was added to the reaction and stirred for 15 mins. The reaction mixture was concentrated in vacuo, and then diluted with chloroform (100 mL), the resultant precipitate was removed by filtration, and the filtrate was concentrated in vacuo to yield a pale-yellow oil (5.68 g, 99 %). *Spectroscopic data was consistent with the literature*.[1]

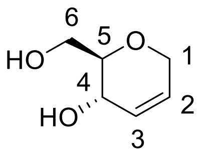

δ_H (400 MHz; chloroform-d): 5.91-5.74 (C^2H, C^3H, 2H, m), 4.24-4.12 (C^1H, C^4H, 3H, m), 3.89 (C^6H, 1H, dd, J_{HH} = 11.6, 3.8 Hz), 3.80 (C^6H, 1H, dd, J_{HH} = 11.6, 5.5 Hz), 3.34 (C^5H, 1H, ddd, J_{HH} = 8.1, 5.4, 3.9 Hz) ppm; δ_C (101 MHz; chloroform-d): 128.8 (C^3), 127.8 (C^2), 78.8 (C^5), 65.6 (C^4), 64.2 (C^1), 63.1 (C^6) ppm.

NMR analysis of (2R,3S)-2-(hydroxymethyl)-3,6-dihydro-2H-pyran-3-ol

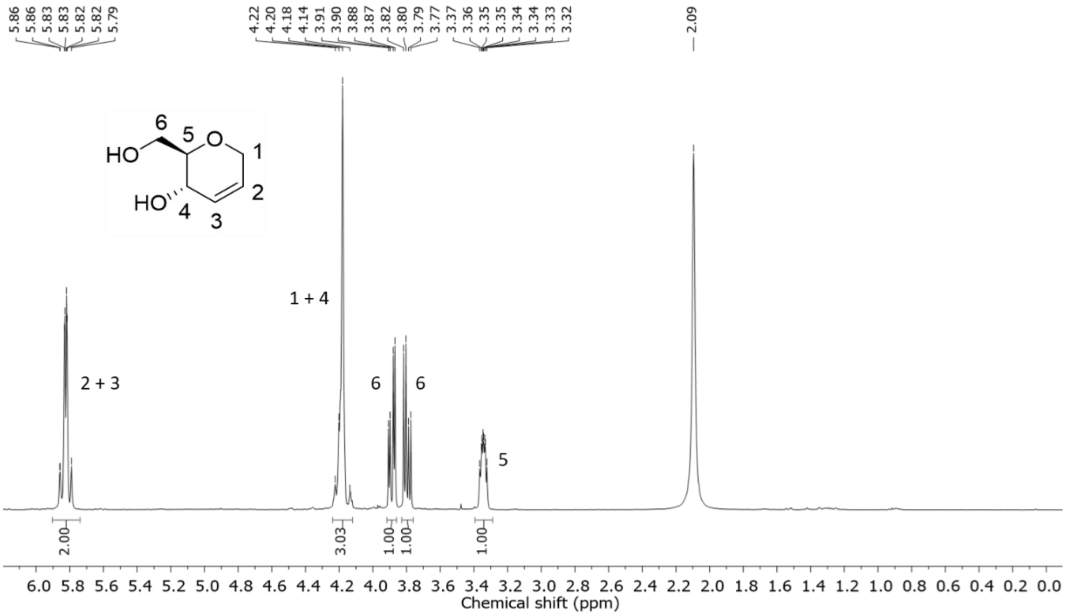

Figure S5. Annotated ^{1}H NMR spectrum ($CDCl_3$) of (2R,3S)-2-(hydroxymethyl)-3,6-dihydro-2H-pyran-3-ol.

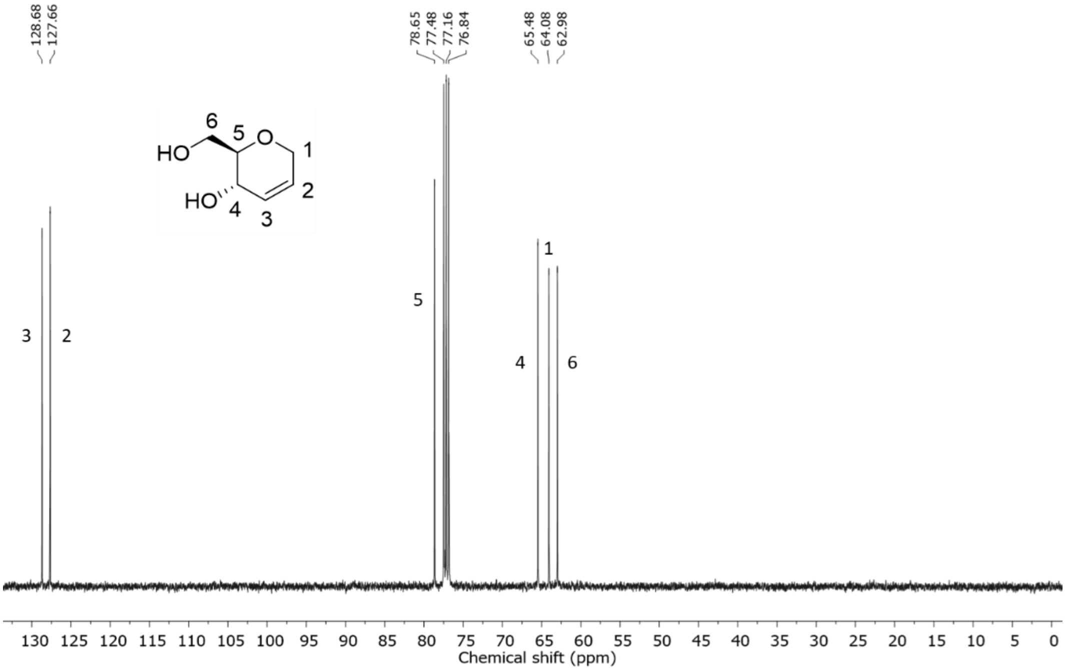

Figure S6. Annotated $^{13}C\{^{1}H\}$ NMR spectrum ($CDCl_3$) of (2R,3S)-2-(hydroxymethyl)-3,6-dihydro-2H-pyran-3-ol.

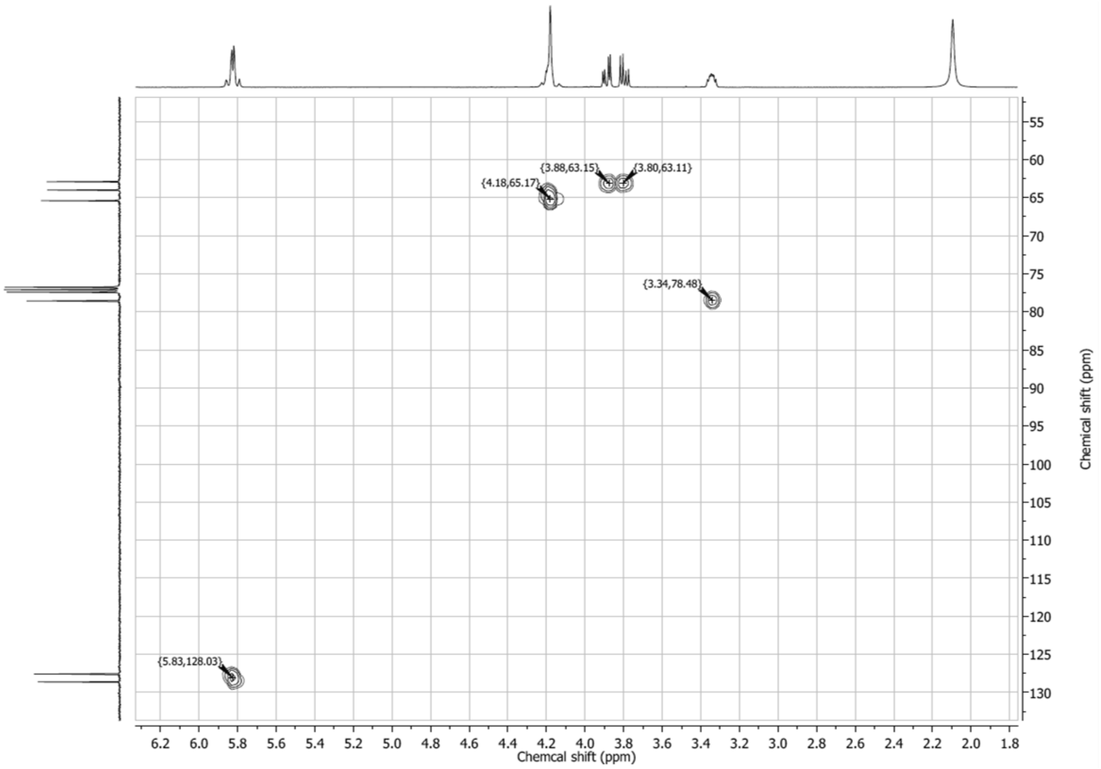

Figure S7. HSQC (^1H–^{13}C) NMR spectrum ($CDCl_3$) of (2R,3S)-2-(hydroxymethyl)-3,6-dihydro-2H-pyran-3-ol.

Synthesis of (4aR,8aS)-2-(bromomethyl)-4,4a,6,8a-tetrahydropyrano-[3,2-d][1,3]-dioxine

A solution of 2-bromo-1,1-dimethoxyethane (6.19 mL, 52.4 mmol, 1.2 equiv.) and (2R,3S)-2-(hydroxy-methyl)-3,6-dihydro-2H-pyran-3-ol (5.68 g, 43.6 mmol, 1.0 equiv.) in toluene (10 mL) was treated with (1*S*)-(+)-10-Camphorsulfonic acid (1.01 g, 4.36 mmol, 0.1 equiv.) and heated to 120 °C. The reaction was stirred for 6 h, and a black solution was formed. Once complete, the solvent was removed in *vacuo* to yield a black solid that was subsequently purified *via* column chromatography on SiO_2 using a hexane:EtOAc (4:1) mobile phase, fractions containing the product were combined and concentrated in *vacuo* to a yield pale yellow solid once cooled (8.22 g, 80 %).

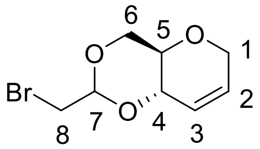

δ_H (500 MHz; chloroform-d): 5.94-5.86 (C^3H, 1H, m), 5.76 (C^2H, 1H, dddd, J_{HH} = 10.4, 2.7, 2.4, 2.4 Hz), 4.83 (C^7H, 1H, t, $^3J_{HH}$ = 4.5 Hz), 4.35-4.26 (C^1H, 1H, m), 4.24-4.18 (C^1H, C^6H, 2H, m), 4.05-4.01 (C^4H, 1H, m), 3.61 (C^6H, 1H, dd, J_{HH} = 10.4, 10.4 Hz), 3.42-3.36 (C^5H, C^8H, 3H, m) ppm. δ_C (126 MHz; chloroform-d): 128.1 (C^3), 125.9 (C^2), 100.4 (C^7), 75.2 (C^4), 70.2 (C^5), 69.4 (C^6), 66.7 (C^1), 31.2 (C^8) ppm.

NMR analysis of (4aR,8aS)-2-(bromomethyl)-4,4a,6,8a-tetrahydropyrano-[3,2-d][1,3]-dioxine

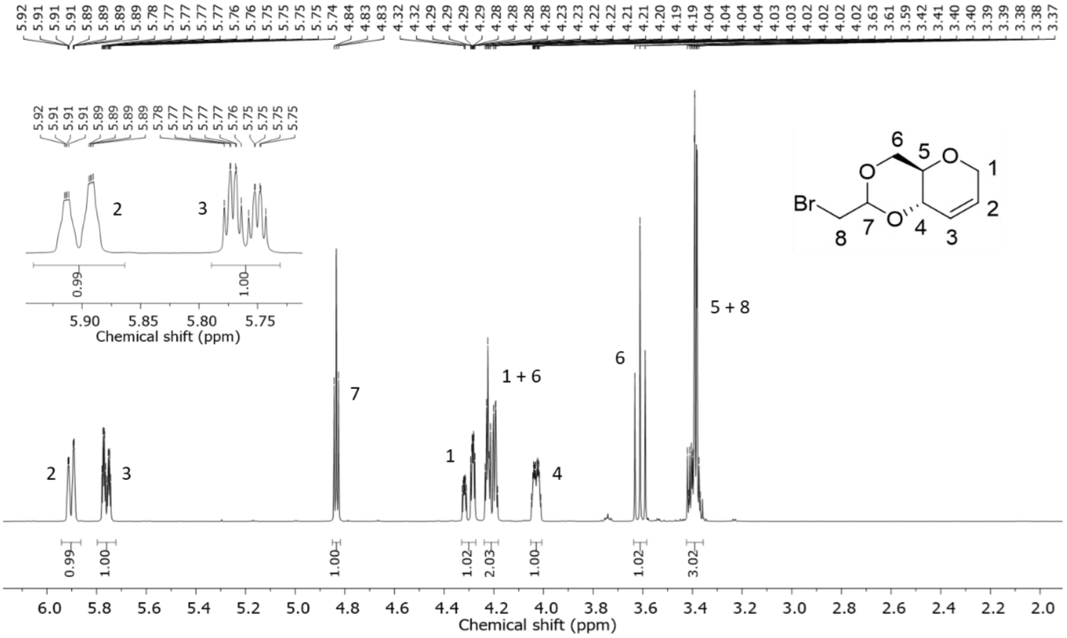

Figure S8. Annotated ^{1}H NMR spectrum ($CDCl_3$) of (4aR,8aS)-2-(bromomethyl)-4,4a,6,8a-tetrahydro-pyrano-[3,2-d] [1,3]-dioxine.

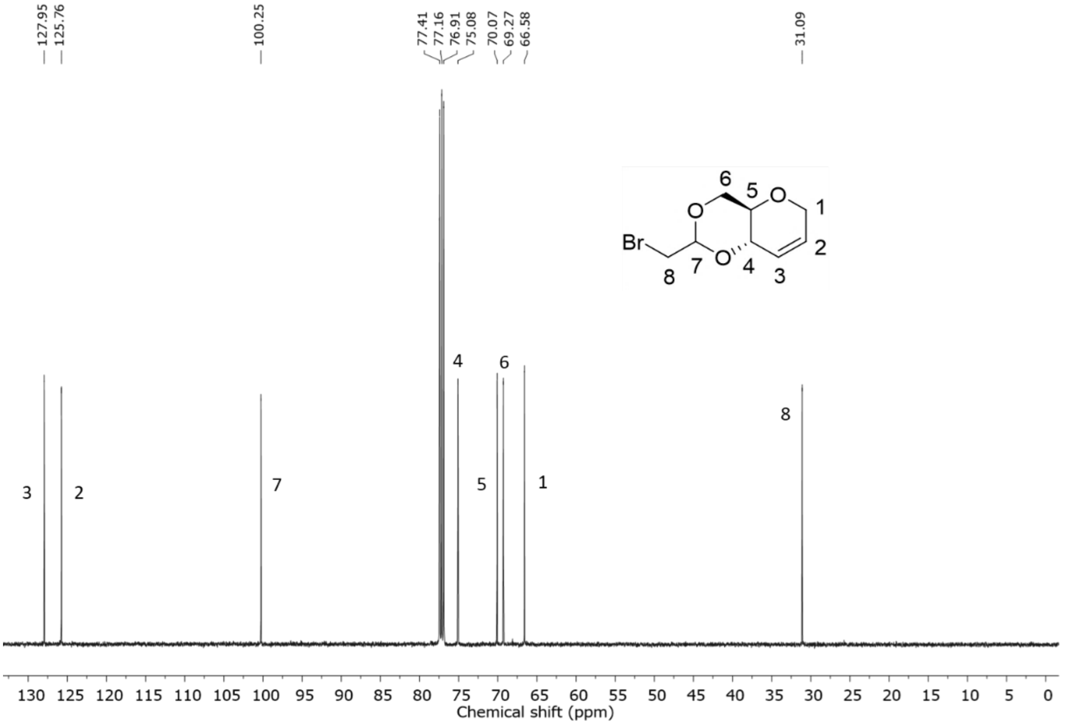

Figure S9. Annotated ^{13}C{^{1}H NMR spectrum ($CDCl_3$) of (4aR,8aS)-2-(bromomethyl)-4,4a,6,8a-tetrahydro-pyrano-[3,2-d][1,3]-dioxine.

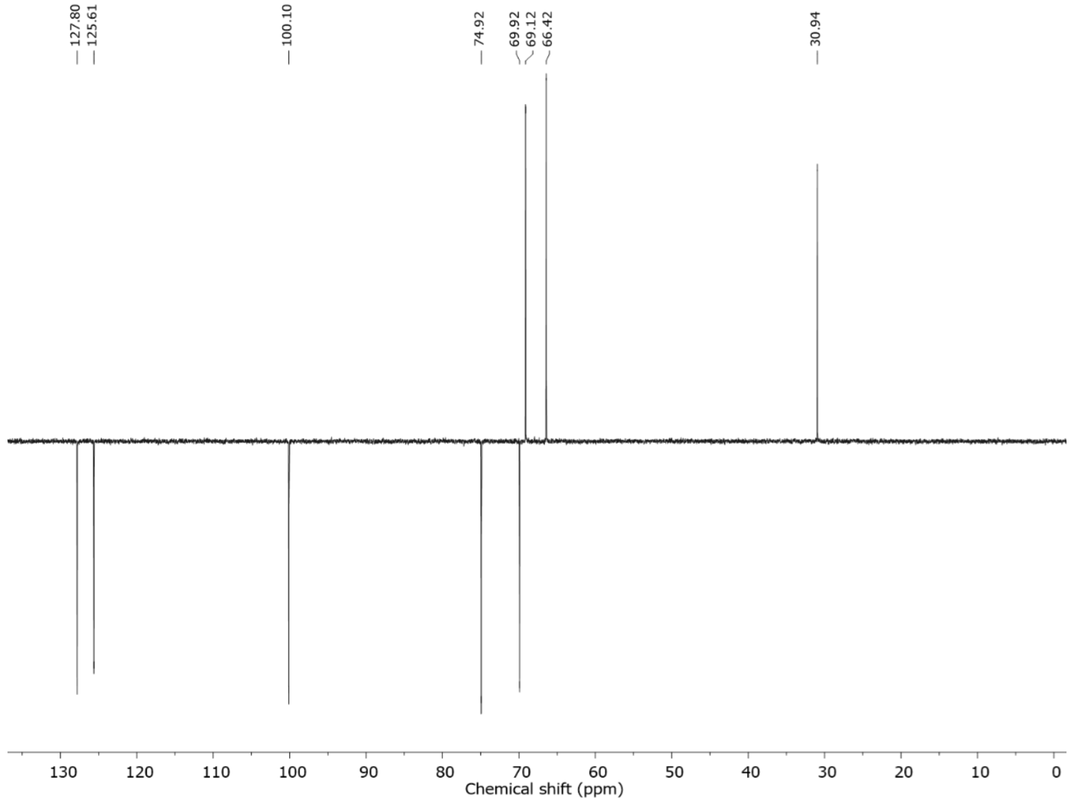

Figure S10. $^{13}C\{^{1}H\}$ DEPT135 NMR spectrum ($CDCl_3$) of (4aR,8aS)-2-(bromomethyl)-4,4a,6,8a-tetrahydro-pyrano-[3,2-d][1,3]-dioxine.

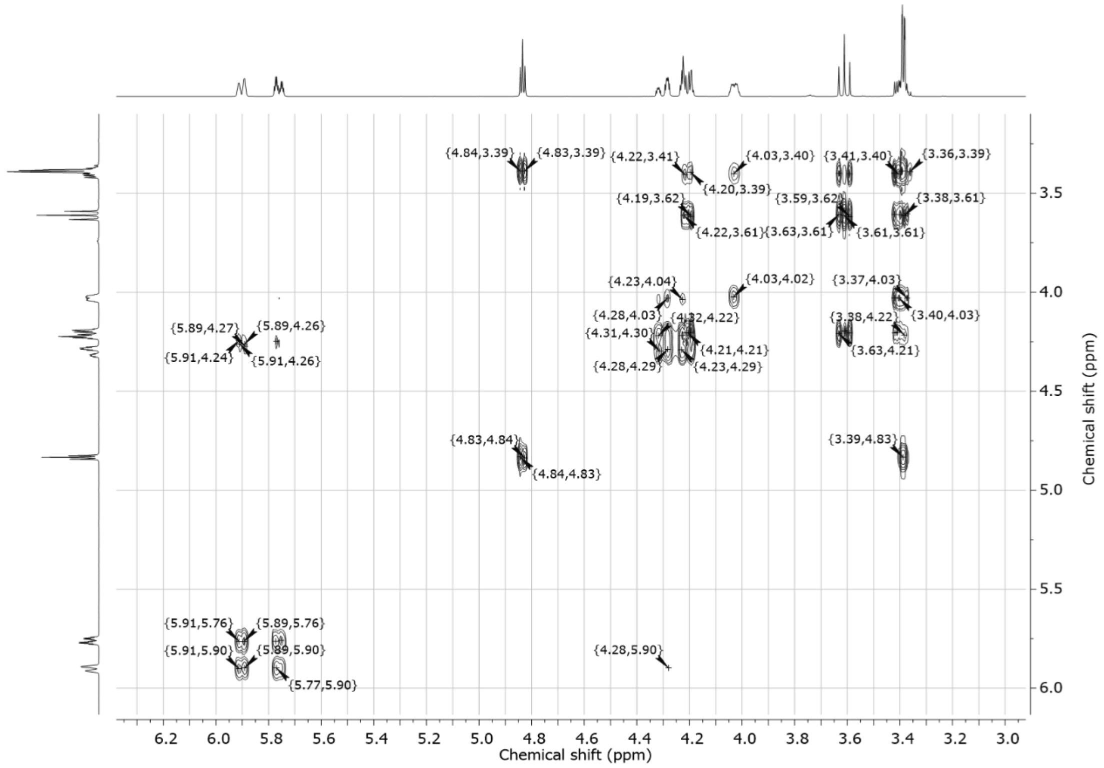

Figure S11. COSY (^{1}H–^{1}H) NMR spectrum ($CDCl_3$) of (4aR,8aS)-2-(bromomethyl)-4,4a,6,8a-tetrahydro-pyrano-[3,2-d][1,3]-dioxine.

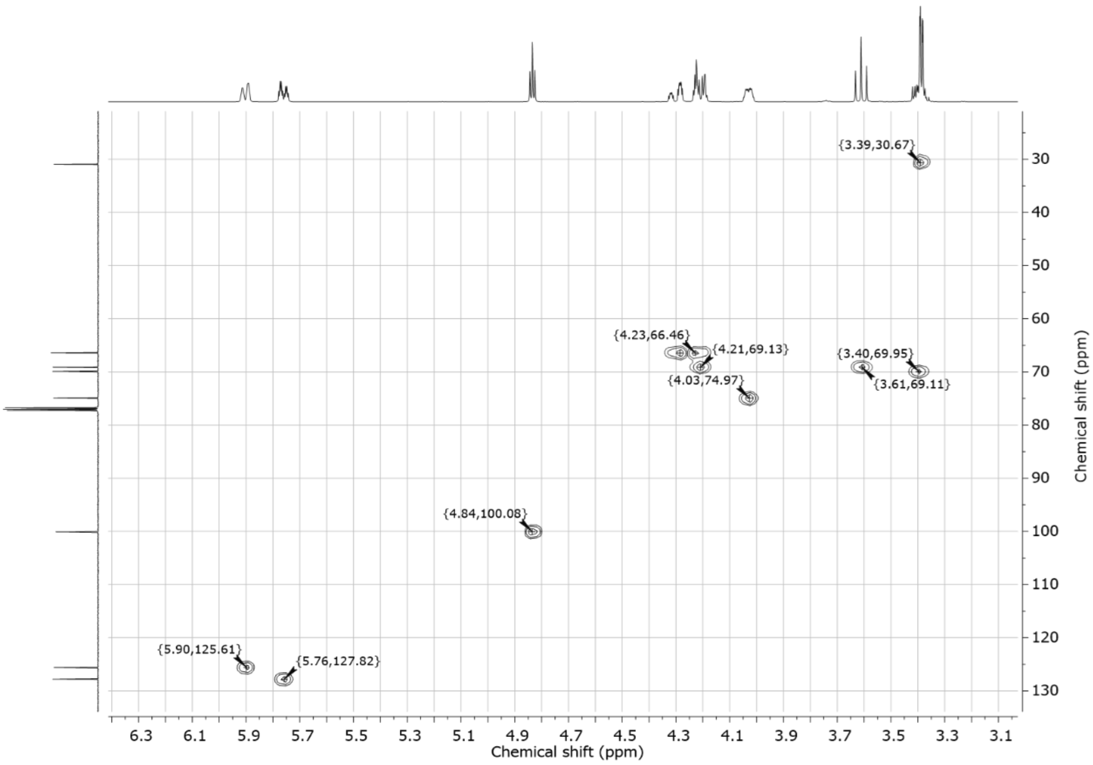

Figure S12. HSQC (^1H–^{13}C) NMR spectrum ($CDCl_3$) of (4aR,8aS)-2-(bromomethyl)-4,4a,6,8a-tetrahydro-pyrano-[3,2-d][1,3]-dioxine.

Crystal diffraction data and structure refinement for (4aR,8aS)-2-(bromomethyl)-4,4a,6,8a-tetra-hydropyrano-[3,2-d][1,3]-dioxine (CCDC Number - 2234496)

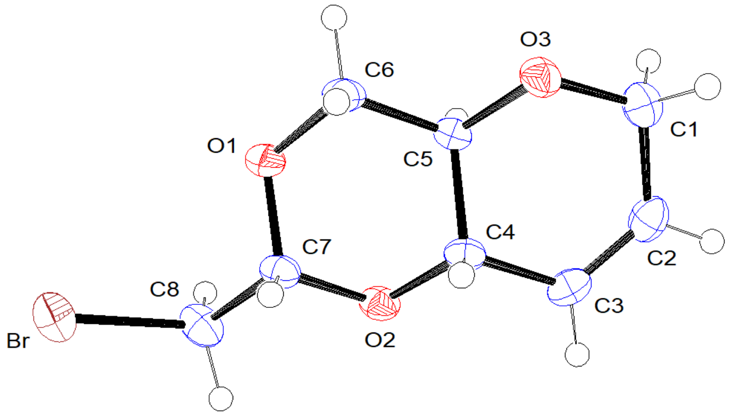

Figure S13. ORTEP drawing of the crystal structure of (4aR,8aS)-2-(bromomethyl)-4,4a,6,8a-tetrahydropyrano-[3,2-d][1,3]-dioxine with thermal ellipsoids at the 50% probability level. Selected bond lengths and dihedral angles (°): Br–C(8) 1.938 (3), O(1)–C(7) 1.414 (3), O(1)–C(6) 1.441 (3), O(2)–C(7) 1.417 (3), O(2)–C(4) 1.439 (3), C(7)–O(1)–C(6) 111.7 (2), C(5)–O(3)–C(1) 111.9 (2), C(7)–O(2)–C(4) 108.3 (2), C(7)–C(8)–Br 110.79 (19).

Identification code	e19ab1	
Empirical formula	C8 H11 Br O3	
Formula weight	235.08	
Temperature	150.01(10) K	
Wavelength	0.71073 Å	
Crystal system	Orthorhombic	
Space group	$P2_12_12_1$	
Unit cell dimensions	a = 7.4646(3) Å	α = 90°
	b = 7.7208(3) Å	β = 90°
	c = 15.7512(6) Å	γ = 90°
Volume	907.78(6) $Å^3$	
Z	4	
Density (calculated)	1.720 Mg/m^3	
Absorption coefficient	4.493 mm^{-1}	
F(000)	472	
Crystal size	0.446 x 0.269 x 0.168 mm^3	

Theta range for data collection	3.696 to 30.361°.
Index ranges	-10<=h<=10, -10<=k<=10, -20<=l<=20
Reflections collected	15936
Independent reflections	2499 [R(int) = 0.0348]
Completeness to theta = 25.242°	99.7 %
Absorption correction	Semi-empirical from equivalents
Max. and min. transmission	1.00000 and 0.64489
Refinement method	Full-matrix least-squares on F^2
Data / restraints / parameters	2499 / 0 / 109
Goodness-of-fit on F^2	1.079
Final R indices [I>2sigma(I)]	R1 = 0.0285, wR2 = 0.0493
R indices (all data)	R1 = 0.0367, wR2 = 0.0513
Absolute structure parameter	-0.005(5)
Extinction coefficient	n/a
Largest diff. peak and hole	0.300 and -0.413 e.Å^{-3}

Synthesis of (4aR,8aS)-2-methylene-4,4a,6,8a-tetrahydropyrano[3,2-d][1,3]dioxine (1)

A solution of (4aR,8aS)-2-(bromomethyl)-4,4a,6,8a-tetrahydropyrano-[3,2-d][1,3]-dioxine (2.02 g, 8.59 mmol, 1.0 equiv.) in dry THF (4 mL) was added dropwise to a 1 mol L^{-1} solution of KHMDS (3.44 g, 17.2 mmol, 2.0 equiv.) in dry THF (17.2 mL) at 0 °C under argon. The reaction mixture was then stirred at 0 °C for 1 h before being heated to 40 °C and stirred overnight, the progress of the reaction was monitored by NMR. Once complete the solvent was removed in *vacuo* and the resultant solids were washed with dry pentane (2 x 50 mL). The liquid phase was separated by centrifugation (2 x 5 min at 3500 rpm) and the solvent was then concentrated in *vacuo* and the crude product was purified *via* recrystallisation in dry pentane at –78 °C to yield a colourless oil at room temperature (0.84 g, 64 %).

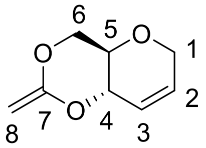

δ_H (400 MHz; benzene-d_6): 5.73–5.66 (C^2H, 1H, m), 5.14 (C^3H, 1H, dddd, J_{HH} = 10.3, 2.4, 2.3, 2.3 Hz), 4.12-4.06 (C^4H, 1H, m), 3.97 (C^6H, 1H, ddd, J_{HH} = 9.8, 5.9, 0.6 Hz), 3.85 (C^8H, 2H, 2 x d, $^2J_{HH}$ = 1.4 Hz), 3.71-3.67 (C^1H, 2H, m), 3.60 (C^6H, 1H, dd, J_{HH} = 9.8, 9.8 Hz), 3.36 (C^5H_2, 1H, ddd, J_{HH} = 9.8, 8.6, 5.9 Hz) ppm; δ_C (101 MHz; benzene-d_6): 160.5 (C^7), 128.5 (C^3), 125.0 (C^2), 71.2 (C^4), 69.9 (C^5), 68.7 (C^8), 69.3 (C^6), 66.0 (C^1) ppm. ν_{max} (cm^{-1}): 2998-2790 (CH), 1670 ($C(CH_2)$), 1087 (CO). Density (ρ): 1.35 g mL^{-1}.

NMR analysis of (4a*R*,8a*S*)-2-methylne-44a,6,8a-tetrahydropyrano[3,2-*d*][1,3]dioxine (1)

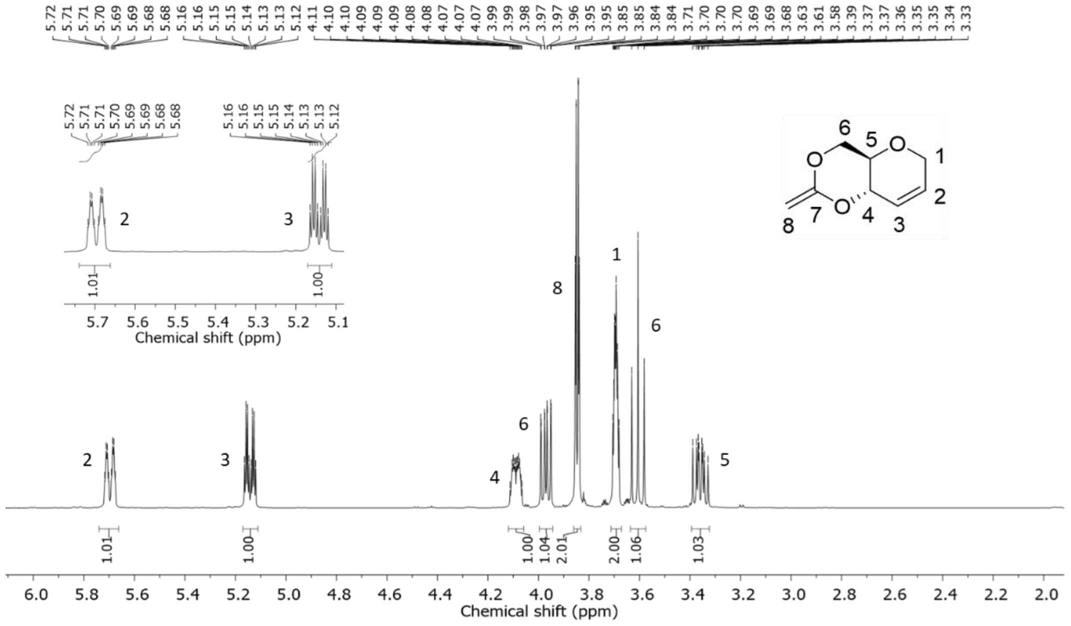

Figure S14. Annotated ^1H NMR spectrum (C_6D_6) of **1**.

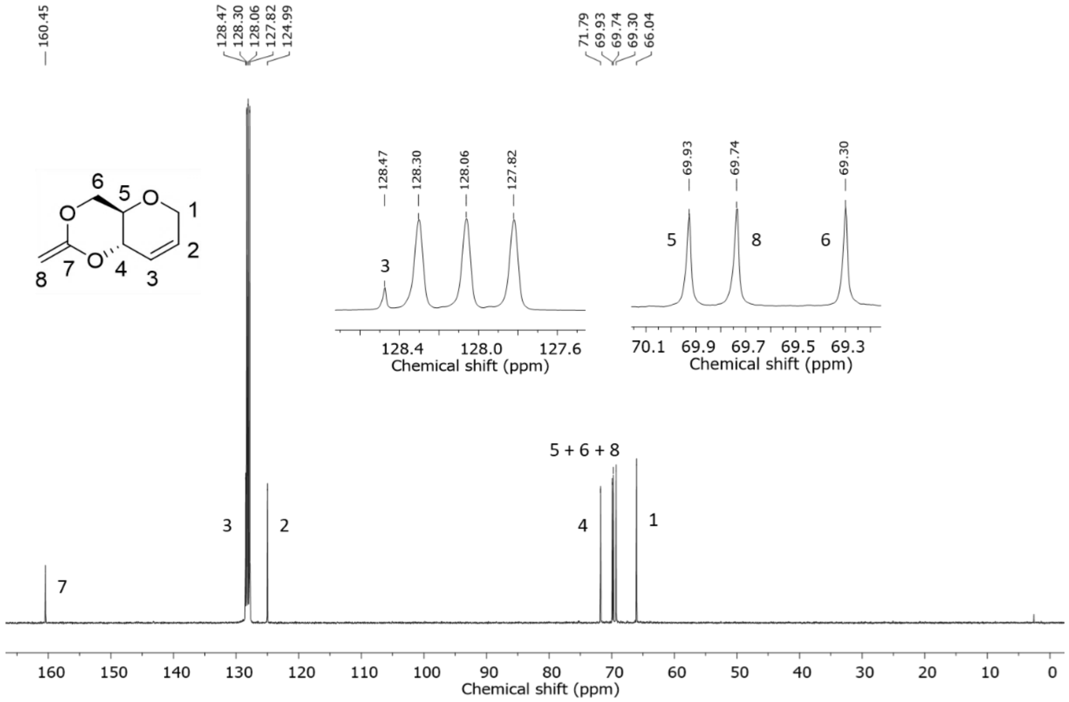

Figure S15. Annotated $^{13}C\{^{1}H\}$ NMR spectrum (C_6D_6) of **1**.

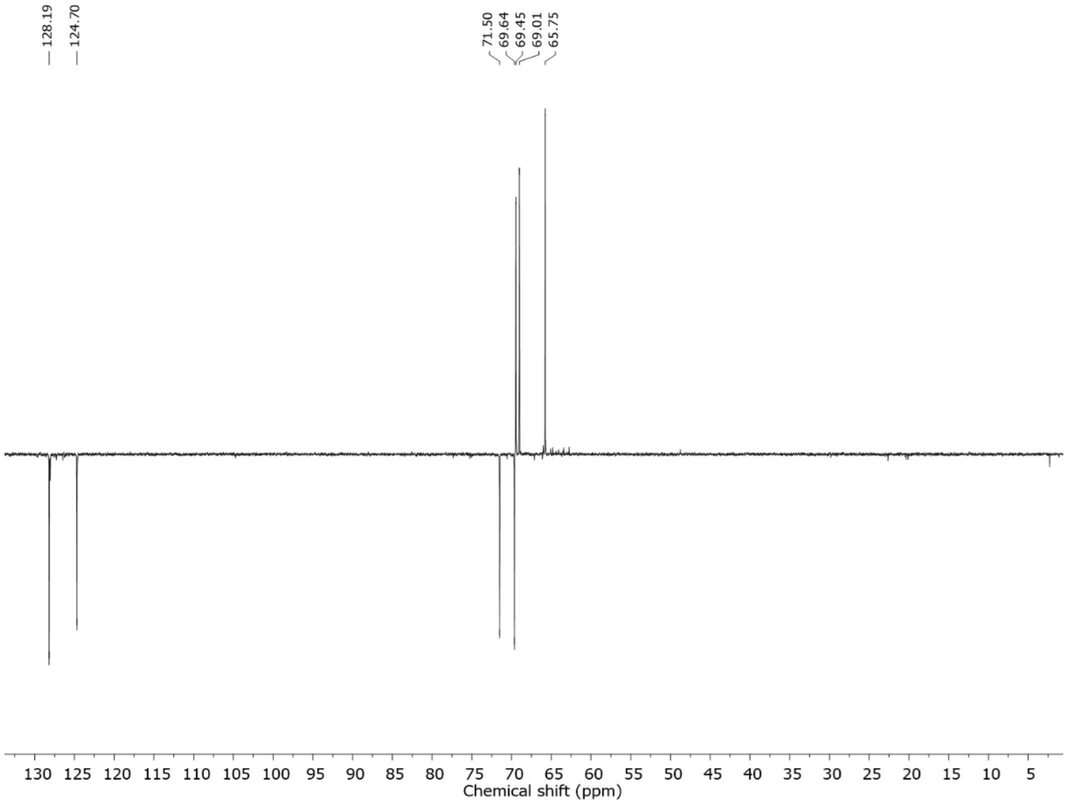

Figure S16. $^{13}C\{^{1}H\}$ DEPT135 NMR spectrum (C_6D_6) of **1**.

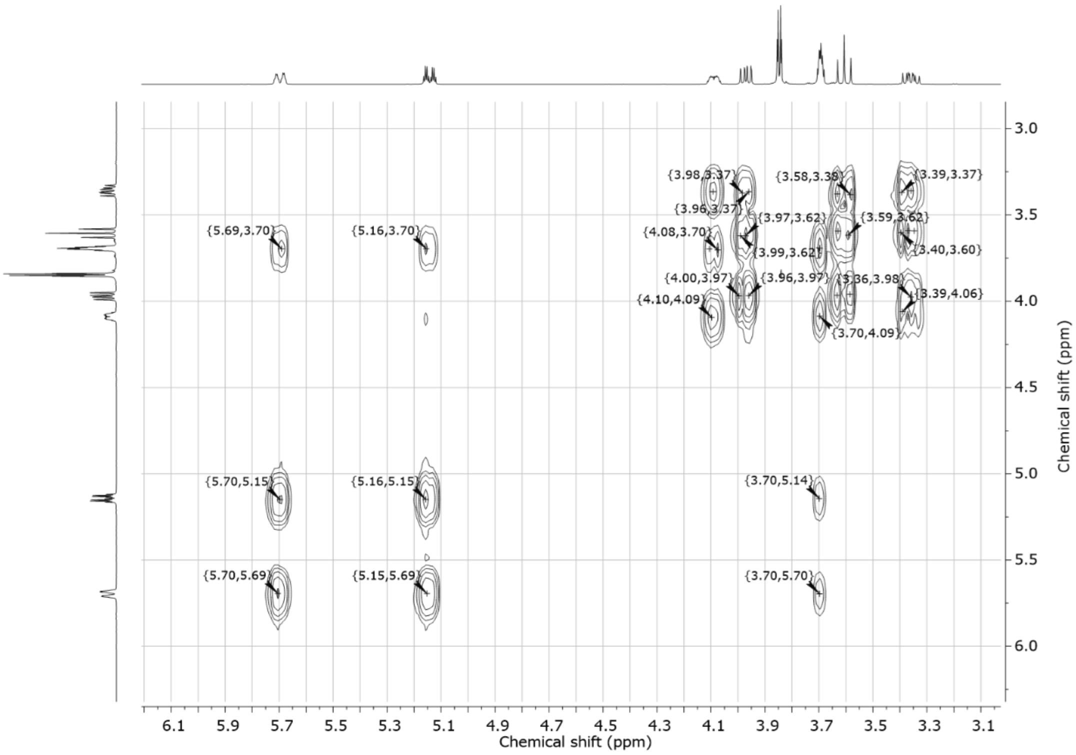

Figure S17. COSY (^1H–^1H) NMR spectrum (C_6D_6) of **1**.

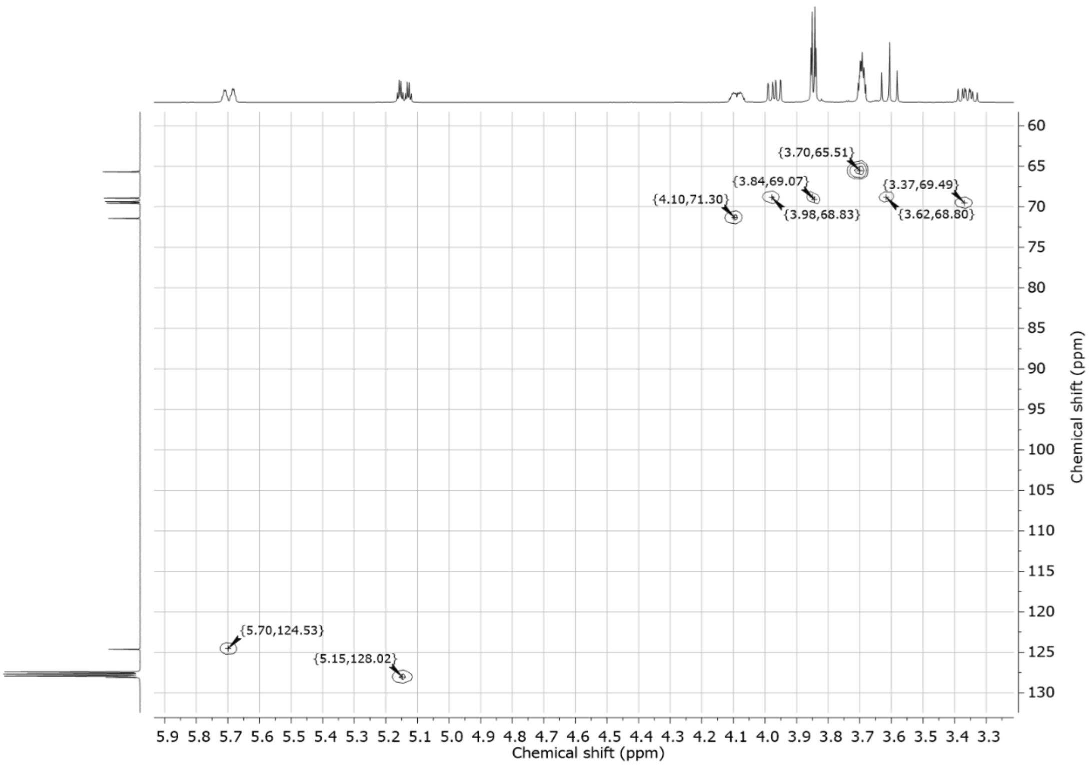

Figure S18. HSQC (^1H–^{13}C) NMR spectrum (C_6D_6) of **1**.

FT-IR analysis of (4a*R*,8a*S*)-2-methylne-44a,6,8a-tetrahydropyrano[3,2-*d*][1,3]dioxine (1)

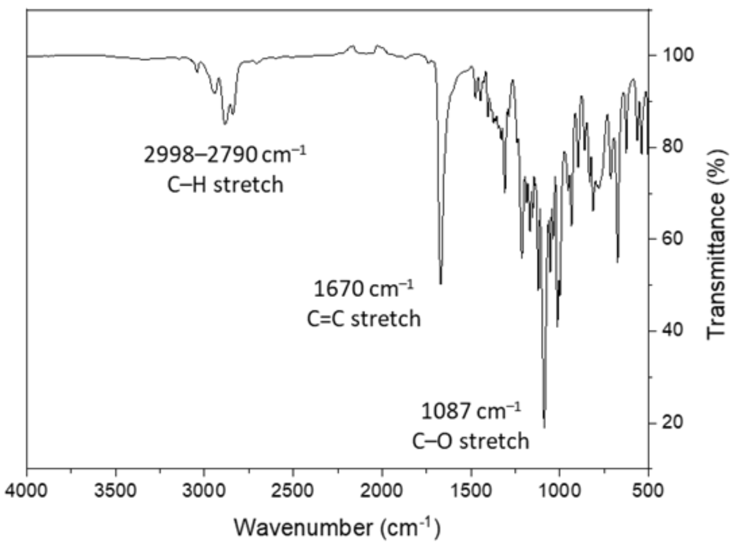

Figure S19. Labelled FT-IR spectrum of **1**.

2. Synthesis and Characterization of Cyclic Ketene Monomer, 2

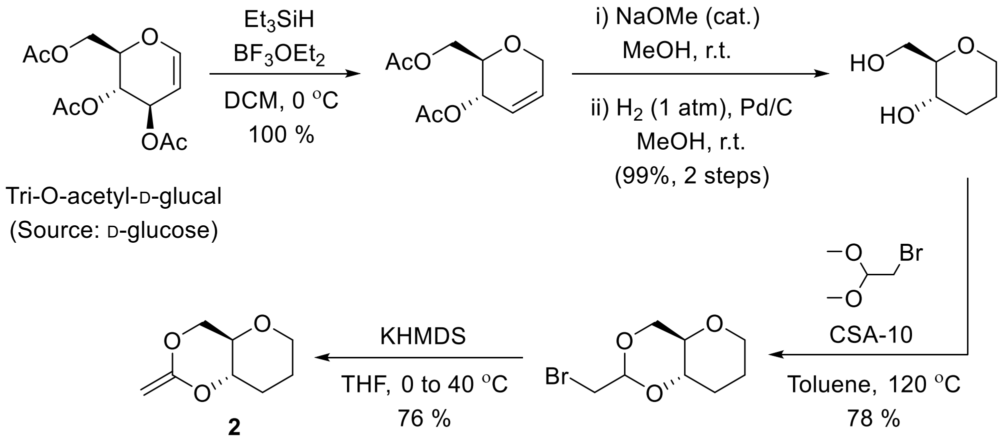

Scheme S2. Synthesis of CKA monomer **2.**

Synthesis of (2R,3S)-2-(hydroxymethyl)tetrahydro-2H-pyran-3-ol

Pd/C (10 wt% loading, 0.56 g) was added to a solution of (2R,3S)-2-(hydroxymethyl)-3,6-dihydro-2H-pyran-3-ol (5.63 g, 43.3 mmol, 1.0 equiv.) in anhydrous methanol (100 mL) under argon. The atmosphere of the flask was then exchanged, and the solution was saturated with H_2. Under a continuous feed of gas, the reaction mixture was stirred at room temperature for 20 h. Once complete, the mixture was filtered through a pad of celite and concentrated under reduced pressure to afford a colourless oil, which was used directly in the next reaction without further purification (5.61 g, 98 %). *Spectroscopic data was consistent with the literature.*[2]

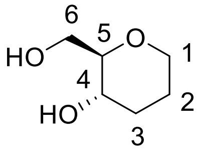

δH (400 MHz; chloroform-d): 3.93-3.86 (C^1H, 1H, m), 3.80 (C^6H, 1H, dd, J_{HH} = 11.7, 3.8 Hz), 3.75 (C^6H, 1H, dd, J_{HH} = 11.7, 4.8 Hz), 3.52 (C^4H, 1H, ddd, J_{HH} = 11.0, 9.2, 4.7 Hz), 3.38-3.31 (C^1H, 1H, m), 3.10 (C^5H, 1H, ddd, J_{HH} = 8.9, 4.3, 4.3 Hz), 2.12-2.05 (C^3H, 1H, m), 1.73-1.57 (C^2H, 2H, m), 1.49-1.34 (C^3H, 1H, m) ppm; δ_C (101 MHz; chloroform-d): 82.0 (C^5), 67.8 (C^1), 67.3 (C^4), 63.2 (C^6), 32.5 (C^3), 25.5 (C^2) ppm.

NMR analysis of (2R,3S)-2-(hydroxymethyl)tetrahydro-2H-pyran-3-ol

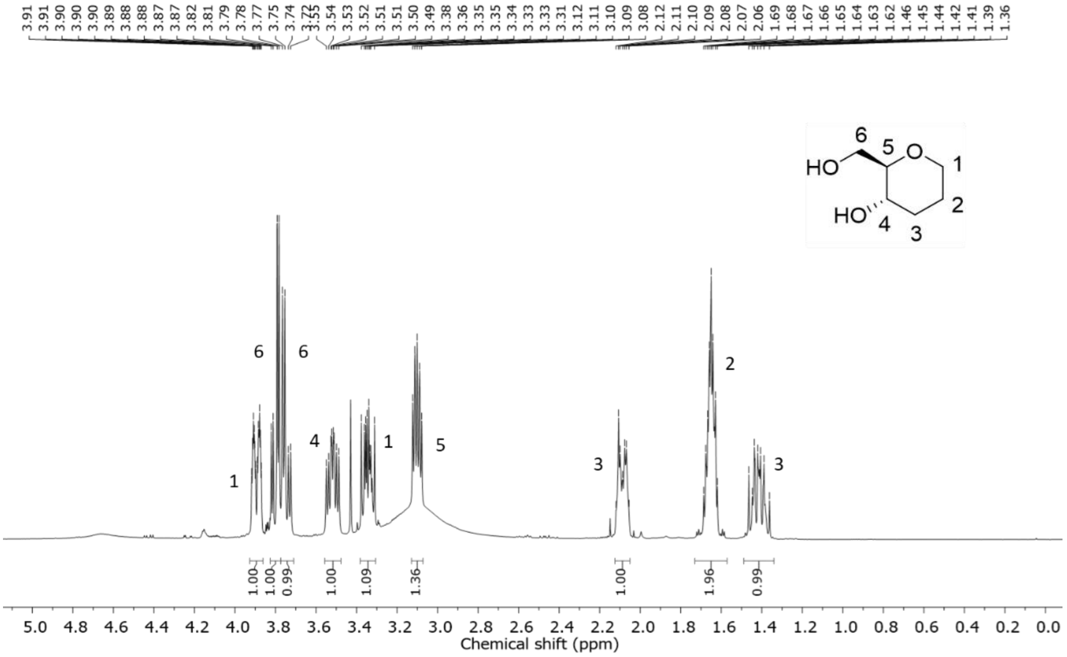

Figure S20. Annotated ^{1}H spectrum ($CDCl_3$) of (2R,3S)-2-(hydroxymethyl)tetrahydro-2H-pyran-3-ol.

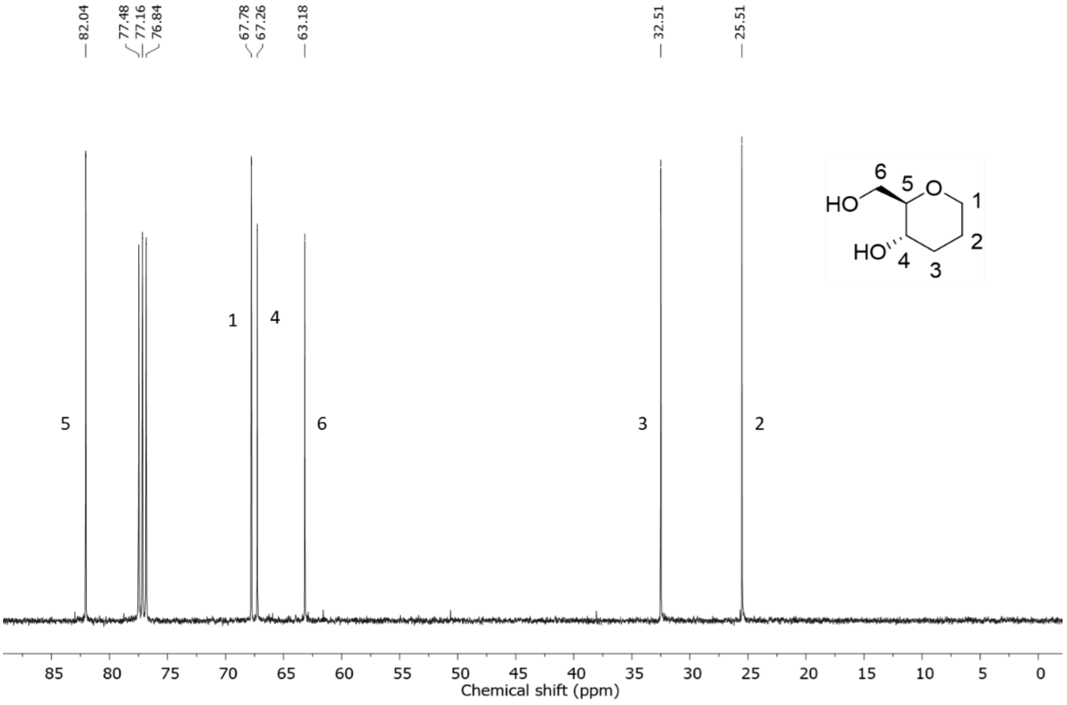

Figure S21. Annotated $^{13}C\{^{1}H\}$ NMR spectrum ($CDCl_3$) of (2R,3S)-2-(hydroxymethyl)tetrahydro-2H-pyran-3-ol.

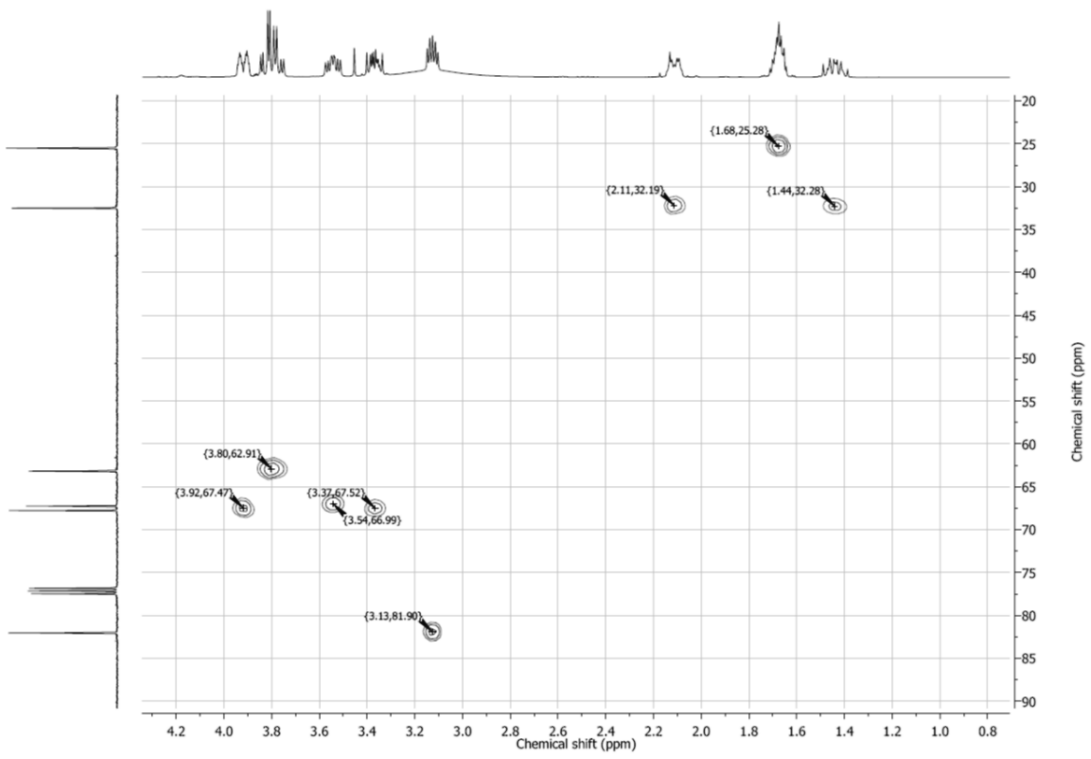

Figure S22. HSQC (^1H–^{13}C) NMR spectrum ($CDCl_3$) of (2R,3S)-2-(hydroxymethyl)tetrahydro-2H-pyran-3-ol.

Synthesis of (4aR,8aS)-2-(bromomethyl)hexahydropyrano[3,2-d][1,3]dioxine

A solution of 2-bromo-1,1-dimethoxyethane (5.53 mL, 46.8 mmol, 1.2 equiv.) and (2R,3S)-2-(hydroxymethyl)tetrahydro-2H-pyran-3-ol (5.15 g, 38.9 mmol, 1.0 equiv.) in toluene (10 mL) was treated with (1*S*)-(+)-10-Camphorsulfonic acid (0.91 g, 3.89 mmol, 0.1 equiv.) and heated to 120 °C. The reaction was stirred for 6 h, and a black solution was formed. Once complete, the solvent was removed in *vacuo* to yield a black solid that was subsequently purified *via* column chromatography on SiO_2 using a hexane:EtOAc (4:1) mobile phase, fractions containing the product were combined and concentrated in *vacuo* to a yield pale yellow solid once cooled (7.21 g, 78 %).

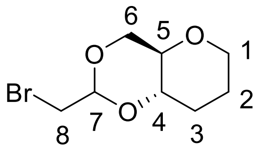

δ_H (500 MHz; chloroform-d): 4.76 (C^7H, 1H, t, $^3J_{HH}$ = 4.5 Hz), 4.10 (C^6H, 1H, dd, J_{HH} = 10.5, 4.9 Hz), 3.91-3.85 (C^1H, 1H, m), 3.48 (C^6H, 1H, dd, J_{HH} = 10.3, 10.3 Hz), 3.42 (C^1H, 1H, ddd, J_{HH} = 11.4, 11.4, 3.9 Hz), 3.38-3.30 (C^4H, C^8H, 3H, m), 3.18 (C^5H, 1H, ddd, J_{HH} = 10.2, 8.9, 4.9 Hz), 2.07-2.01 (C^3H, 1H, m), 1.81-1.69 (C^2H, 2H, m), 1.61-1.51 (C^3H, 1H, m) ppm; δ_C (126 MHz; chloroform-d): 100.3 (C^7), 78.2 (C^4), 73.7 (C^5), 69.1 (C^6), 68.1 (C^1), 31.4 (C^8), 28.6 (C^3), 25.5 (C^2) ppm.

NMR analysis of (4aR,8aS)-2-(bromoethyl)hexahydropyrano[3,2-d][1,3]dioxine

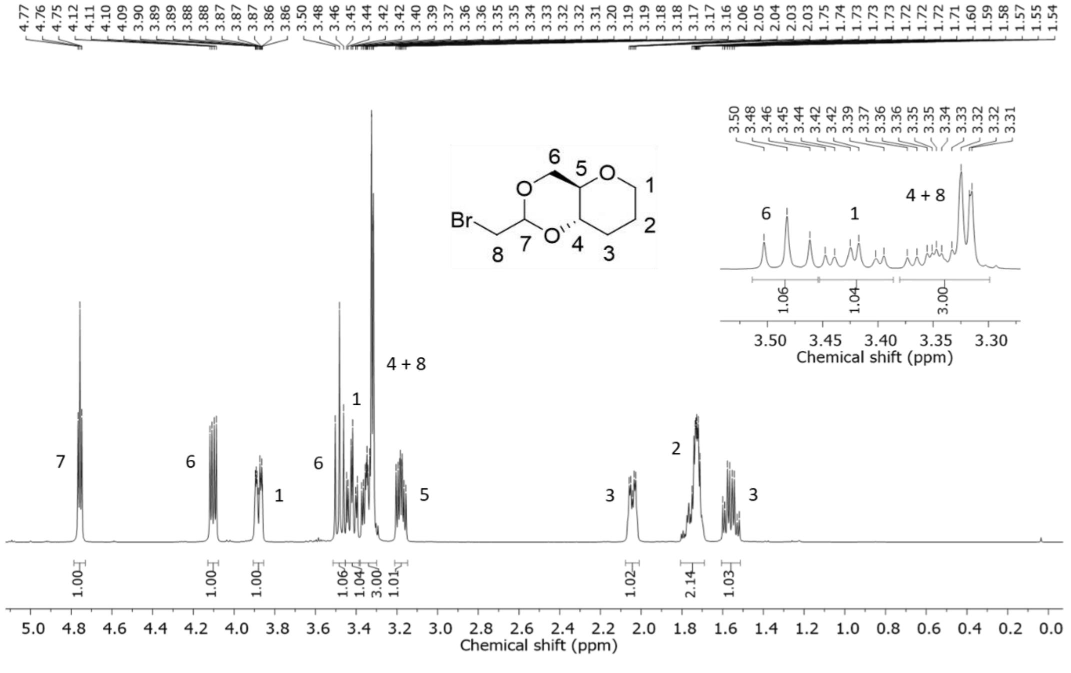

Figure S23. Annotated ^{1}H NMR spectrum ($CDCl_3$) of (4aR,8aS)-2-(bromomethyl)hexahydropyrano[3,2-d]-[1,3]dioxine.

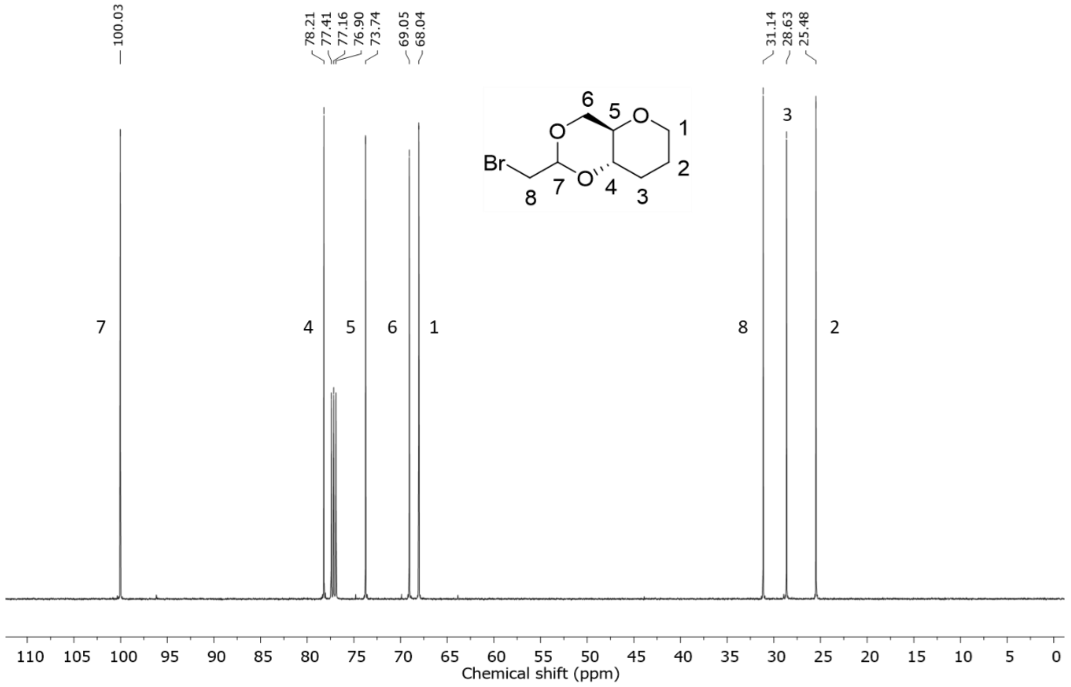

Figure S24. Annotated $^{13}C\{^{1}H\}$ NMR spectrum ($CDCl_3$) of (4aR,8aS)-2-(bromomethyl)hexahydropyrano [3, 2-d][1,3]-dioxine.

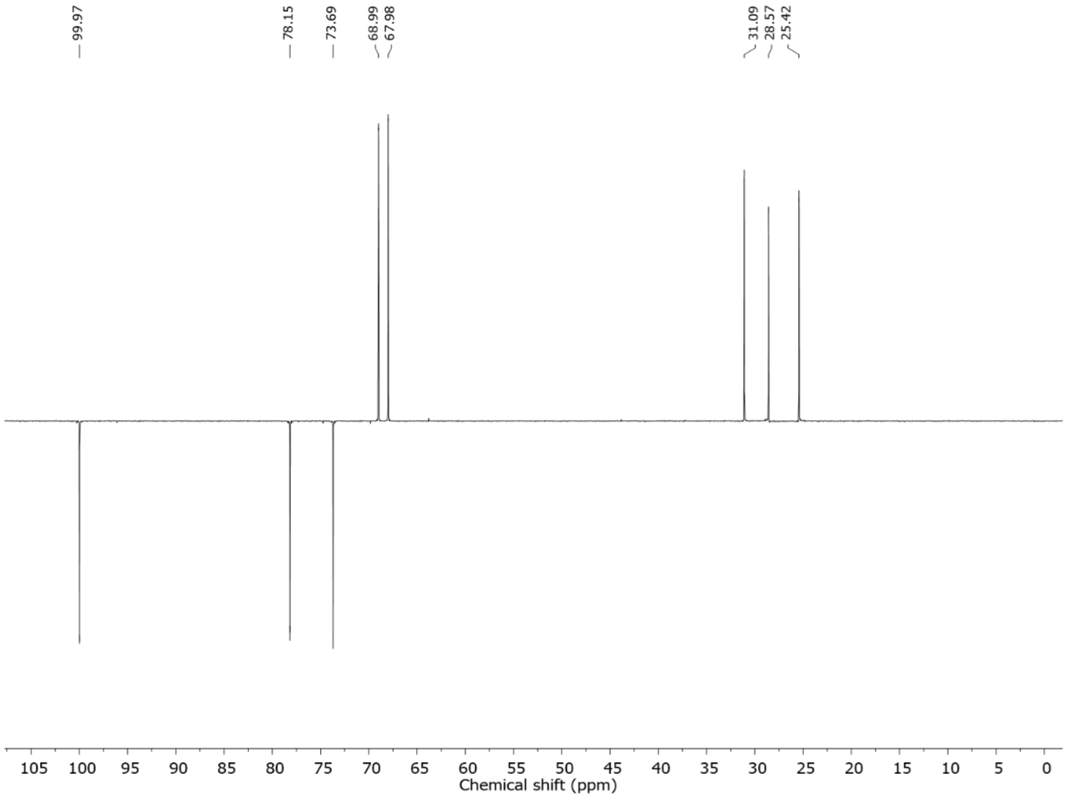

Figure S25. $^{13}C\{^{1}H\}$ DEPT135 NMR spectrum ($CDCl_3$) of (4aR,8aS)-2-(bromomethyl)hexahydropyrano [3, 2-d][1,3]-dioxine.

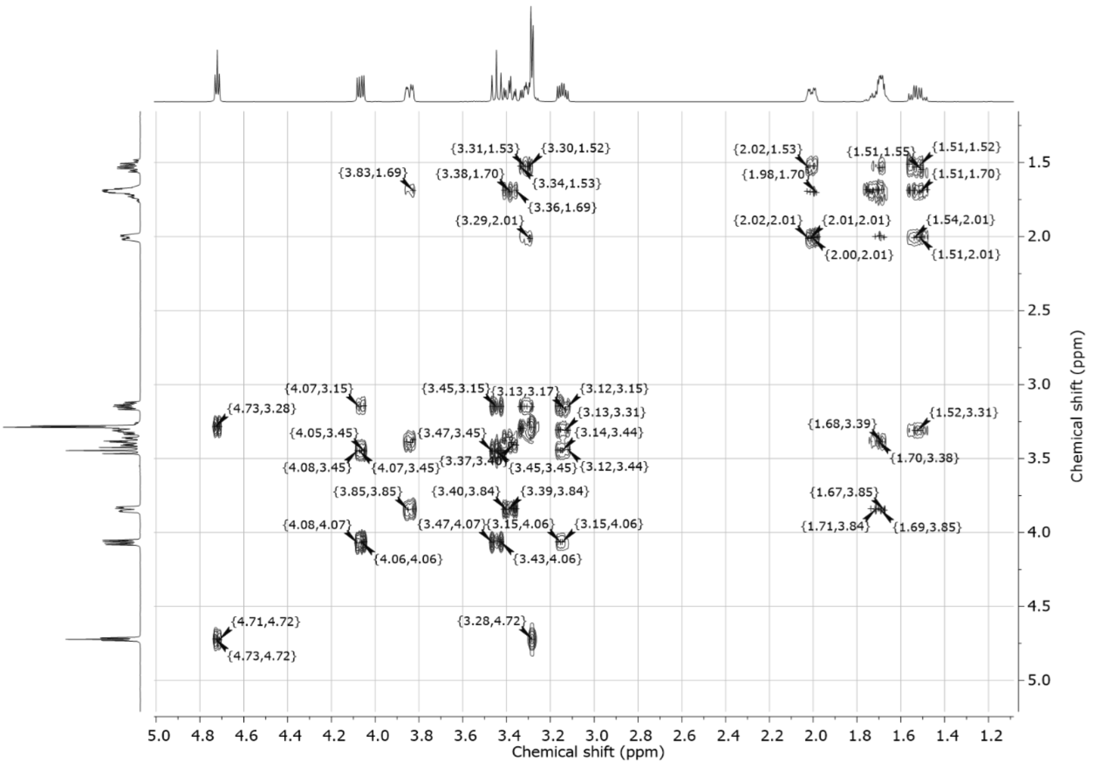

Figure S26. COSY (^1H–^1H) NMR spectrum ($CDCl_3$) of (4aR,8aS)-2-(bromomethyl)hexahydropyrano[3,2-d][1,3]dioxine.

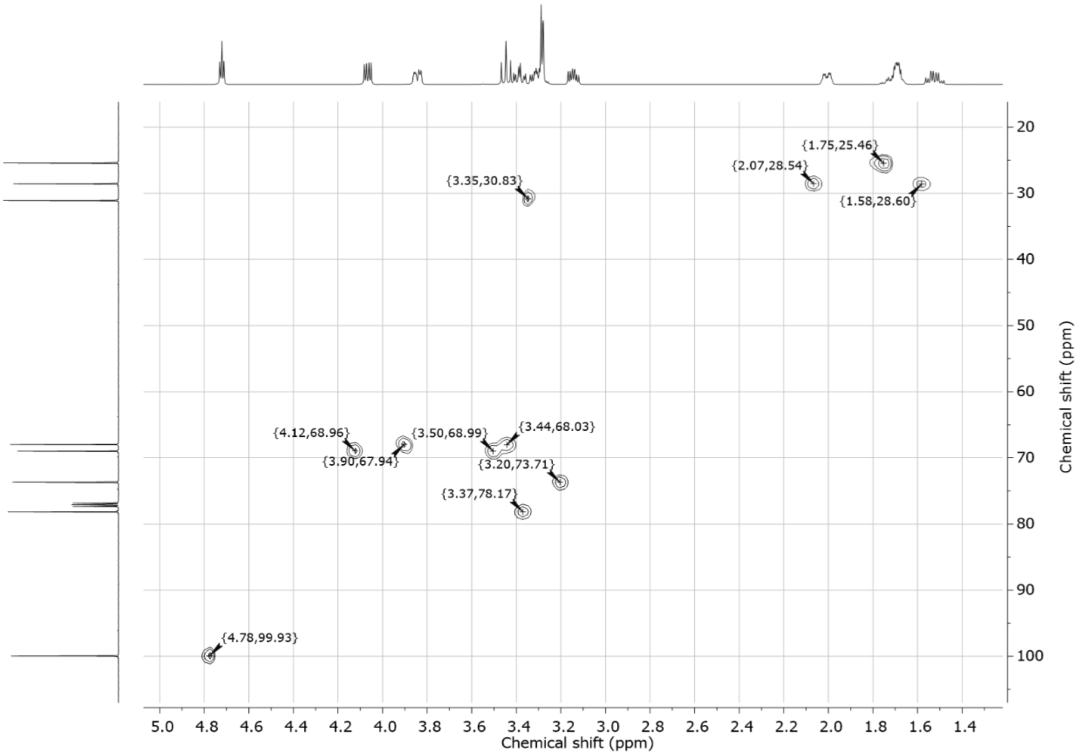

Figure S27. HSQC (^1H–^{13}C) NMR spectrum ($CDCl_3$) of (4aR,8aS)-2-(bromomethyl)hexahydropyrano[3,2-d][1,3]-dioxine.

Synthesis of (4aR,8aS)-2-methylene-4,4a,6,8a-tetrahydropyrano[3,2-d][1,3]dioxine (2)

A solution of (4aR,8aS)-2-(bromomethyl)hexahydropyrano-[3,2-d][1,3]-dioxine (1.00 g, 4.2 mmol, 1.0 equiv.) in dry THF (2.1 mL) was added dropwise to a 1 mol L^{-1} solution of KHMDS (1.68 g, 8.44 mmol, 2.0 equiv.) in dry THF (8.4 mL) at 0 °C under argon. The reaction mixture was then stirred at 0 °C for 1 h before being heated to 40 °C and stirred overnight, the progress of the reaction was monitored by NMR. Once complete the solvent was removed in *vacuo* and the resultant solids were washed with dry pentane (1 x 50 mL). The liquid phase was separated by centrifugation (2 x 5 min at 3500 rpm) and the solvent was then concentrated in *vacuo* and the crude product was purified *via* recrystallisation in dry pentane at –78 °C to yield a off-white solid at room temperature (0.51 g, 76 %).

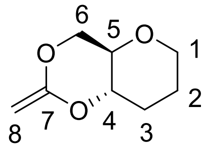

δH (500 MHz; benzene-d6): 3.97 (C^6H, 1H, dd, J_{HH} = 10.1, 6.0 Hz), 3.85 (C^8H, 2H, s), 3.60 (C^6H, 1H, dd, J_{HH} = 10.1, 9.2 Hz), 3.48-3.42 (C^1H, 1H, m), 3.35 (C^4H, 1H, ddd, J_{HH} = 10.9, 9.2, 4.4 Hz), 3.09 (C^5H, 1H, ddd, J_{HH} = 9.3, 9.2, 6.0 Hz), 2.88-2.80 (C^1H, 1H, m), 1.77-1.68 (C^3H, 1H, m), 1.25-1.13 (C^2H, C^3H, 2H, m), 1.05-0.96 (C^2H, 1H, m) ppm; δ_C (126 MHz; benzene-d6): 160.7 (C^7), 75.2 (C^4), 73.6 (C^5), 69.2 (C^6), 69.2 (C^8), 67.5 (C^1), 28.6 (C^3), 25.1 (C^2) ppm.

NMR analysis of (4aR,8aS)-2-methylene-4,4a,6,8a-tetrahydropyrano[3,2-d][1,3]dioxine (2)

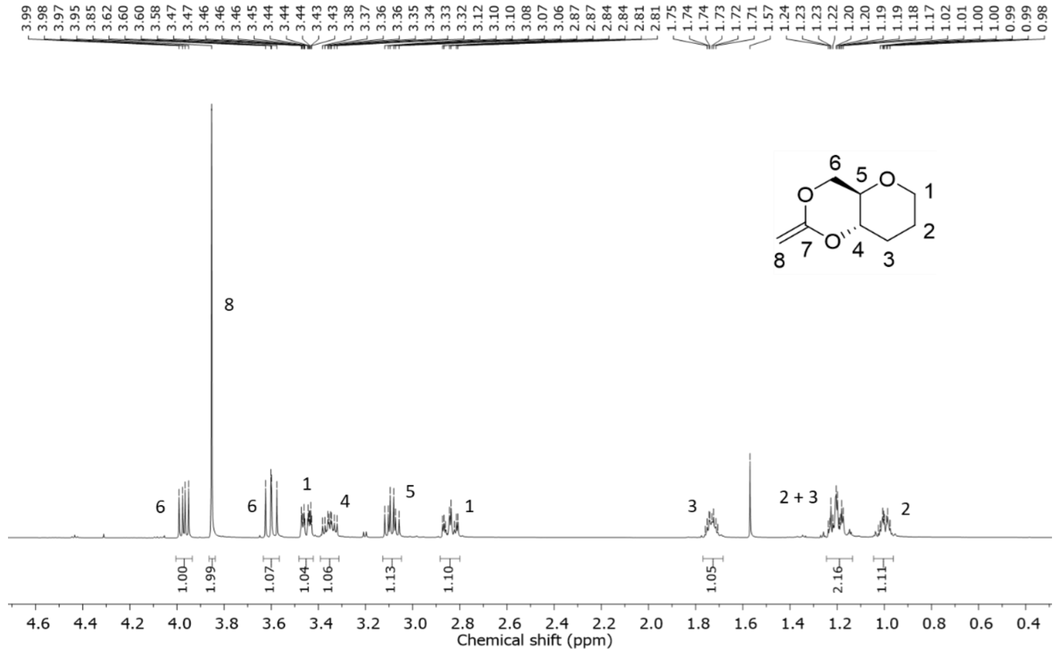

Figure S28. Annotated ^1H NMR spectrum (C_6D_6) of **2**.

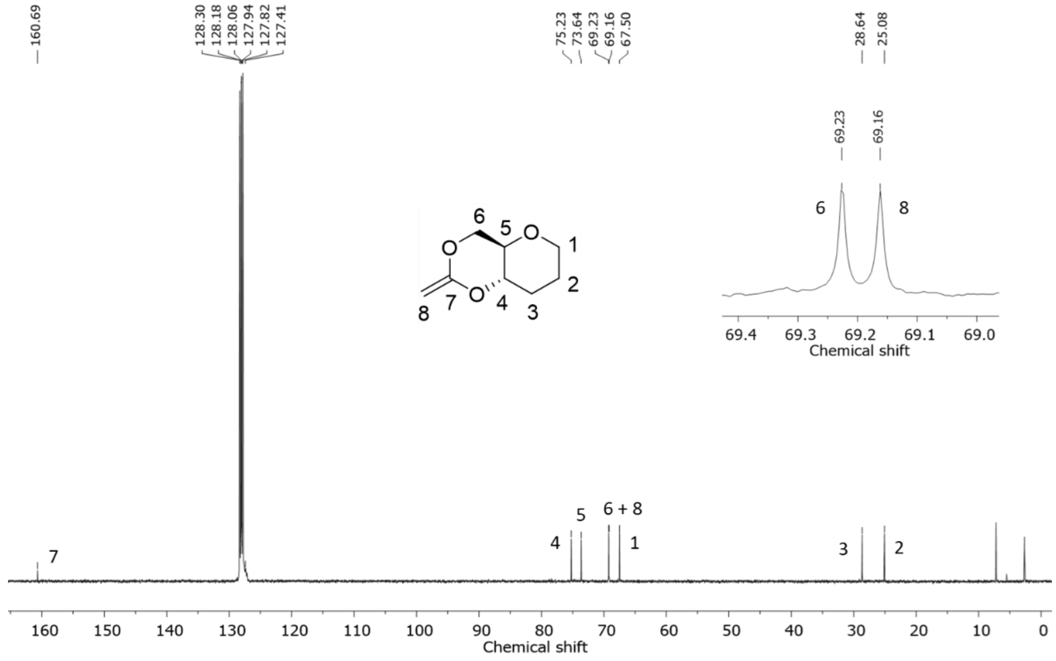

Figure S29. Annotated $^{13}C\{^{1}H\}$ NMR spectrum (C_6D_6) of **2**.

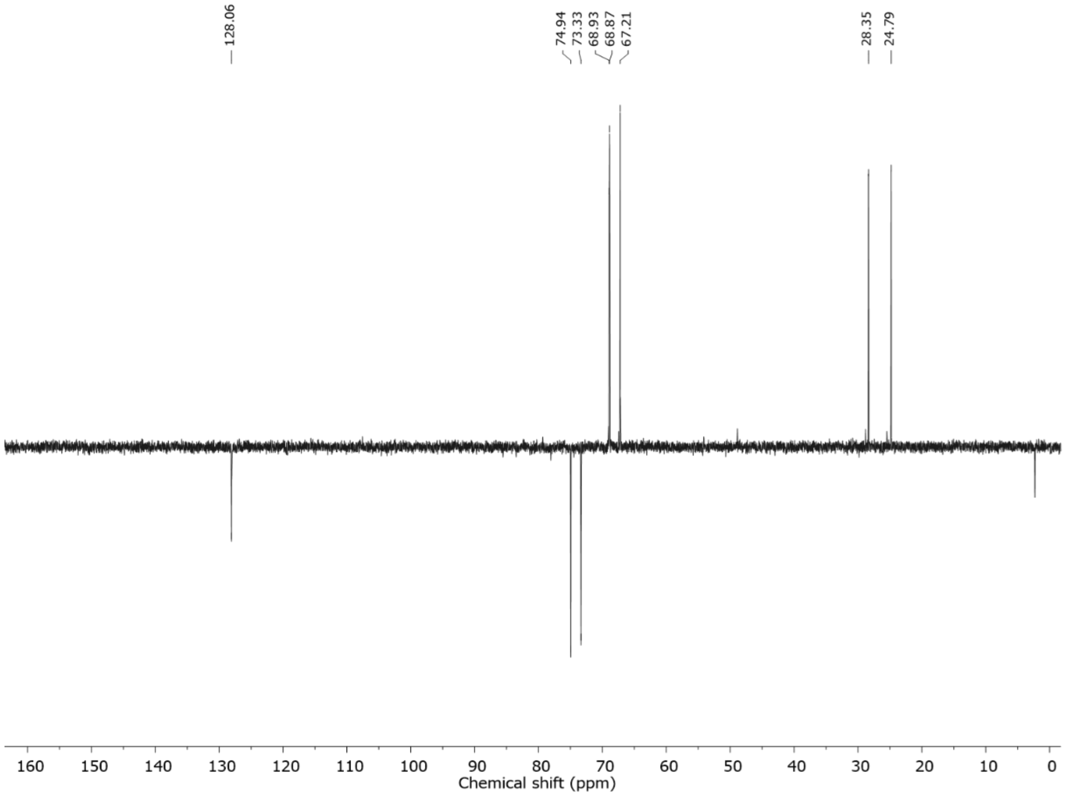

Figure S30. $^{13}C\{^{1}H\}$ DEPT135 NMR spectrum (C_6D_6) of **2**. Residual benzene signal at δ_C = 128.1 ppm.

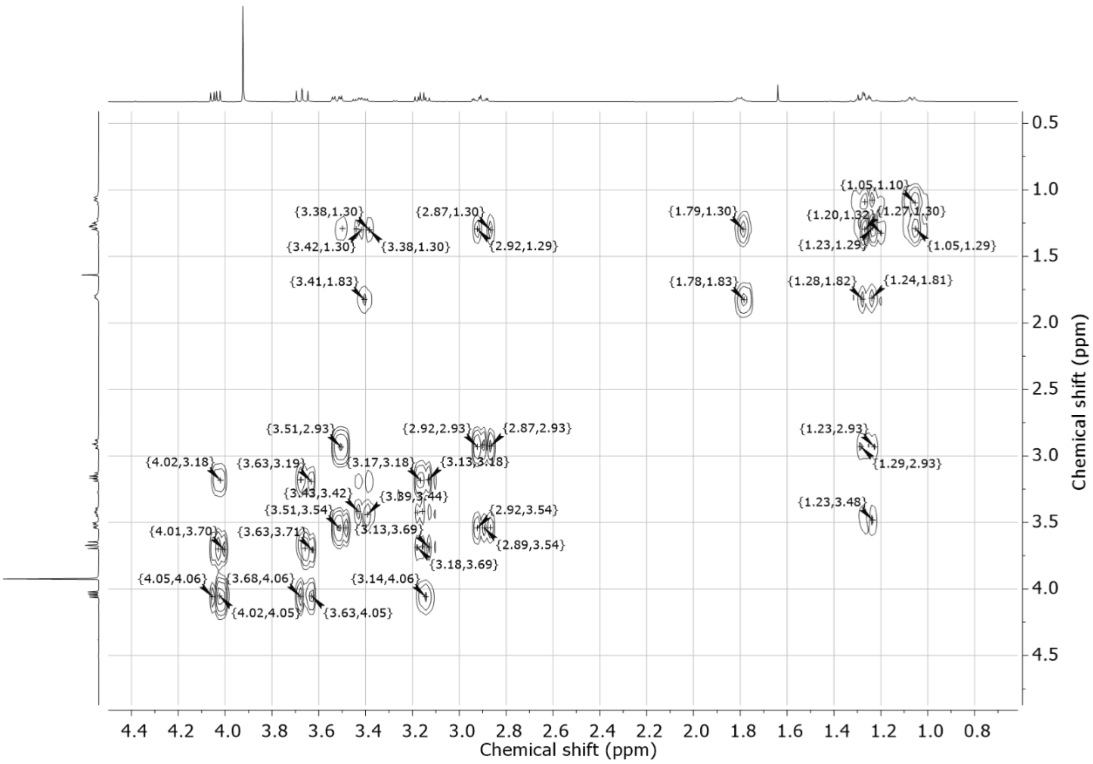

Figure S31. COSY (^1H–^1H) NMR spectrum (C_6D_6) of **2.**

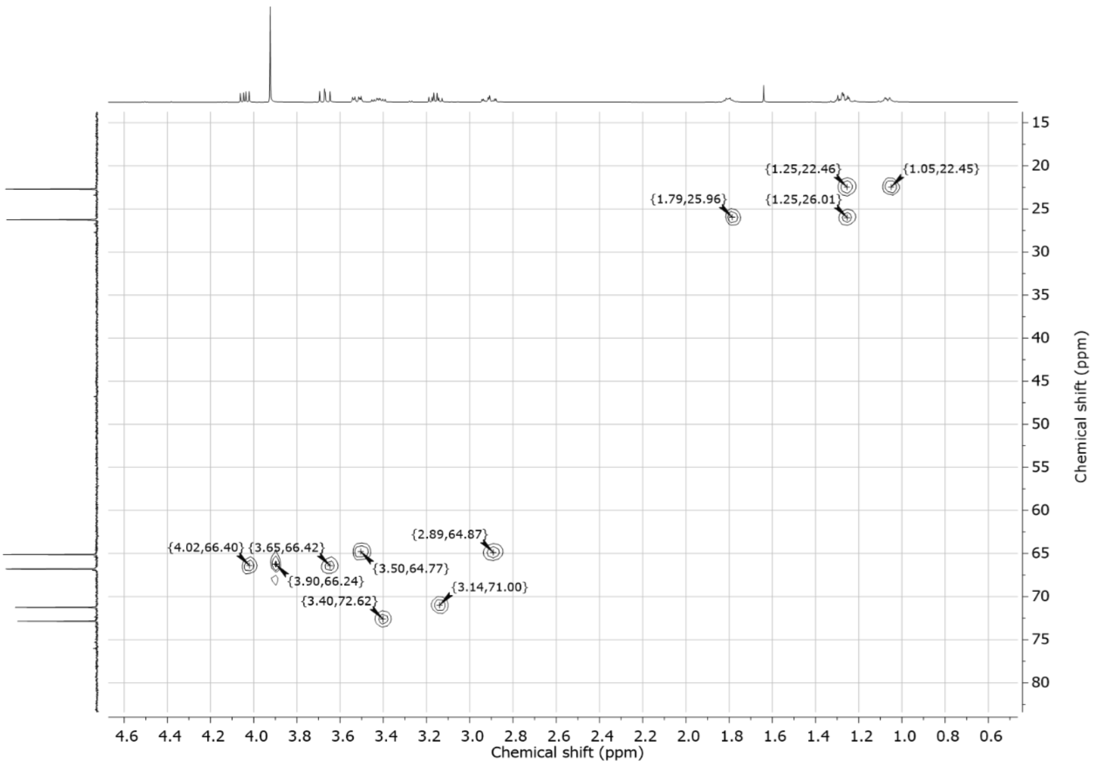

Figure S32. HSQC (^1H–^{13}C) NMR spectrum (C_6D_6) of **2.**

3. Free radical polymerization of 1 (solvent-free conditions)

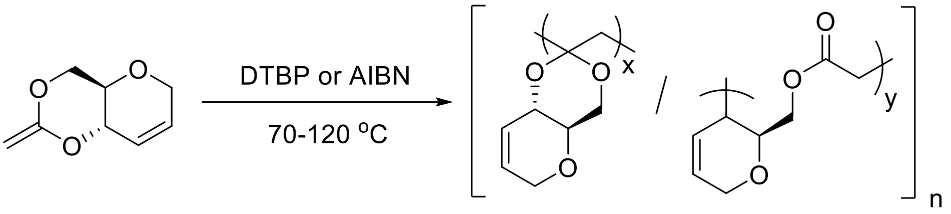

Under an argon atmosphere **1** (0.20 g, 1.30 mmol, 50 equiv.) was added into a silane washed Schlenk tube followed by three freeze-cooling-thaw cycles prior to the addition of the chosen radical initiator (1.0 equiv.). The reaction vessel was maintained under an inert atmosphere, placed into an oil bath at 70–120 °C and stirred for 20 h. The reaction was quenched by rapid cooling and the crude solid was dissolved in $CHCl_3$ and filtered before precipitation from excess hexane. The product was isolated by centrifugation (3500 rpm, 2 x 5 minutes), washed twice with hexane, and dried under vacuum. The polymer was isolated as a pale-brown solid (0.11 g, 57% yield).

δ_H (500 MHz; chloroform-d): 5.98-5.57 (C^2H, C^3H, 2H, m), 4.59-4.02 (C^1H, C^4H, 3H, m), 3.92-2.24 (C^5H, C^6H, 3H, m), 2.85-2.28 ($C^{10}H$, 1.5H, m), 2.17-2.02 (C^8H, 0.5H, m) ppm; δ_C (126 MHz; chloroform-d): 171.9 (C^7), 170.9 (C^7), 129.5-124.7 (C^2, C^3), 112.8 (C^9), 77.0 (C^5), 70.9 (C^5), 67.8-62.4 (C^1, C^4, C^6), 40.0 (C^{10}), 21.2 (C^8), 21.0 (C^8) ppm. ν_{max} (cm^{-1}): 2998-2920 (CH), 1733 (C(O)O), 1063 (CO).

Table S1. Radical polymerization of **1** in solvent-free conditions.[a]

Entry	[I]	[**1**]$_0$:[I]$_0$[b]	Temp. (°C)	Conv.[c] (%)	$M_{n,SEC}$[d] [$Đ_M$] (kg mol^{-1})
1	AIBN	50:1	70	90	2.2 [2.58]
2	AIBN	50:1	120	19	0.6 [2.38]
3	DTBP	50:1	120	99	3.1 [2.17]

[a]Reactions were carried out in solvent-free conditions at 70 or 120 °C for 20 hours, under an argon atmosphere (**1** = monomer; [**1**] = 8.8 mol L^{-1}); [b]I = initiator; [c]Monomer conversion to polymer, calculated based on the relative integration of the methylene proton signal of **1** (δ_H = 3.85 ppm, d, 2H) and the resultant alkyl proton signal(s) of poly(**1**) (δ_H = 2.85–2.28 and/or 2.17–2.02, m, 2H), in the ^1H NMR spectrum; [d]Number-average molar mass and Dispersity ($M_{n,SEC}$, $Đ_M$), calculated by SEC relative to polystyrene standards in THF eluent.

NMR analysis of the poly(1) (solvent-free conditions)

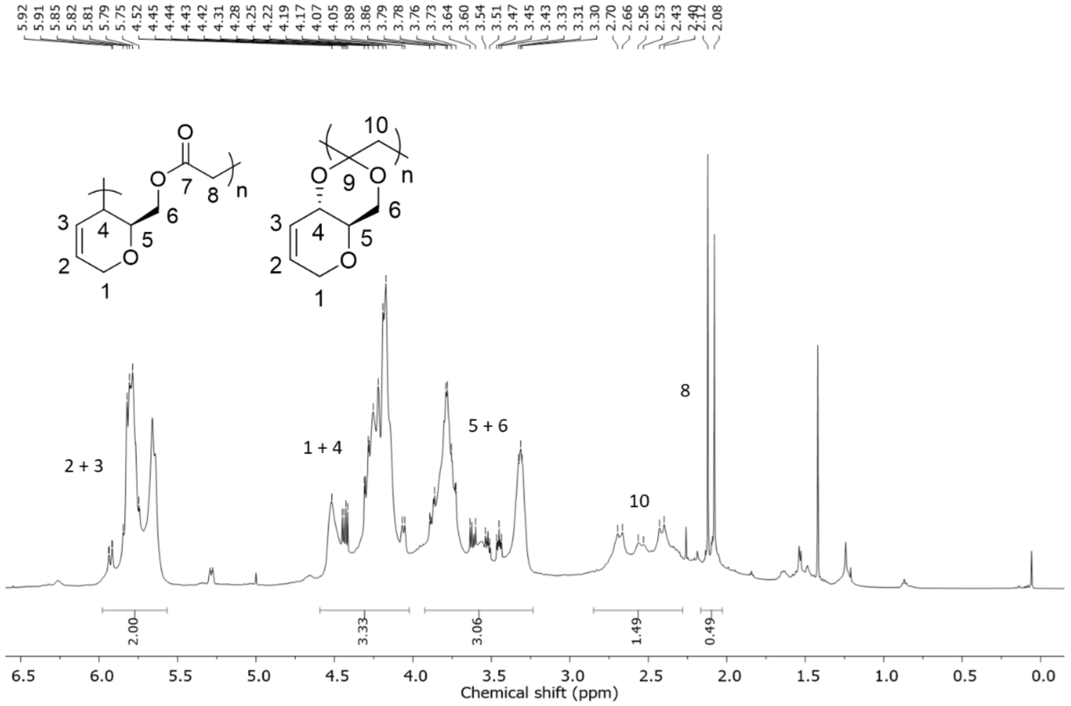

Figure S33. Annotated ^{1}H NMR spectrum ($CDCl_3$) of poly(**1**) (M_n 3,100 g mol^{-1} ($Đ_M$ 2.17), F_E/F_A = 23/77).

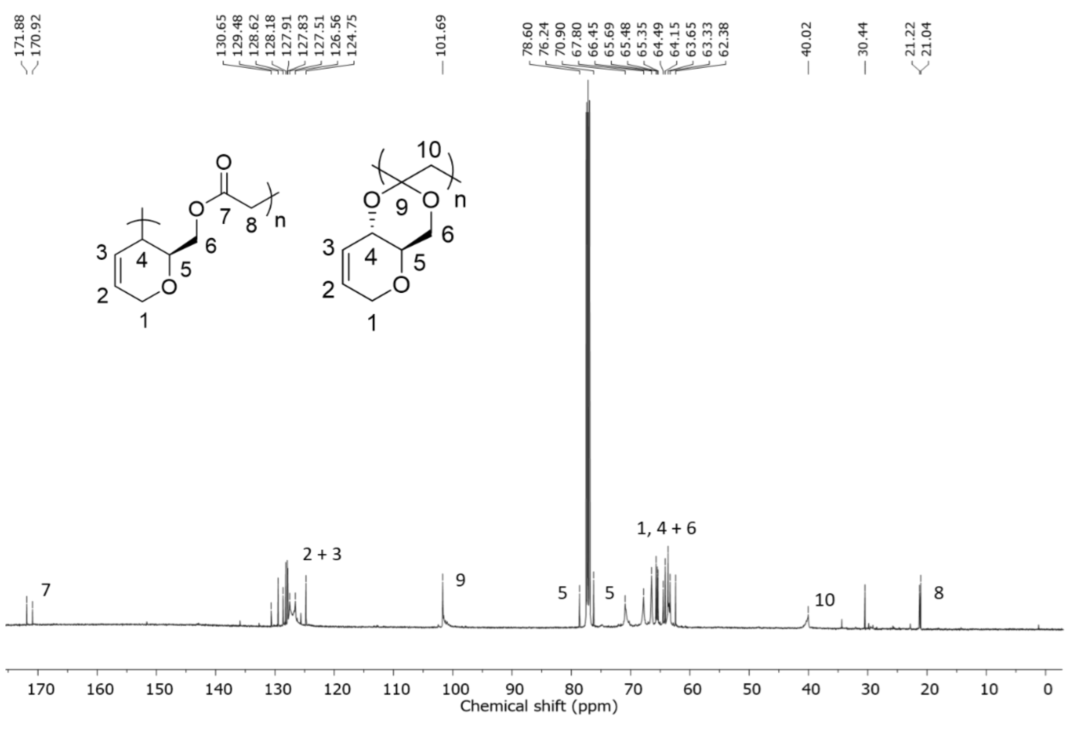

Figure S34. Annotated ^{13}C{^{1}H} NMR spectrum ($CDCl_3$) of poly(**1**) (M_n 3,100 g mol^{-1} ($Đ_M$ 2.17), F_E/F_A = 23/77).

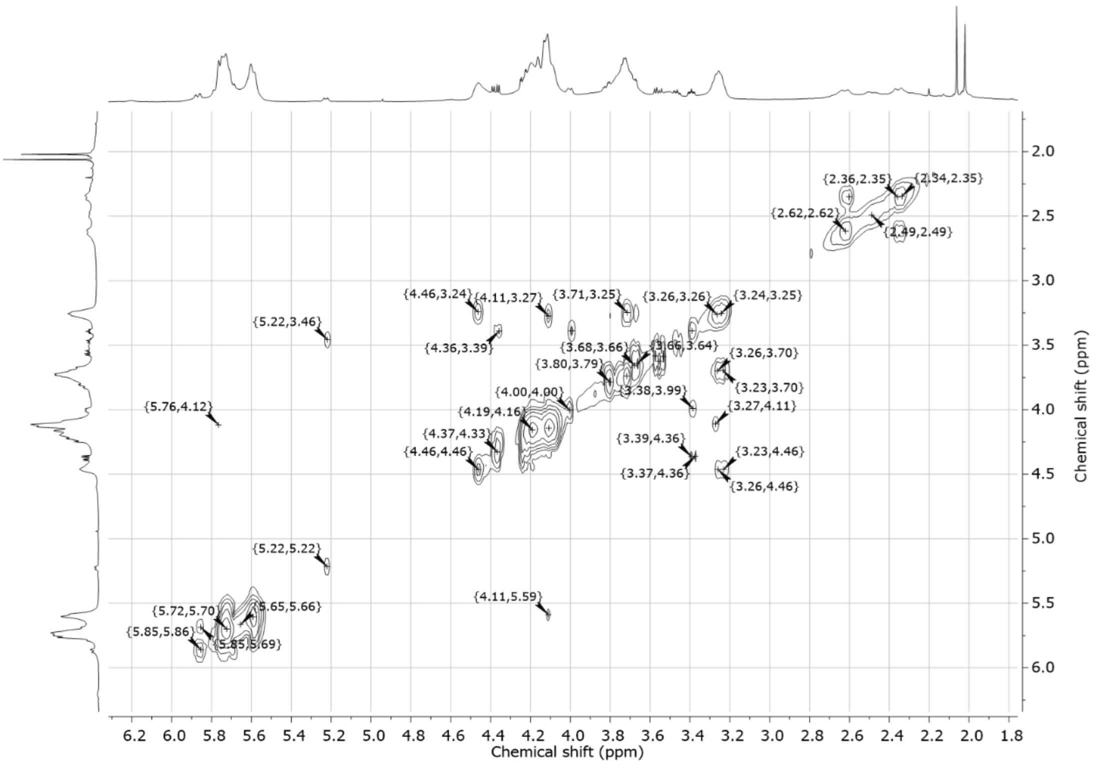

Figure S35. COSY (^{1}H–^{1}H) NMR spectrum ($CDCl_3$) of poly(**1**) (M_n 3,100 g mol^{-1} ($Đ_M$ 2.17), F_E/F_A = 23/77).

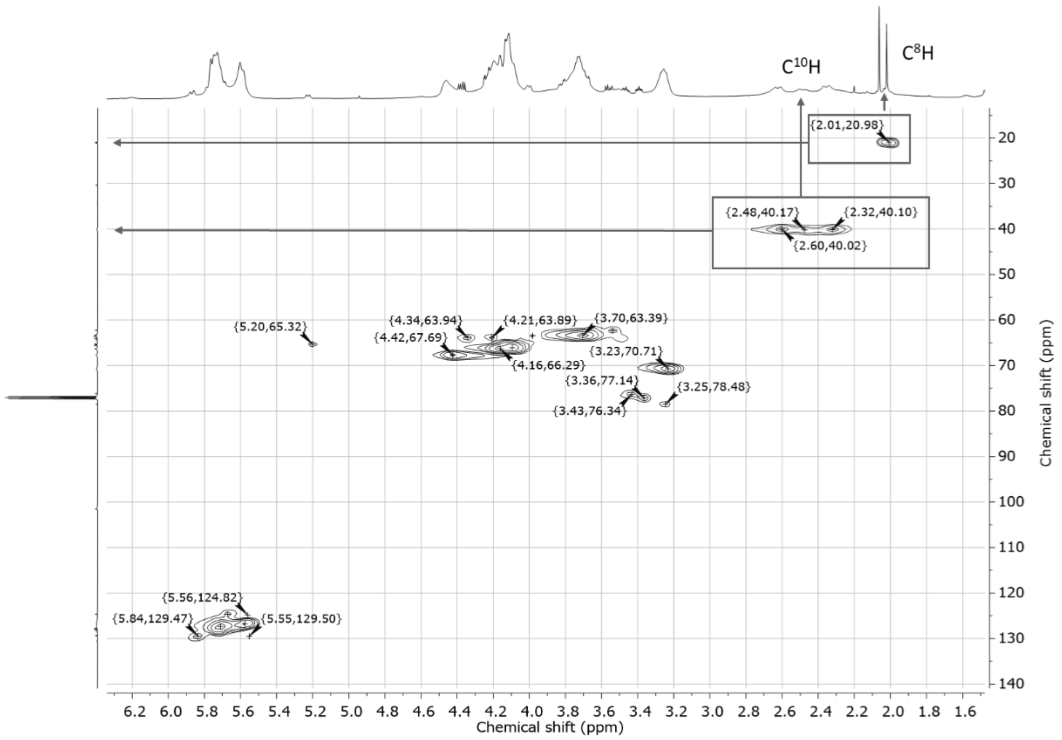

Figure S36. HSQC (^{1}H–^{13}C) NMR spectrum ($CDCl_3$) of poly(**1**) (M_n 3,100 g mol^{-1} ($_M$ 2.17), F_E/F_A = 23/77).

FT-IR analysis of poly(1) (solvent-free conditions)

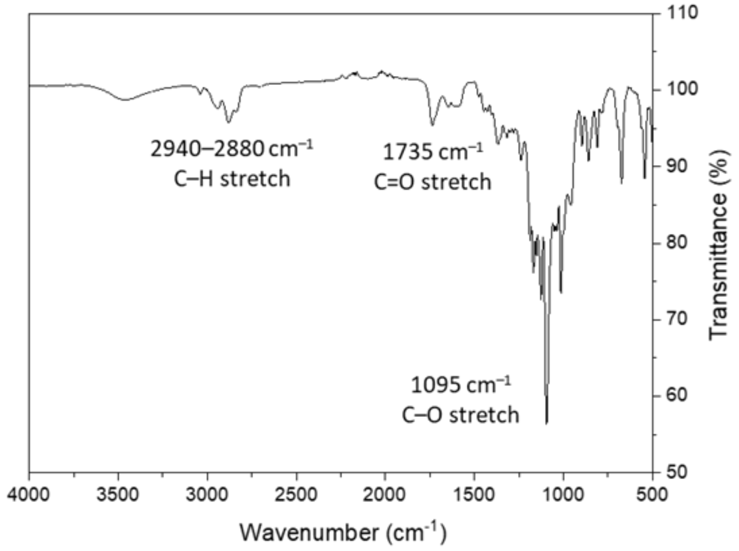

Figure S37. Labelled FT-IR spectrum of poly(**1**) (M_n 3,100 g mol^{-1} ($Đ_M$ 2.17), F_E/F_A = 23/77).

4. Free radical polymerization of 1 (in solution)

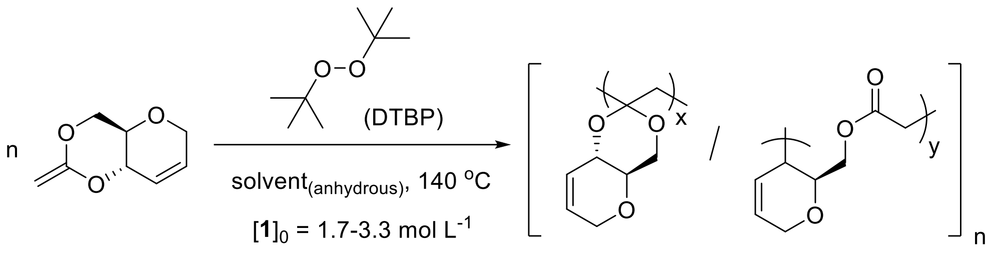

Under an argon atmosphere **1** (0.20 g, 1.30 mmol, 100 equiv.) was added into a Schlenk tube followed by three freeze-cooling-thaw cycles prior to the addition of 1,2-dichlorobenzene (0.26 mL, $[\mathbf{1}]_0$ = 1.7–3.3 mol L^{-1}) and di-tert-butyl-peroxide (DTBP) (2.4 µL, 0.013 mmol, 1 equiv.). The reaction vessel was maintained under an inert atmosphere, placed into an oil bath at 140 °C and stirred for 20 h. The reaction was quenched by rapid cooling and the solvent was removed under reduced pressure. The crude solid was dissolved in $CHCl_3$ and filtered before precipitation from excess hexane. The product was isolated by centrifugation (3500 rpm, 2 x 5 minutes), washed twice with hexane, and dried under vacuum. The polymer was isolated as a pale-brown solid (0.12 g, 60% yield).

δ_H (500 MHz; chloroform-d): 6.02-5.62 (C^2H, C^3H, 2H, m), 4.52-4.02 (C^1H, C^4H, 3H, m), 4.01-3.29 (C^5H, C^6H, 3H, m), 2.20-1.91 (C^8H, 2H, m) ppm; δ_C (126 MHz; chloroform-d): 171.9 (C^7), 170.9 (C^7), 129.5-124.7 (C^2, C^3), 112.8 (C^9), 77.0 (C^5), 65.7-65.3 (C^1), 64.4 (C^4), 63.6 (C^5), 21.2 (C^8), 21.0 (C^8) ppm. ν_{max} (cm^{-1}): 2998-2920 (CH), 1733 (C(O)O), 1063 (CO).

Table S2. Radical polymerization of **1** with DTBP, in solution.[a]

Entry	Solvent	$[\mathbf{1}]_0$:$[I]_0$[b]	$[\mathbf{1}]_0$ (mol L^{-1})	Time (h)	Conv.[c] (%)	$M_{n,SEC}$[d] [$Đ_M$] (kg mol^{-1})
1	p-Xylene	50:1	3.2	20	20	1.1 [1.35]
2	p-Xylene	100:1	4.7	20	42	1.2 [1.39]
3	p-Xylene	50:1	3.2	20	95	1.5 [1.94]
4	p-Xylene	100:1	4.7	20	99	1.6 [1.79]
5	ODCB	50:1	3.2	20	99	2.3 [2.49]
6	ODCB	100:1	4.7	20	99	4.7 [2.11]
7	ODCB	50:1	3.2	20	99	4.3 [2.33]
8	ODCB	100:1	4.7	20	99	5.1 [2.37]

[a]Reactions were carried out at 140 °C under an argon atmosphere, in anhydrous solvent with initial and $[\mathbf{1}]_0$ = 3.2–4.7 mol L^{-1} (**1** = monomer); [b]I = DTBP; [c]Monomer conversion to polymer, calculated based on the relative integration of the methylene proton signal of **1** (δ_H = 3.85 ppm, d, 2H) and the resultant alkyl proton signal(s) of poly(**1**) (δ_H = 2.85–2.28 and/or 2.17–2.02, m, 2H), in the ^1H NMR spectrum; [d]Number-average molar mass and Dispersity ($M_{n,SEC}$, $Đ_M$), calculated by SEC relative to polystyrene standards in THF eluent.

NMR analysis of poly(1) (solution polymerization)

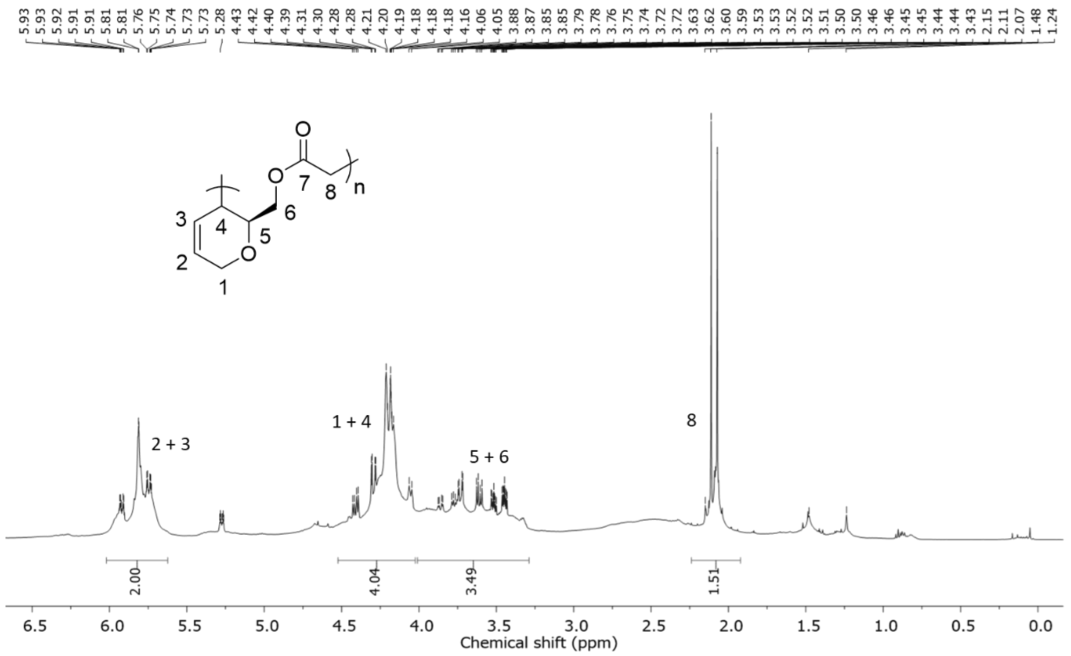

Figure S38. Annotated ^{1}H NMR spectrum ($CDCl_3$) of poly(**1**) (M_n 2,300 g mol^{-1} ($Đ_M$ 2.49), F_E/F_A = 78/22).

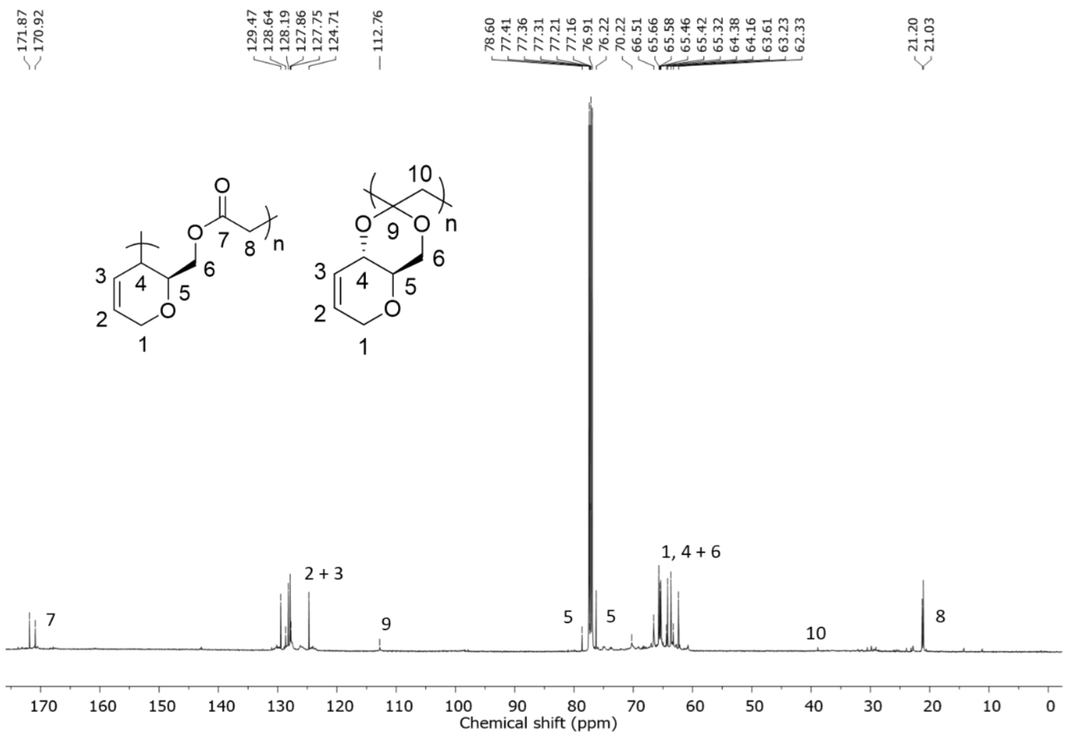

Figure S39. Annotated $^{13}C\{^{1}H\}$ NMR spectrum ($CDCl_3$) of poly(**1**) (M_n 2,300 g mol^{-1} ($Đ_M$ 2.49), F_E/F_A = 78/22).

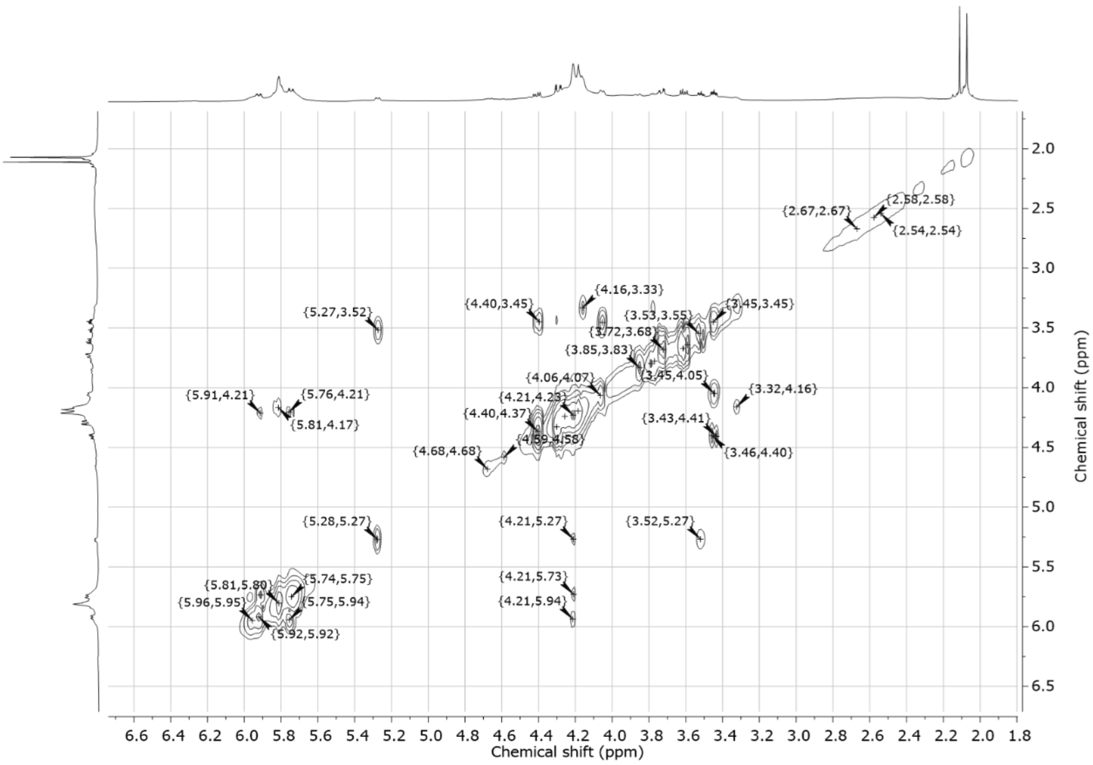

Figure S40. COSY (^1H–^1H) NMR spectrum ($CDCl_3$) of poly(**1**) (M_n 2,300 g mol^{-1} ($Đ_M$ 2.49), F_E/F_A = 78/22).

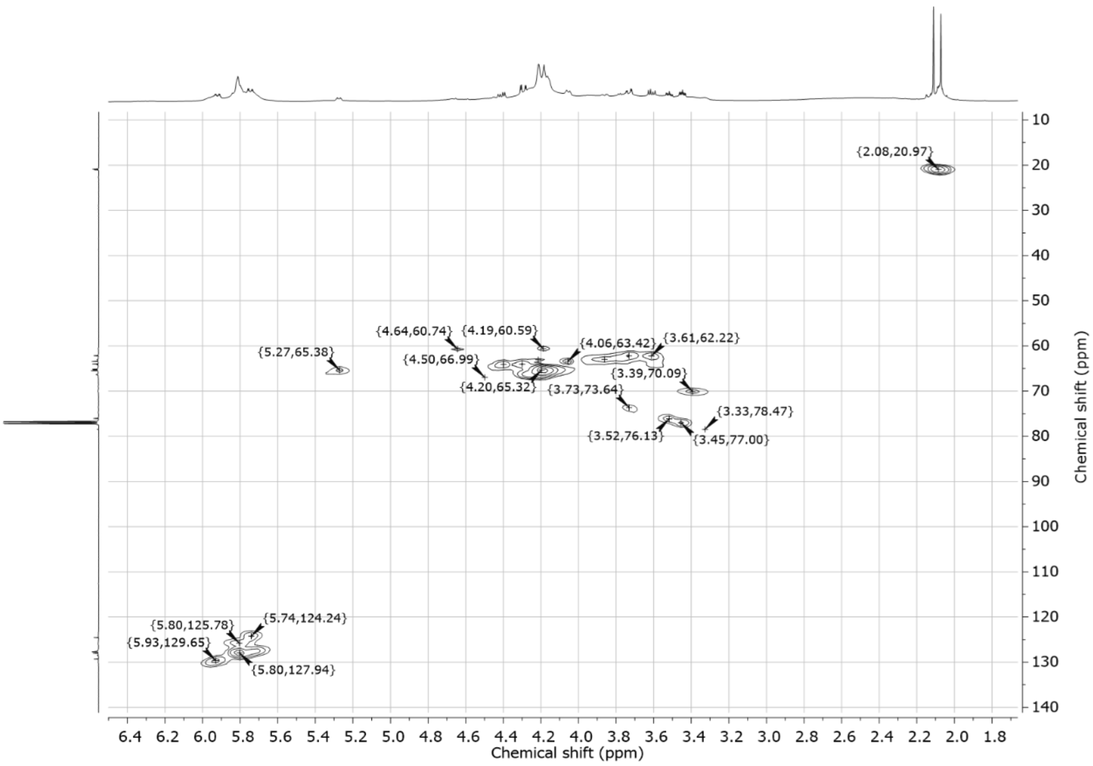

Figure S41. HSQC (^1H–^{13}C) NMR spectrum ($CDCl_3$) of poly(**1**) (M_n 2,300 g mol^{-1} ($Đ_M$ 2.49), F_E/F_A = 78/22).

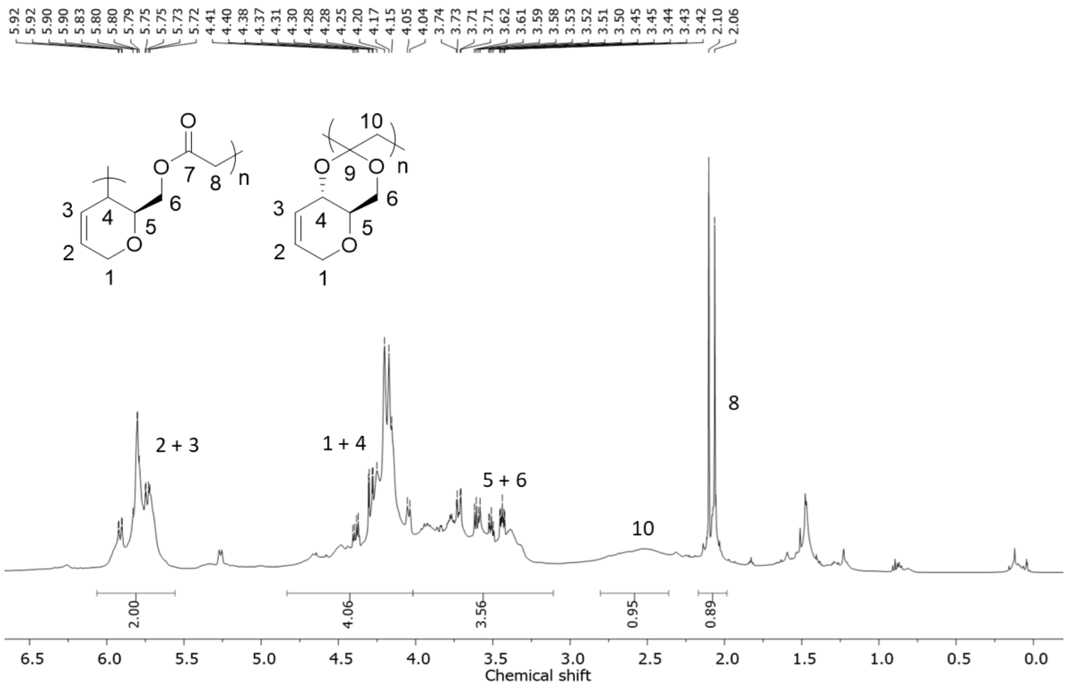

Figure S42. Annotated ^{1}H NMR spectrum ($CDCl_3$) of poly(**1**) (M_n 4,300 g mol^{-1} ($Đ_M$ 2.53), F_E/F_A = 54/44).

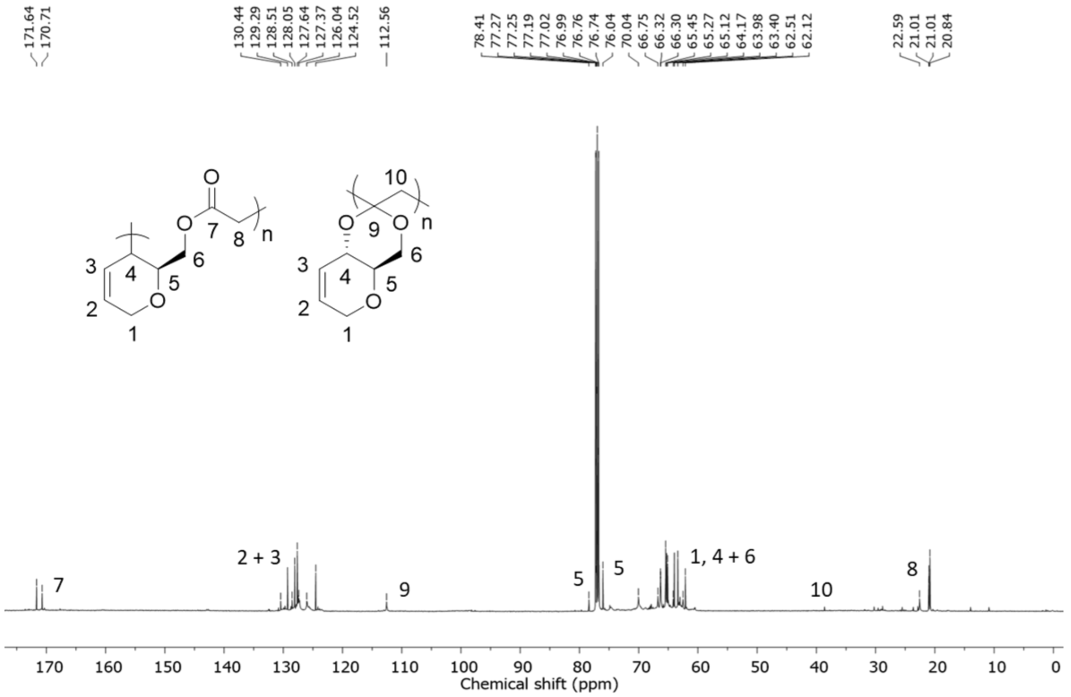

Figure S43. Annotated ^{13}C{^{1}H} NMR spectrum ($CDCl_3$) of poly(**1**) (M_n 4,300 g mol^{-1} ($Đ_M$ 2.53), F_E/F_A = 54/44).

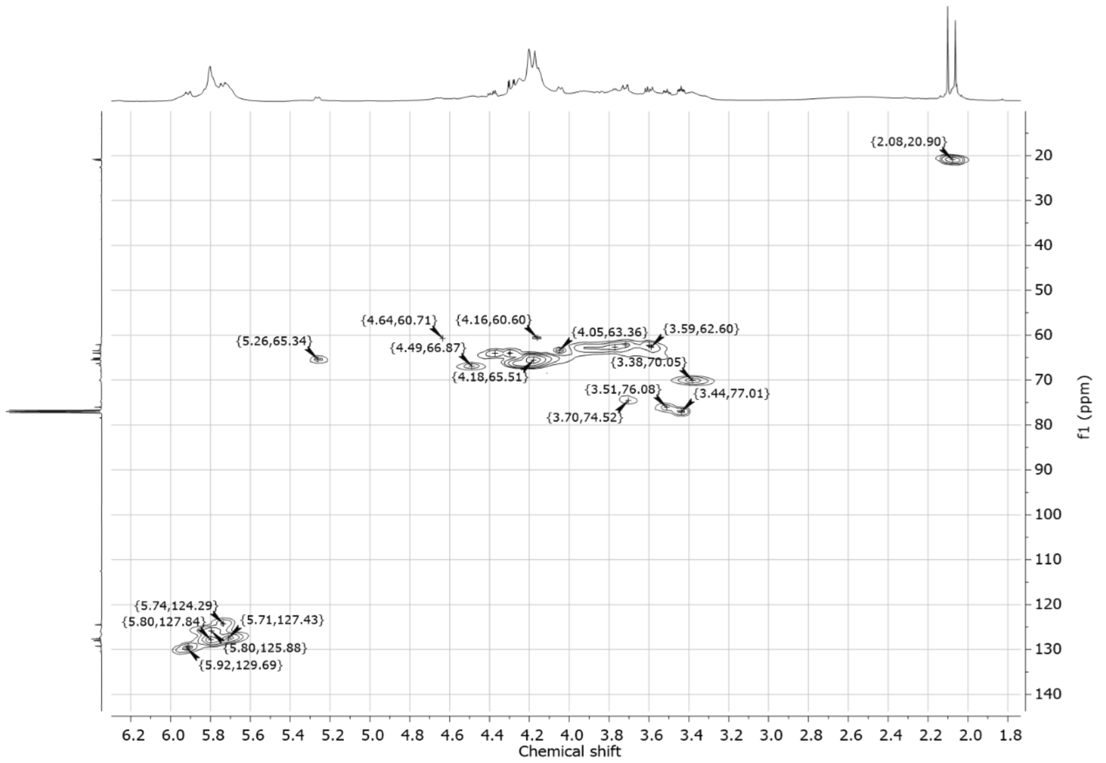

Figure S44. HSQC (^1H–^{13}C) NMR spectrum ($CDCl_3$) of poly(**1**) (M_n 4,300 g mol^{-1} ($Đ_M$ 2.53), F_E/F_A = 54/44).

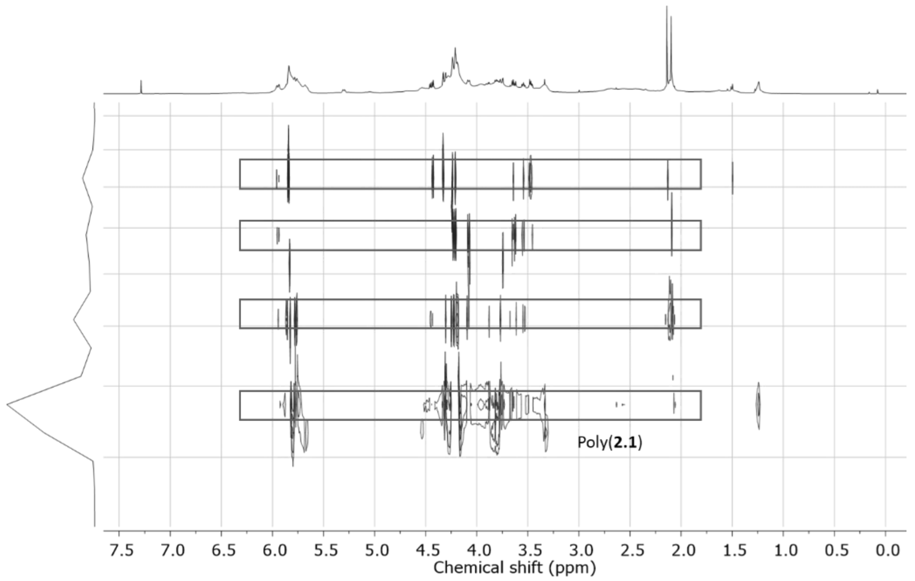

Figure S45. ^1H DOSY NMR spectrum ($CDCl_3$) of poly(**1**) (M_n 2,300 g mol^{-1} ($Đ_M$ 2.49), F_E/F_A = 78/22).

FT-IR analysis of poly(1) (solution polymerization)

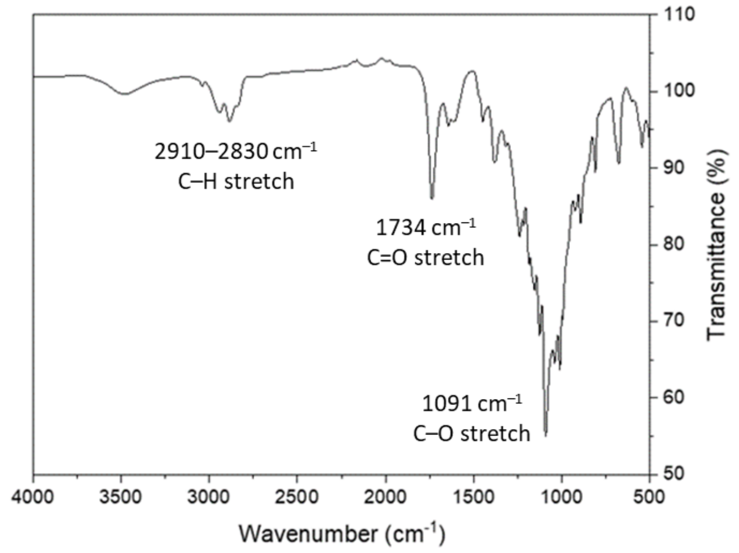

Figure S46. Labelled FT-IR spectrum of poly(**1**) (M_n 5,100 g mol^{-1} ($Đ_M$ 2.37), F_E/F_A = 43/57).

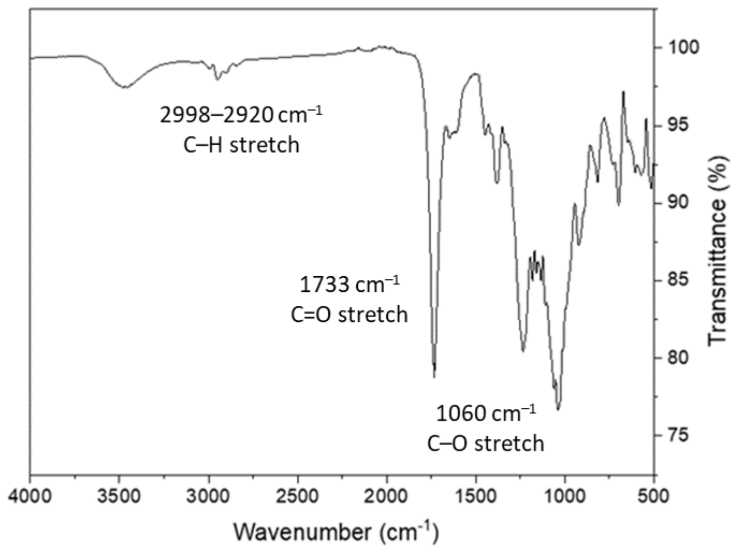

Figure S47. Labelled FT-IR spectrum of poly(**1**) (M_n 2,400 g mol^{-1} ($Đ_M$ 1.92), F_E/F_A = 78/22).

Representative size-exclusion chromatography analysis of poly(1)

Molecular Weight Averages

Peak	Mp (g/mol)	Mn (g/mol)	Mw (g/mol)	Mz (g/mol)	Mz+1 (g/mol)	Mv (g/mol)	PD
Peak 1	3810	5112	12127	25445	40901	23311	2.372

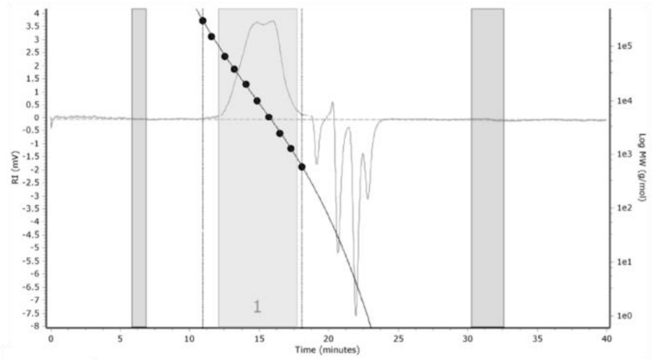

Figure S48. SEC trace of poly(**1**) (M_n 5,100 g mol^{-1} ($Đ_M$ 2.37), F_E/F_A = 43/57), in THF.

Molecular Weight Averages

Peak	Mp (g/mol)	Mn (g/mol)	Mw (g/mol)	Mz (g/mol)	Mz+1 (g/mol)	Mv (g/mol)	PD
Peak 1	4259	4667	9867	21091	34314	19201	2.114

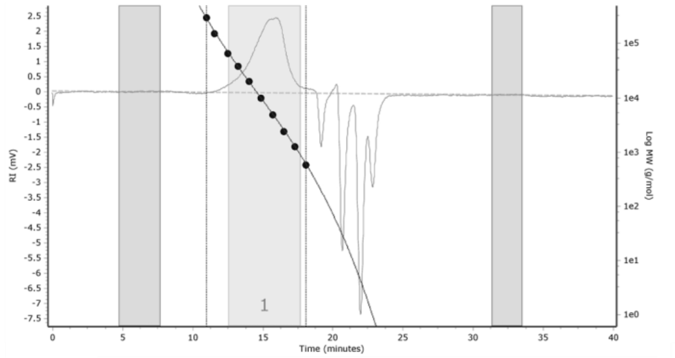

Figure S49. SEC trace of poly(**1**) (M_n 4,700 g mol^{-1} ($Đ_M$ 2.11), F_E/F_A = 65/35), in THF.

MALDI ToF MS analysis of poly(1)

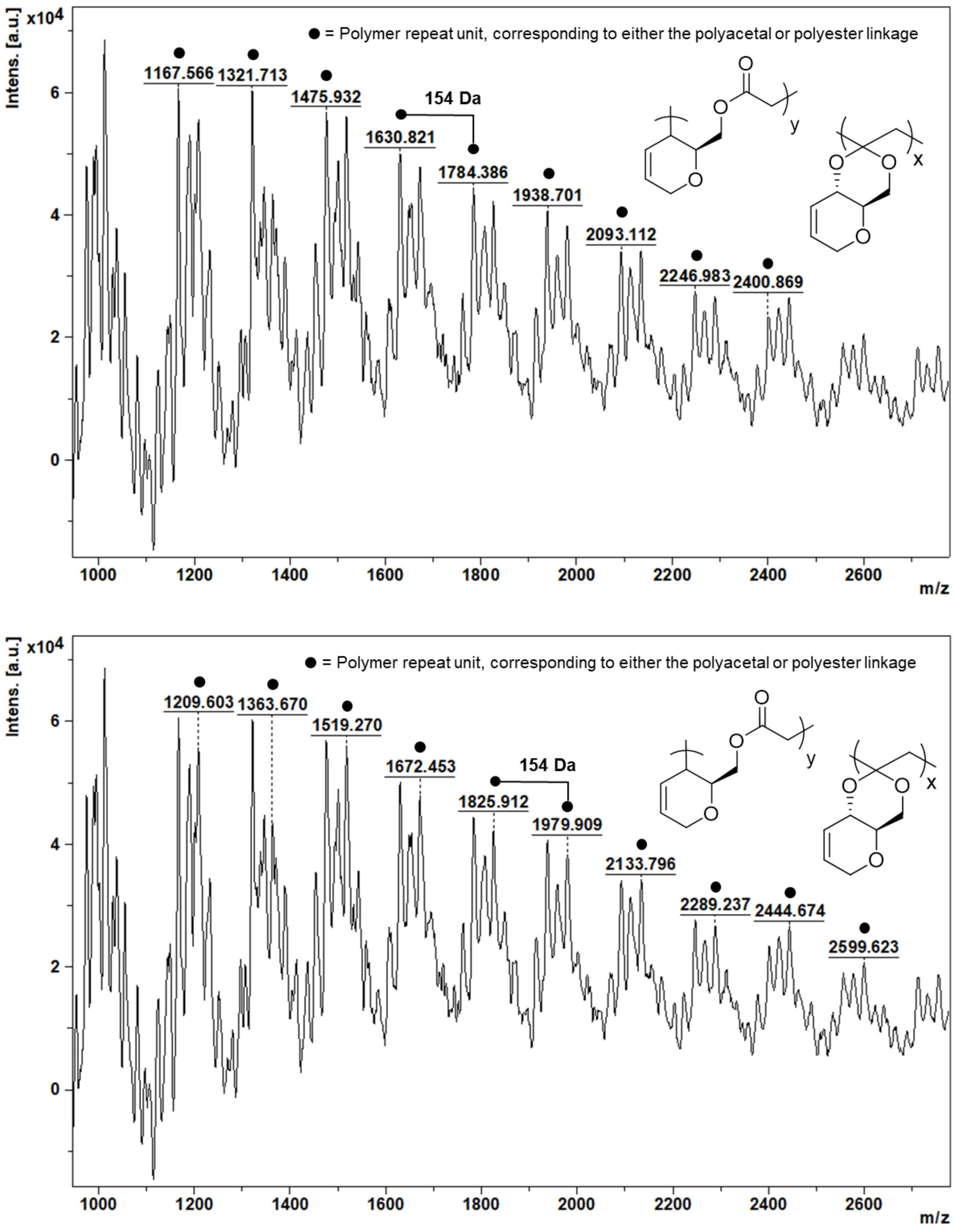

Figure S50. MALDI-TOF MS spectrum of poly(**1**) (M_n 4,300 g mol^{-1} ($Đ_M$ 2.53), F_E/F_A = 56/44), obtained in linear positive ionisation mode, showing the repeat unit of m/z 154. The MALDI-TOF MS spectrum has been smoothed and has had a baseline correction. Both spectra shown are the same but at the top: the second peak of the repeating set of peaks has been picked; whilst at the bottom, the fourth peak has been picked. Both approaches show the same value for the polymer repeat unit. No end group was identifiable.

5. Thermal analysis of poly(1)

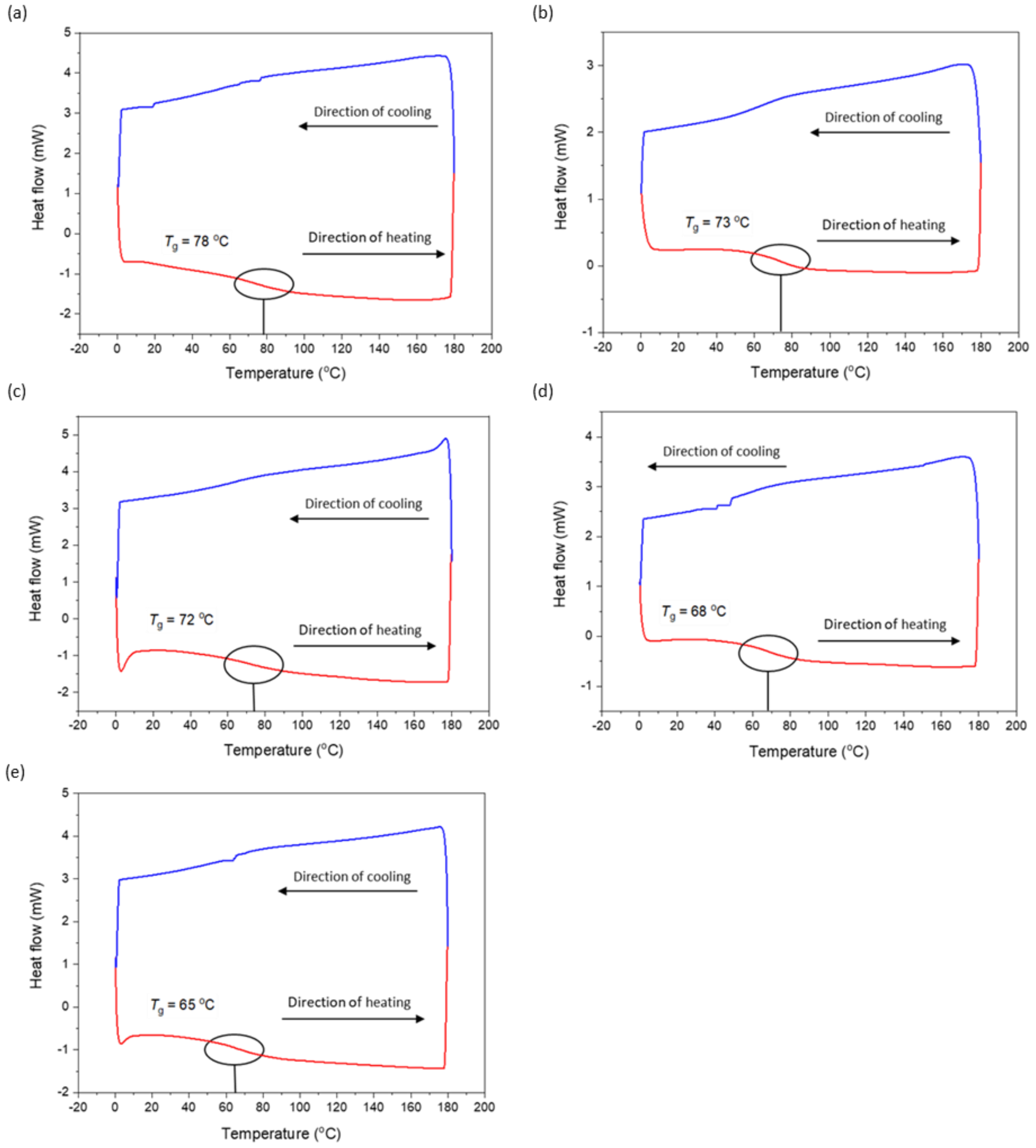

Figure S51. DSC traces (second heating and cooling cycle) of (a) poly(**1**) (M_n 3,100 g mol^{-1} ($Đ_M$ 2.17), F_E/F_A = 23/77), T_g = 78 °C. (b) poly(**1**) (M_n 5,100 g mol^{-1} ($Đ_M$ 2.37), F_E/F_A = 43/57), T_g = 73 °C. (c) poly(**1**) (M_n 4,300 g mol^{-1} ($Đ_M$ 2.53), F_E/F_A = 54/44), T_g = 72 °C. (d) poly(**1**) (M_n 4,700 g mol^{-1} ($Đ_M$ 2.11), F_E/F_A = 65/35), T_g = 68 °C. (e) poly(**1**) (M_n 2,400 g mol^{-1} ($Đ_M$ 1.92), F_E/F_A = 78/22, T_g = 65 °C. Exothermic and endothermic events are indicated by positive and negative heat flows, respectively.

Table S3. Polymeric data and thermal properties of polymers derived from **1**, poly(**1**)s.

Entry	F_E/F_A[a]	$M_{n,SEC}$[a] [$Đ_M$] (kg mol^{-1})	T_g[c](°C)	$T_{d5\%}$[d] (°C)	$T_{d,max}$[d] (°C)	% Mass loss[d]
1	23/77	3.1 [2.17]	78	206	220	72
2	43/57	5.1 [2.37]	73	230	241	69
3	56/44	4.3 [2.53]	72	218	233	72
4	65/35	4.7 [2.11]	68	210	293	73
5	78/22	1.2 [1.44]	-	205	278	70

[a]Monomer selectivity between polyester (F_E) and polyacetal (F_A) linkages determined by integration of the $^{13}C\{^1H\}$ NMR spectra of the purified polymers; [b]Number-average molar mass and Dispersity ($M_{n,SEC}$, $Đ_M$), calculated by SEC relative to polystyrene standards in THF eluent; [c]Values obtained from DSC second heating cycle; [d]Values obtained from TGA analysis of the polymers, heated from 30 to 600 (or 700 °C) under argon at 10 °C min^{-1}.

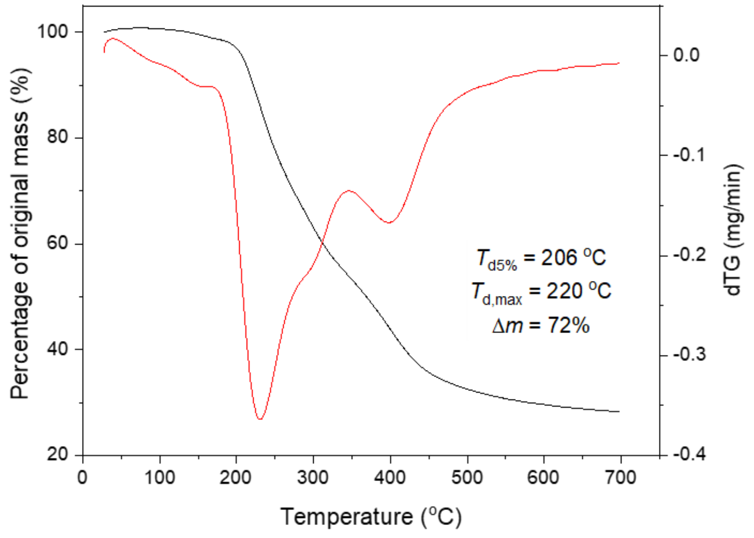

Figure S52. TGA trace of poly(**1**) (M_n 3,100 g mol^{-1} ($Đ_M$ 2.17), F_E/F_A = 23/77). The polymer was heated from 30 to 700 °C under argon at 10 °C min^{-1}. Obtained values: $T_{d5\%}$ = 206 °C, $T_{d,max}$ = 220 °C with 28 % char remaining at 700 °C.

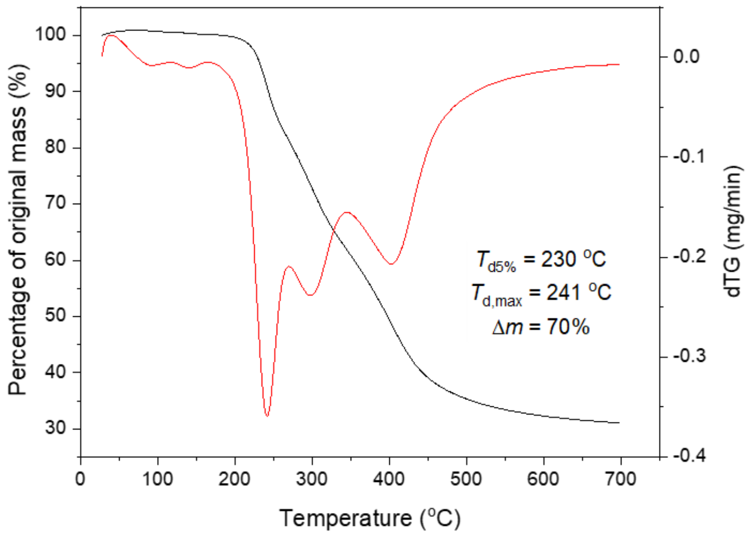

Figure S53. TGA trace of poly(**1**) (M_n 5,100 g mol^{-1} ($Đ_M$ 2.37), F_E/F_A = 43/57). The polymer was heated from 30 to 700 °C under argon at 10 °C min^{-1}. Obtained values: $T_{d5\%}$ = 230 °C, $T_{d,max}$ = 241 °C with 30 % char remaining at 700 °C.

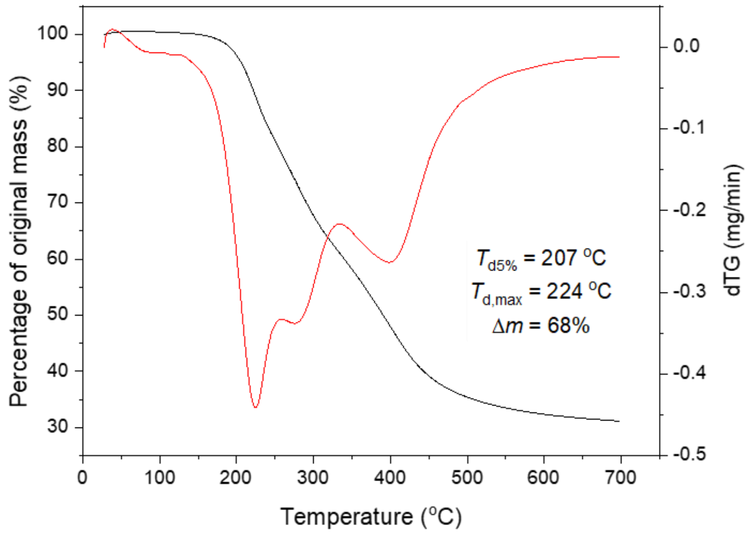

Figure S54. TGA trace of poly(**1**) (M_n 4,300 g mol^{-1} ($Đ_M$ 2.53), F_E/F_A = 54/44)). The polymer was heated from 30 to 700 °C under argon at 10 °C min^{-1}. Obtained values: $T_{d5\%}$ = 207 °C, $T_{d,max}$ = 224 °C with 32 % char remaining at 700 °C.

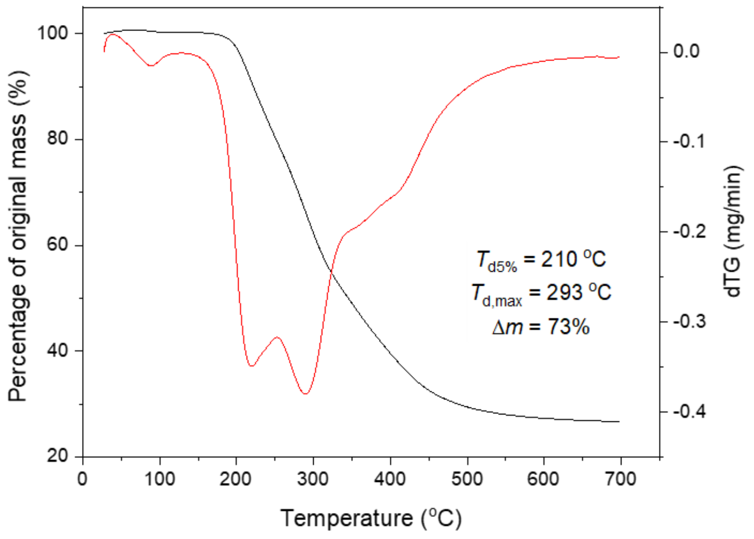

Figure S55. TGA trace of poly(**1**) (M_n 4,700 g mol^{-1} ($Đ_M$ 2.11), F_E/F_A = 65/35). The polymer was heated from 30 to 700 °C under argon at 10 °C min^{-1}. Obtained values: $T_{d5\%}$ = 210 °C, $T_{d,max}$ = 293 °C with 27 % char remaining at 700 °C.

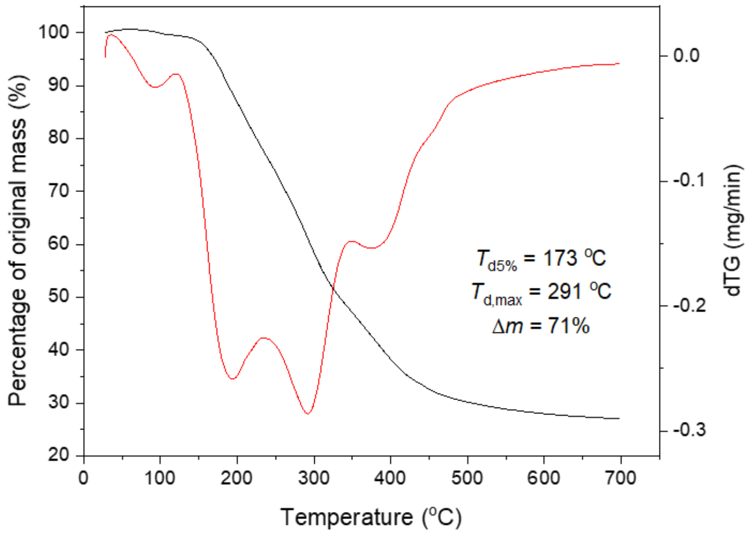

Figure S56. TGA trace of poly(**1**) (M_n 1,200 g mol^{-1} ($Đ_M$ 1.44), F_E/F_A = 78/22). The polymer was heated from 30 to 700 °C under argon at 10 °C min^{-1}. Obtained values: $T_{d5\%}$ = 173 °C, $T_{d,max}$ = 291 °C with 29 % char remaining at 700 °C.

6. Free radical polymerization of 2 (in solution)

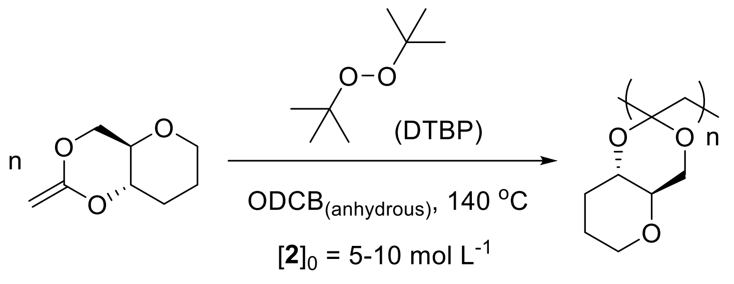

Under an an argon atmosphere **2** (0.20 g, 1.28 mmol, 100 equiv.) was added into a Schlenk tube followed by three freeze-cooling-thaw cycles prior to the addition of 1,2-dichlorobenzene (0.260 mL, [**2**]$_0$ = 5–10 mol L^{-1}) and di-tert-butyl-peroxide (DTBP) (2.4 µL, 0.013 mmol, 1 equiv.). The reaction vessel was maintained under an inert atmosphere, placed into an oil bath at 140 °C and stirred for 20 h. The reaction was quenched by rapid cooling and the solvent was removed under reduced pressure. The crude solid was dissolved in $CHCl_3$ and filtered before precipitation from excess hexane. The product was isolated by centrifugation (3500 rpm, 2 x 5 minutes), washed twice with hexane, and dried under vacuum. The polymer was isolated as a pale-brown solid.

δH (500 MHz; chloroform-d): 4.11-3.66 (C^1H, C^5H, C^6H, 3H, m), 3.65-2.86 (C^1H, C^4H, C^6H, 3H, m), 2.72-2.06 (C^8H, 2H, m), 2.04-1.21 (C^2H, C^3H, 4H) ppm; δ_C (126 MHz; chloroform-d): 101.3 (C^7), 74.7 (C^4), 70.7 (C^5), 68.0 (C^1), 63.5 (C^6), 40.9 (C^8), 29.2 (C^3), 25.3 (C^2) ppm. ν_{max} (cm^{-1}): 2900-2830 (CH), 1089 (CO).

NMR analysis of poly(2) (solution polymerization)

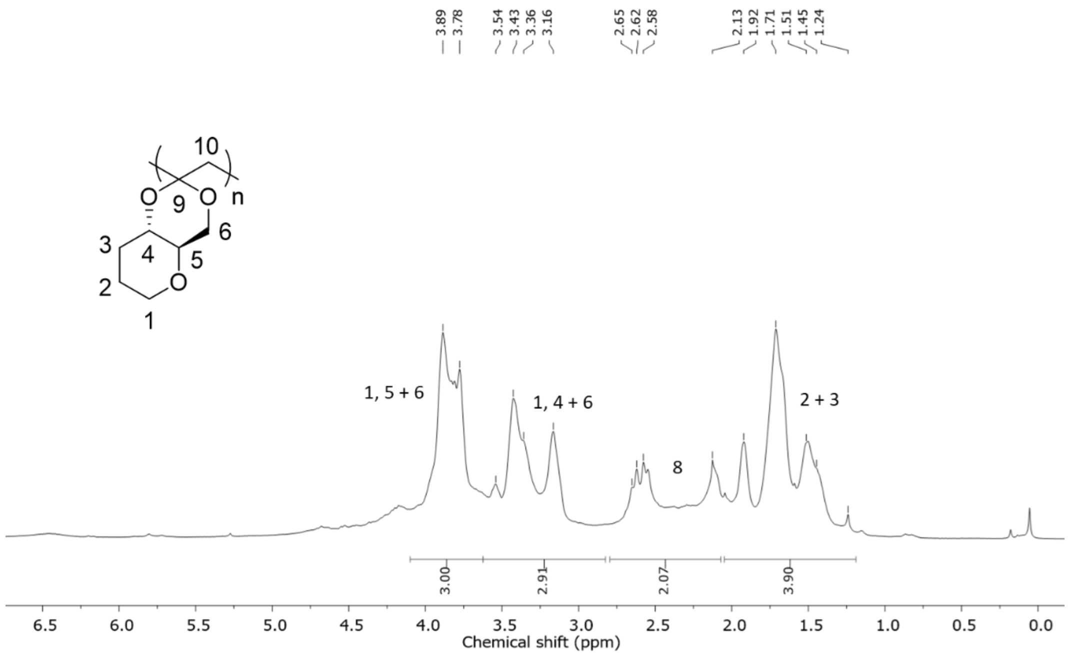

Figure S57. Annotated ^1H NMR spectrum ($CDCl_3$) of poly(**2**) (M_n 1,000 g mol^{-1} ($Đ_M$ 1.66), F_E/F_A = 0/100).

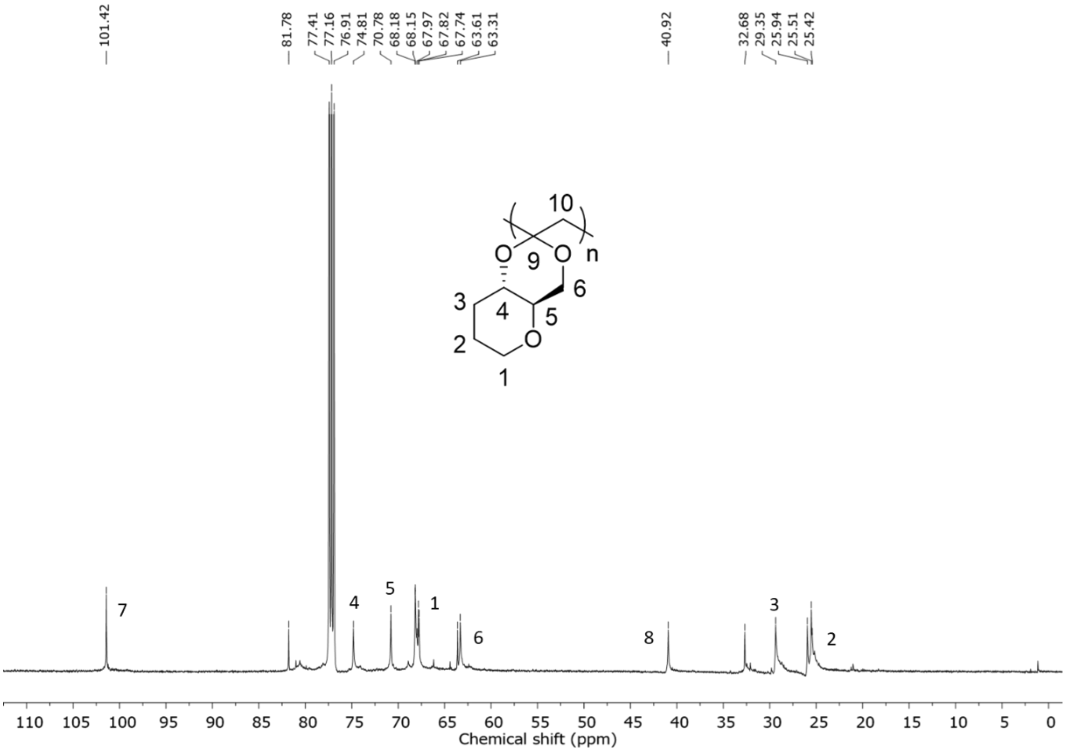

Figure S58. Annotated $^{13}C\{^{1}H\}$ NMR spectrum ($CDCl_3$) of poly(**2**) (M_n 1,000 g mol^{-1} ($Đ_M$ 1.66), F_E/F_A = 0/100).

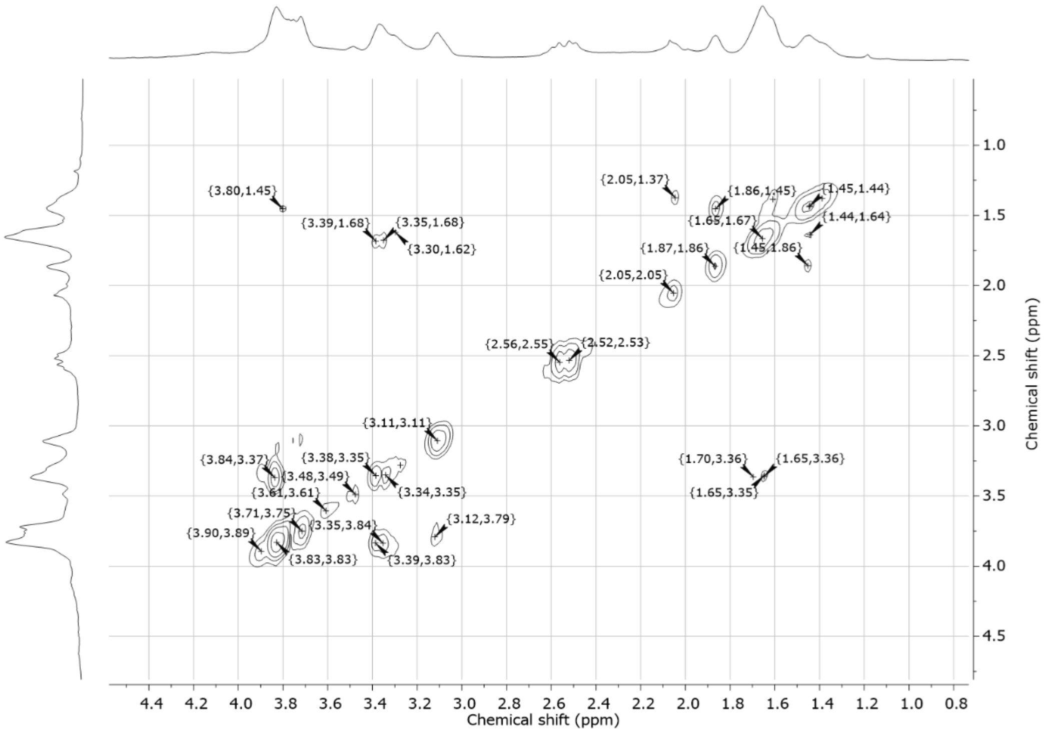

Figure S59. COSY (^{1}H–^{1}H) NMR spectrum ($CDCl_3$) of poly(**2**) (M_n 1,000 g mol^{-1} ($Đ_M$ 1.66), F_E/F_A = 0/100).

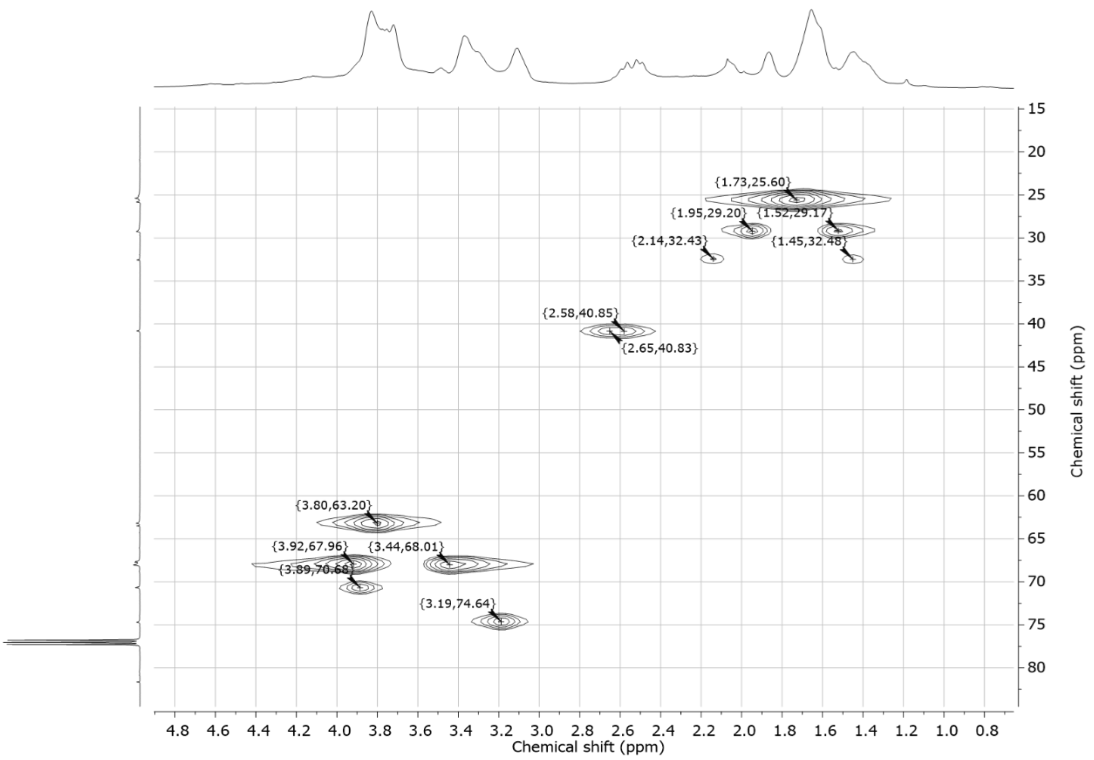

Figure S60. HSQC (^1H–^{13}C) NMR spectrum ($CDCl_3$) of poly(**2**) (M_n 1,000 g mol^{-1} ($Đ_M$ 1.66), F_E/F_A = 0/100).

FT-IR analysis of poly(2) (solution polymerization)

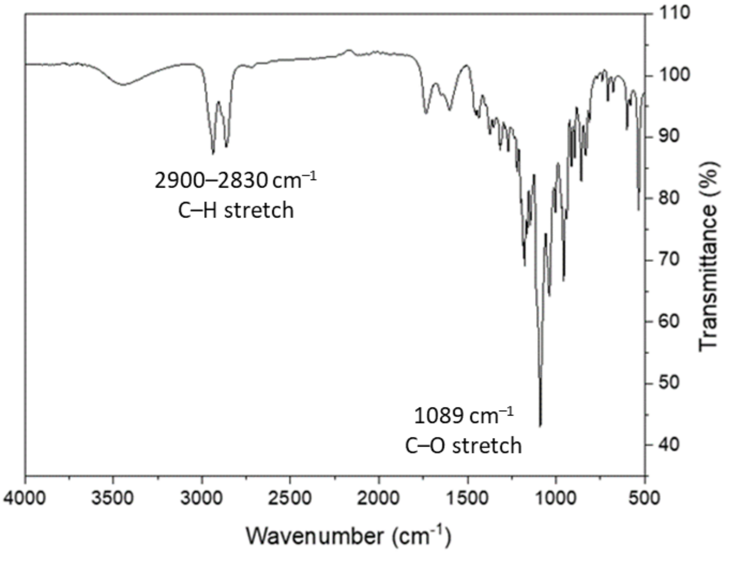

Figure S61. Labelled FT-IR spectrum of poly(**2**) (M_n 1,000 g mol^{-1} ($Đ_M$ 1.66), F_E/F_A = 0/100).

Representative size-exclusion chromatography analysis of poly(2)

Molecular Weight Averages

Peak	Mp (g/mol)	Mn (g/mol)	Mw (g/mol)	Mz (g/mol)	Mz+1 (g/mol)	Mv (g/mol)	PD
Peak 1	1183	1002	1666	2640	3768	2484	1.663

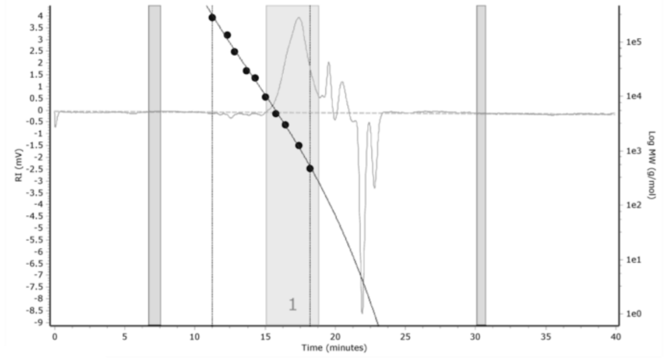

Figure S62. SEC trace of poly(**2**) (M_n 1,000 g mol^{-1} ($Đ_M$ 1.66), F_E/F_A = 0/100), in THF.

Molecular Weight Averages

Peak	Mp (g/mol)	Mn (g/mol)	Mw (g/mol)	Mz (g/mol)	Mz+1 (g/mol)	Mv (g/mol)	PD
Peak 1	1431	1386	2537	4255	5988	3997	1.83

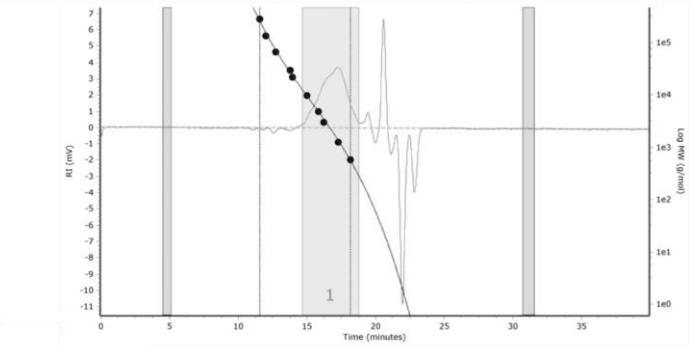

Figure S63. SEC trace of poly(**2**) (M_n 1,400 g mol^{-1} ($Đ_M$ 1.83), F_E/F_A = 0/100), in THF.

Thermal analysis of poly(2)

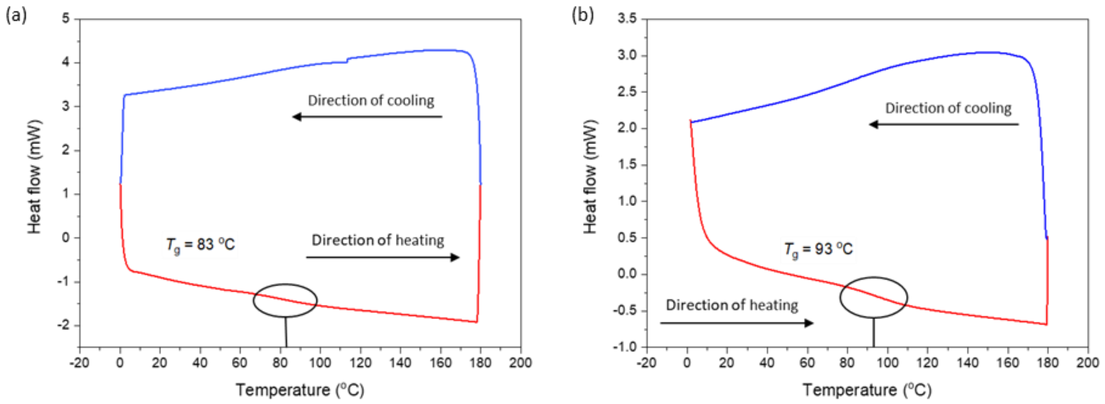

Figure S64. DSC traces (second heating and cooling cycle) of (a) poly(**2**) (M_n 1,000 g mol^{-1} ($Đ_M$ 1.66), F_E/F_A = 0/100), T_g = 83 °C. (b) poly(**2**) (M_n 1,400 g mol^{-1} ($Đ_M$ 1.83), F_E/F_A = 0/100), T_g = 93 °C. Exothermic and endothermic events are indicated by positive and negative heat flows, respectively.

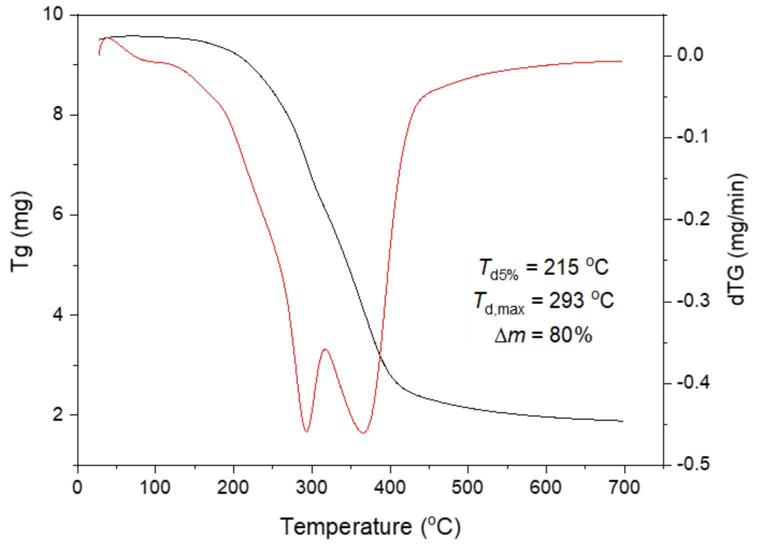

Figure S65. TGA trace of poly(**2**) (M_n 1,000 g mol^{-1} ($Đ_M$ 1.66), F_E/F_A = 0/100). The polymer was heated from 30 to 700 °C under argon at 10 °C min^{-1}. Obtained values: $T_{d5\%}$ = 215 °C, $T_{d,max}$ = 293 °C with 20 % char remaining at 700 °C.

7. One-pot copolymerization of 1 with methyl methacrylate

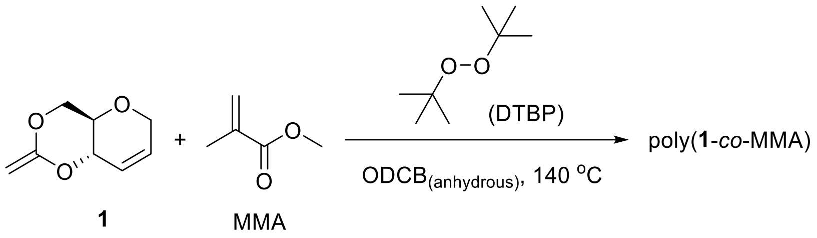

Under an argon atmosphere **1** (0.20 g, 1.30 mmol, 50 equiv.) and methyl methacrylate (0.14 mL, 1.30 mmol, 50 equiv.) were added into a Schlenk tube followed by three freeze-cooling-thaw cycles prior to the addition of anhydrous 1,2-dichlorobenzene (0.13 mL) and di-tert-butyl-peroxide (DTBP) (4.8 µL, 0.026 mmol, 1 equiv.). The reaction vessel was maintained under an inert atmosphere, placed into an oil bath at 140 °C and stirred for 20 h. After completion, the reaction was quenched by rapid cooling and the solvent was removed under reduced pressure. The crude solid was dissolved in $CHCl_3$ and filtered before precipitation from excess hexane. The precipitate was then isolated by centrifugation (3500 rpm, 2 x 5 minutes), dried under vacuum, re-dissolved in $CHCl_3$ and precipitated from excess Et_2O. The product was isolated by centrifugation (3500 rpm, 2 x 5 minutes), as a pale-brown solid and dried under vacuum (0.228 g, 69% yield). Additionally, the supernatant of the second precipitation was concentrated in *vacuo*, dissolved in $CHCl_3$, and precipitated from excess hexane as a pale-brown solid ("secondary" polymer) (0.067 g, 20% yield). Total isolated polymer yield = 89%.

δ_H (500 MHz; chloroform-d): 6.01-5.53 ($C^{11}H$, $C^{12}H$, 2H, m), 4.57-3.25 (C^4H, C^8H, C^9H, $C^{10}H$, $C^{13}H$, 14H, m), 2.82-2.36 (C^6H, 2H, m), 2.20-1.74 (C^1H, 5H, m), 1.33-0.77 (C^5H, 8H, m) ppm; δ_C (126 MHz; chloroform-d): 178.5-177.0 (C^3), 171.8 (C^7), 170.9 (C^7), 129.5-124.7 (C^{11}, C^{12}), 103.4 (C^{14}), 101.7 (C^{14}), 77.2-76.2 (C^9), 70.1 (C^9), 66.5 (C^1), 65.7-62.4 (C^8, C^9, C^{10}, C^{13}), 51.9 (C^4), 45.0-43.4 (C^2), 35.2-30.8 (C^6), 18.7-16.3 (C^5) ppm;. ν_{max} (cm^{-1}): 2950 (CH), 1727 (C(O)O), 1116-1094 (CO)

NMR analysis of poly(1-*co*-MMA)

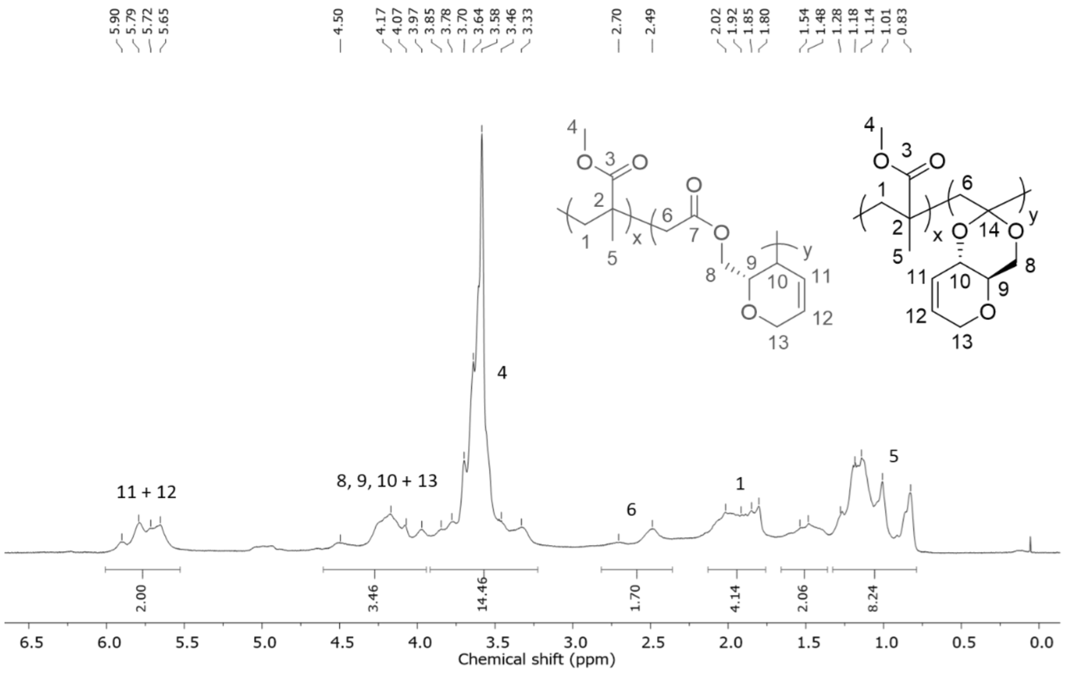

Figure S66. Annotated ^{1}H NMR spectrum ($CDCl_3$) of poly(**1**-*co*-MMA) (M_n 12,600 g mol^{-1} ($Đ_M$ 2.53), F_1/F_{MMA} = 33/67).

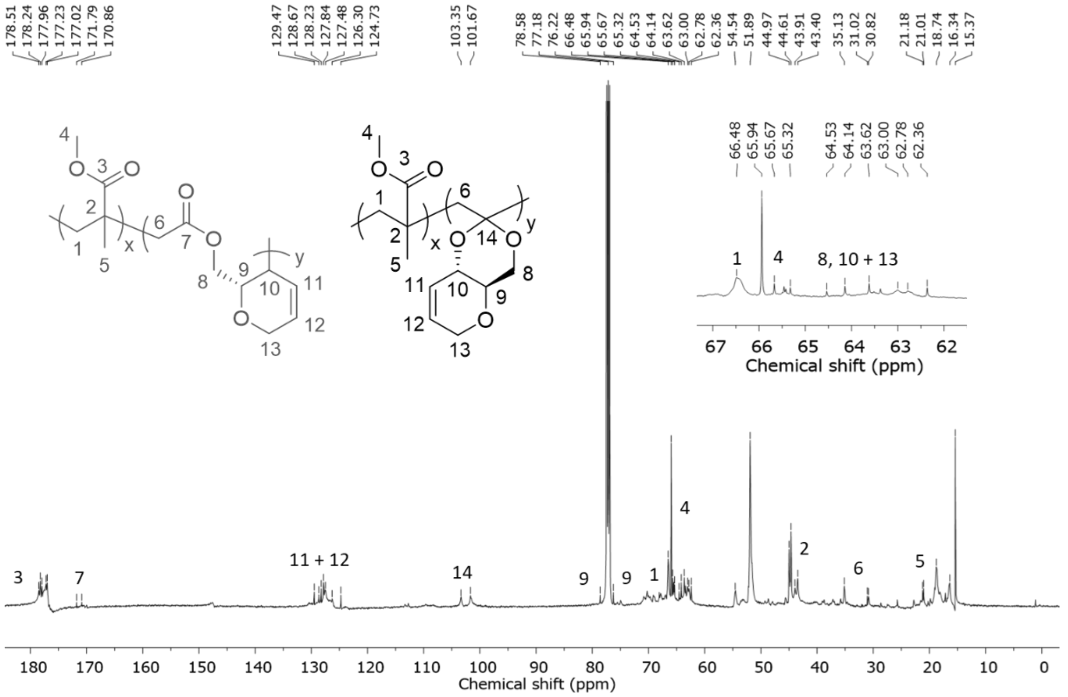

Figure S67. Annotated $^{13}C\{^{1}H\}$ NMR spectrum ($CDCl_3$) of poly(**1**-co-MMA) (M_n 12,600 g mol^{-1} ($Đ_M$ 2.53), F_1/F_{MMA} = 33/67).

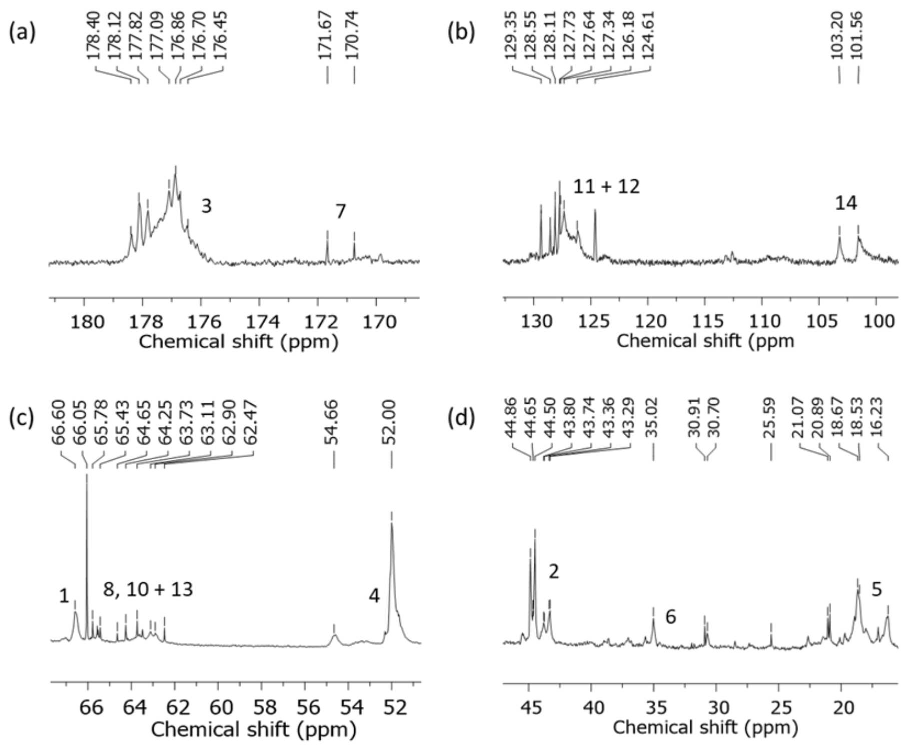

Figure S68. Localized and annotated $^{13}C\{^1H\}$ NMR spectra of poly(**1**-co-MMA) (M_n 12,600 g mol^{-1} ($Đ_M$ 2.53), F_1/F_{MMA} = 33/67) in chloroform-d. $^{13}C\{^1H\}$ NMR spectra show: (a) δ_C = 185–170, (b) δ_C = 135–100, (c) δ_C = 67–51 and (d) δ_C = 45–15 ppm regions.

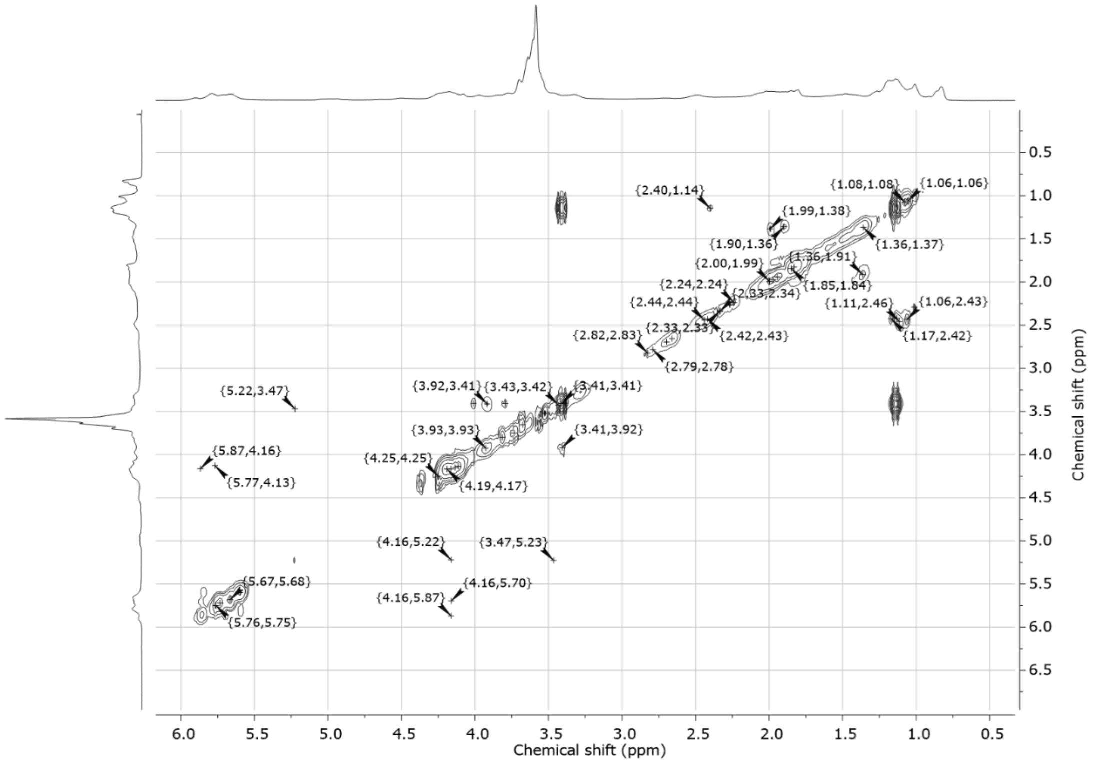

Figure S69. COSY (^1H–^1H) NMR spectrum ($CDCl_3$) of poly(1-co-MMA) (M_n 12,600 g mol^{-1} ($Đ_M$ 2.53), F_1/F_{MMA} = 33/67).

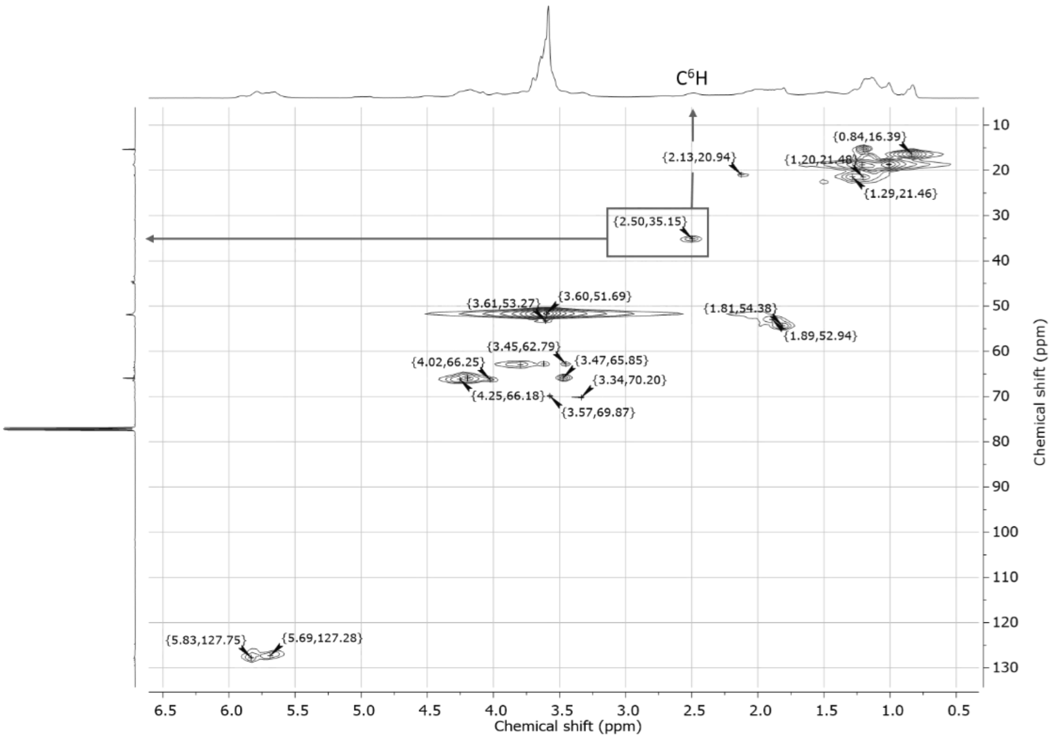

Figure S70. HSQC (^{1}H–^{13}C) NMR spectrum ($CDCl_3$) of poly(**1**-co-MMA) (M_n 12,600 g mol^{-1} ($Đ_M$ 2.53), F_1/F_{MMA} = 33/67).

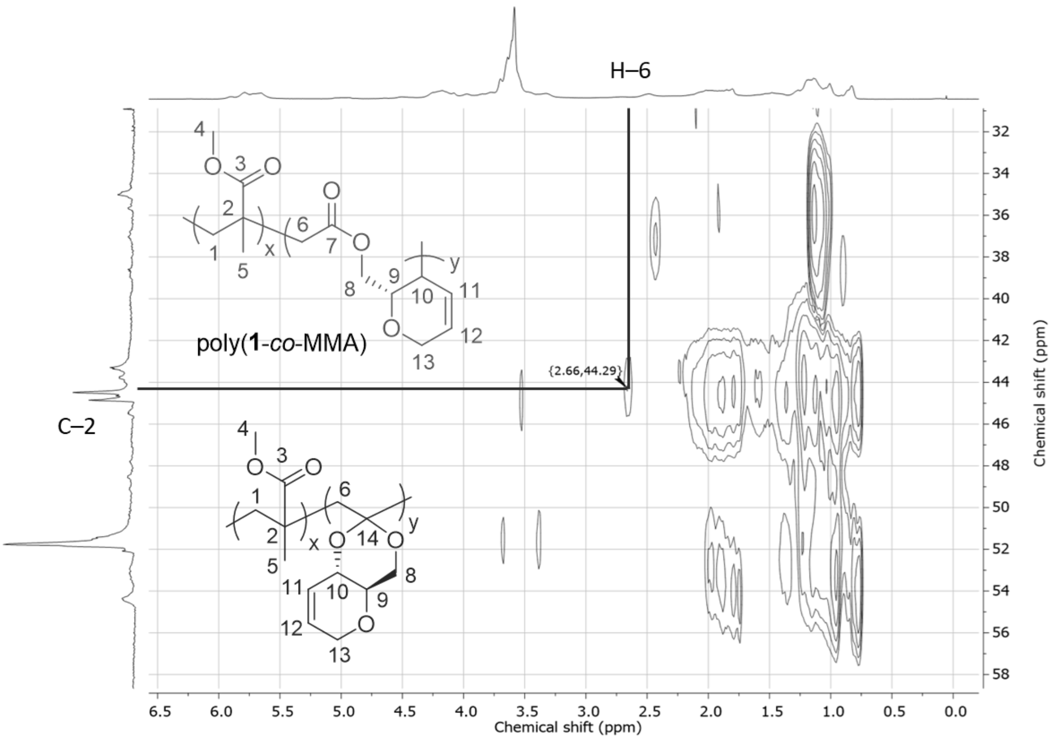

Figure S71. Focused HMBC (^{1}H–^{13}C) NMR spectrum ($CDCl_3$) of poly(**1**-co-MMA) (M_n 12,600 g mol^{-1} ($Đ_M$ 2.53), F_1/F_{MMA} = 33/67). Interaction between H^6 of **1** and C^2 of MMA is highlighted.

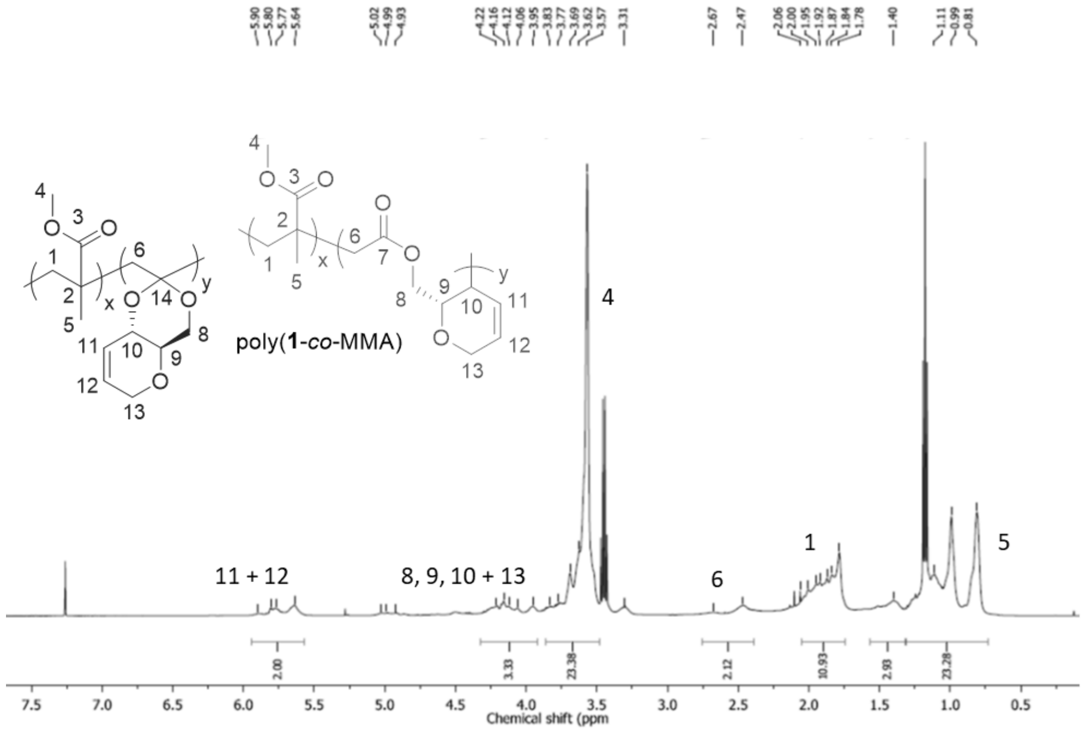

Figure S72. Annotated ^{1}H NMR spectrum ($CDCl_3$) of poly(**1**-*co*-MMA) (M_n 24,400 g mol^{-1} ($Đ$ 2.01), F_1/F_{MMA} = 16/84).

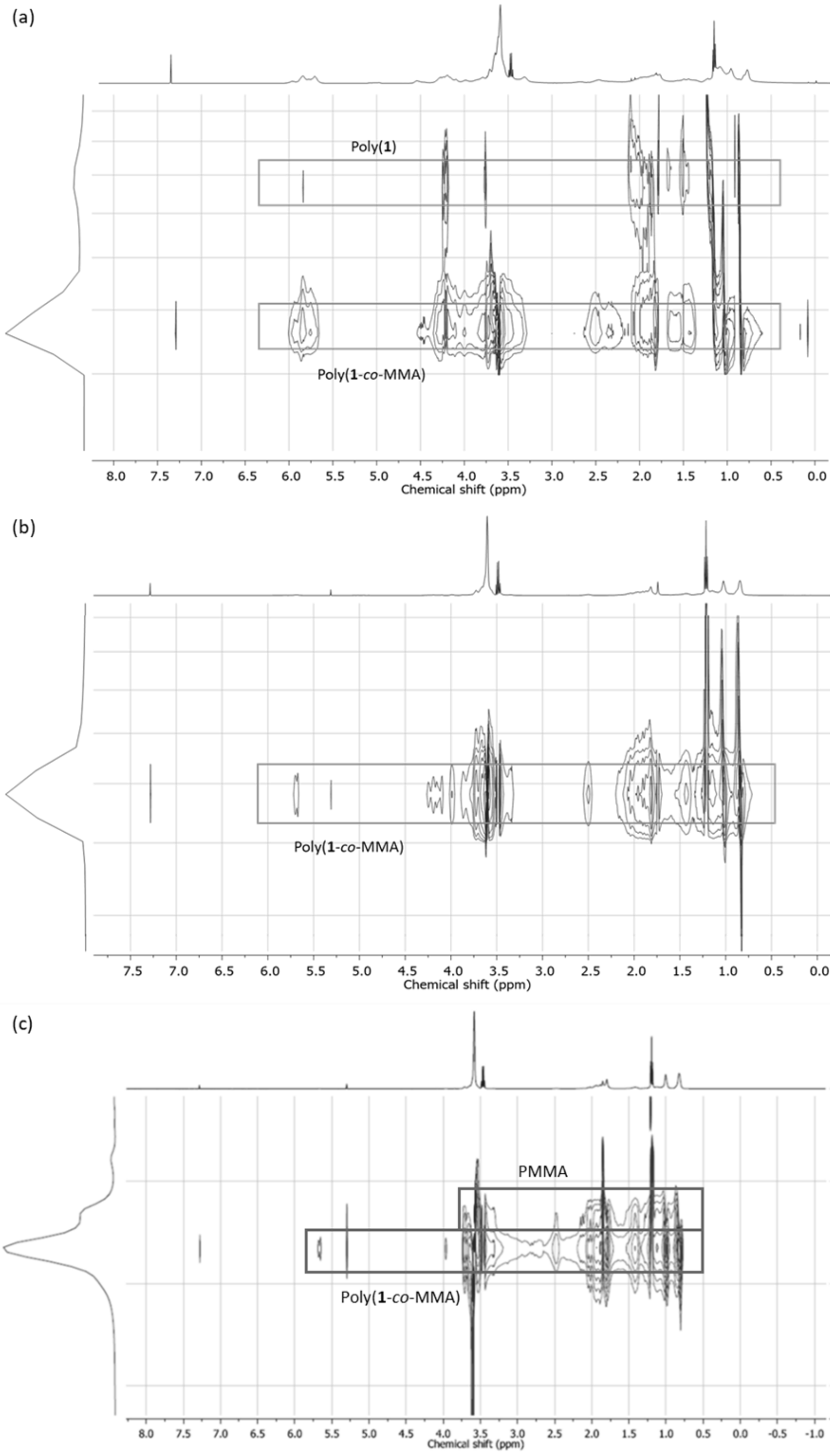

Figure S73. ^{1}H DOSY NMR spectra ($CDCl_3$) of (a) poly(**1**-*co*-MMA) (M_n 12,600 g mol^{-1} ($Đ_M$ 2.53), F_1/F_{MMA} = 33/67), (b) poly(**1**-*co*-MMA) (M_n 24,400 g mol^{-1} ($Đ_M$ 2.01), F_1/F_{MMA} = 16/84), and (c) poly(**1**-*co*-MMA) (M_n 39,900 g mol^{-1} ($Đ_M$ 2.59), F_1/F_{MMA} = 2/98).

FT-IR analysis of poly(1-*co*-MMA)

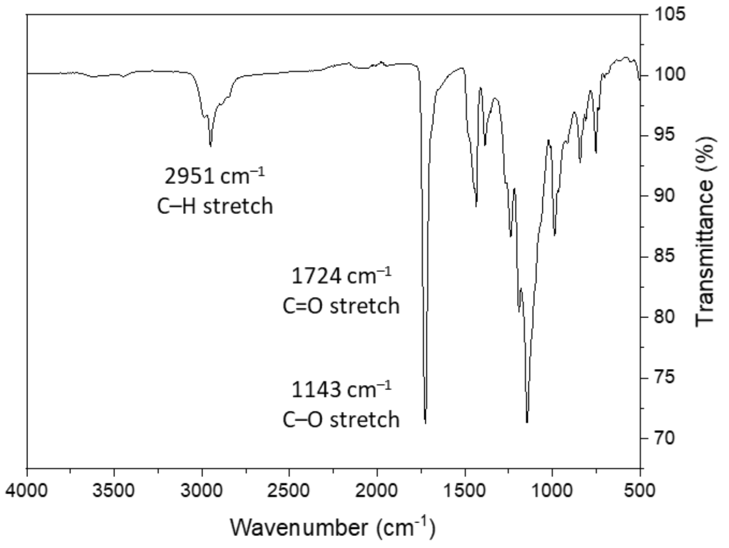

Figure S74. Labelled FT-IR spectrum of poly(**1**-*co*-MMA) (M_n 39,900 g mol^{-1} ($Đ_M$ 2.59), F_1/F_{MMA} = 2/98).

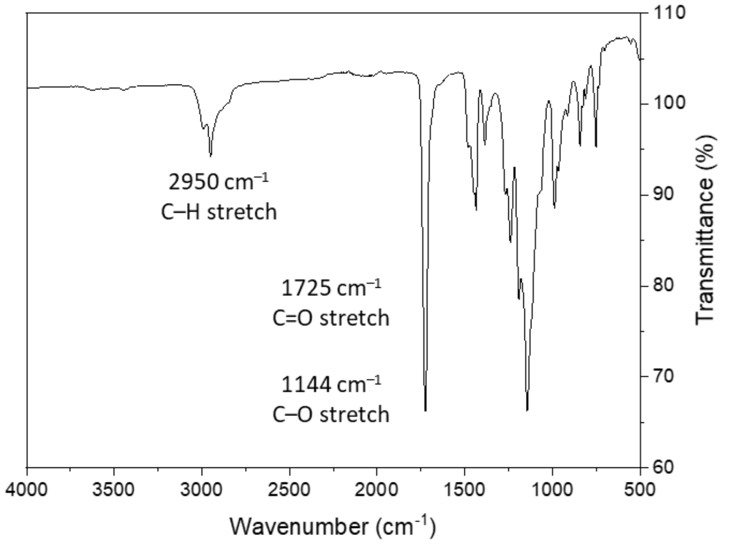

Figure S75. Labelled FT-IR spectrum of poly(**1**-*co*-MMA) (M_n 24,400 g mol^{-1} ($Đ_M$ 2.01), F_1/F_{MMA} = 16/84).

Representative size-exclusion chromatography analysis of poly(1-*co*-MMA)

Molecular Weight Averages

Peak	Mp (g/mol)	Mn (g/mol)	Mw (g/mol)	Mz (g/mol)	Mz+1 (g/mol)	Mv (g/mol)	PD
Peak 1	52503	39916	103253	258904	474439	231265	2.587

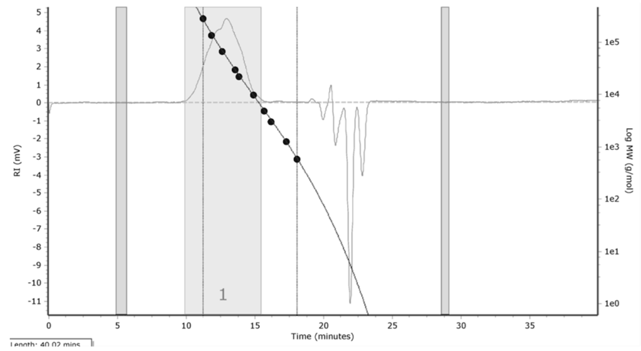

Figure S76. SEC trace of poly(**1**-co-MMA) (M_n 39,900 g mol^{-1} ($Đ_M$ 2.59), F_1/F_{MMA} = 2/98), in THF.

Molecular Weight Averages

Peak	Mp (g/mol)	Mn (g/mol)	Mw (g/mol)	Mz (g/mol)	Mz+1 (g/mol)	Mv (g/mol)	PD
Peak 1	21509	24422	49167	109468	201646	98054	2.013

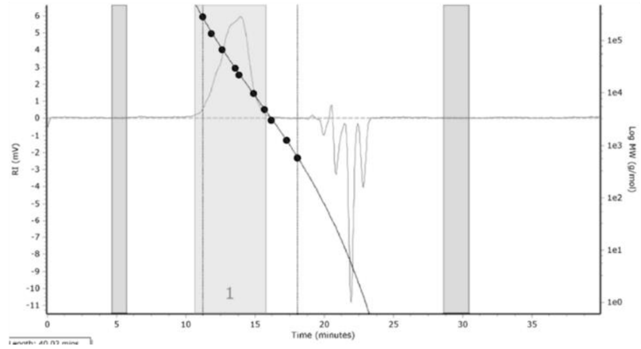

Figure S77. SEC trace of poly(1-co-MMA) (M_n 24,400 g mol^{-1} ($Đ_M$ 2.01), F_1/F_{MMA} = 16/84), in THF.

Molecular Weight Averages

Peak	Mp (g/mol)	Mn (g/mol)	Mw (g/mol)	Mz (g/mol)	Mz+1 (g/mol)	Mv (g/mol)	PD
Peak 1	17138	12554	31740	83242	165214	73099	2.528

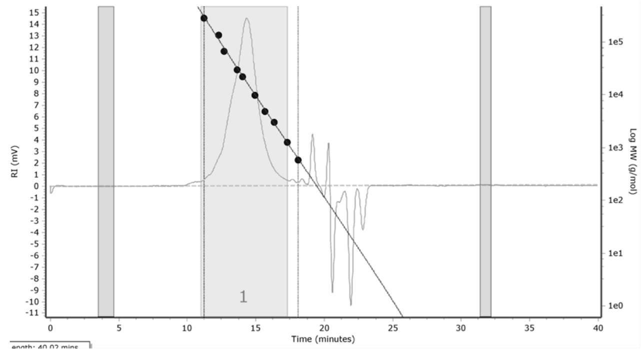

Figure S78. SEC trace of poly(1-co-MMA) (M_n 12,600 g mol^{-1} ($Đ_M$ 2.42), F_1/F_{MMA} = 33/87), in THF.

Thermal analysis of poly(1-*co*-MMA)

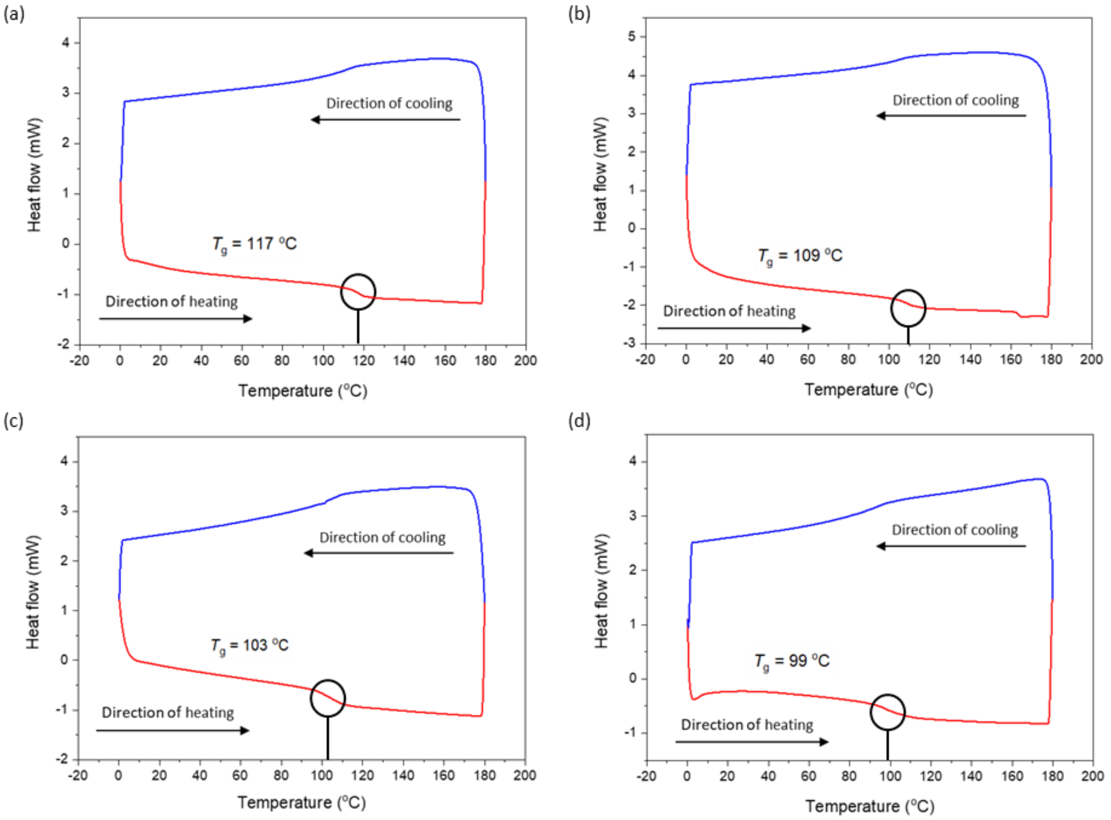

Figure S79. DSC traces (second heating and cooling cycle) of (a) poly(**1**-co-MMA) (M_n 39,900 g mol^{-1} ($Đ_M$ 2.59), F_1/F_{MMA} = 2/98), T_g = 117 °C. (b) poly(**1**-*co*-MMA) (M_n 24,400 g mol^{-1} ($Đ_M$ 2.01), F_1/F_{MMA} = 16/84), T_g = 109 °C. (c) poly(**1**-*co*-MMA) (M_n 13,800 g mol^{-1} ($Đ_M$ 2.42), F_1/F_{MMA} = 28/72), T_g = 99 °C. (d) poly(**1**-*co*-MMA) (M_n 12,600 g mol^{-1} ($Đ_M$ 2.53), F_1/F_{MMA} = 33/67), T_g = 99 °C. Exothermic and endothermic events are indicated by positive and negative heat flows, respectively.

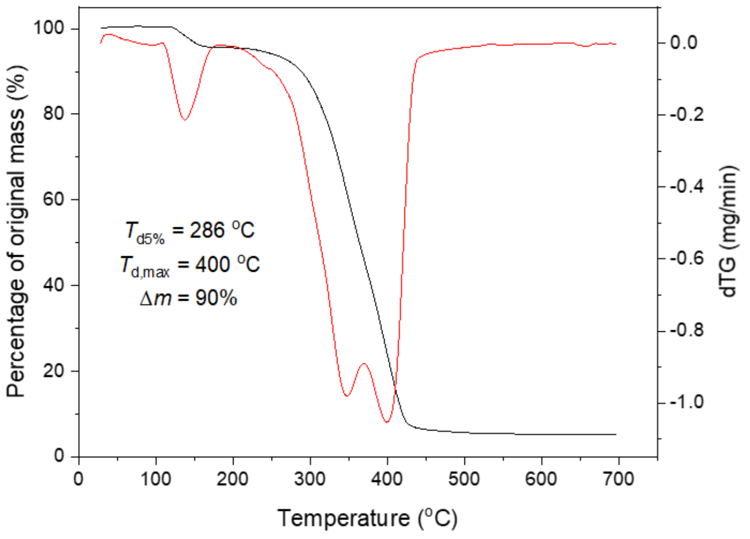

Figure S80. TGA trace of poly(**1**-*co*-MMA) (M_n 39,900 g mol^{-1} ($Đ_M$ 2.59), F_1/F_{MMA} = 2/98). The polymer was heated from 30 to 700 °C under argon at 10 °C min^{-1}. Obtained values: $T_{d5\%}$ = 286 °C, $T_{d,max}$ = 400 °C with 10 % char remaining at 700 °C.

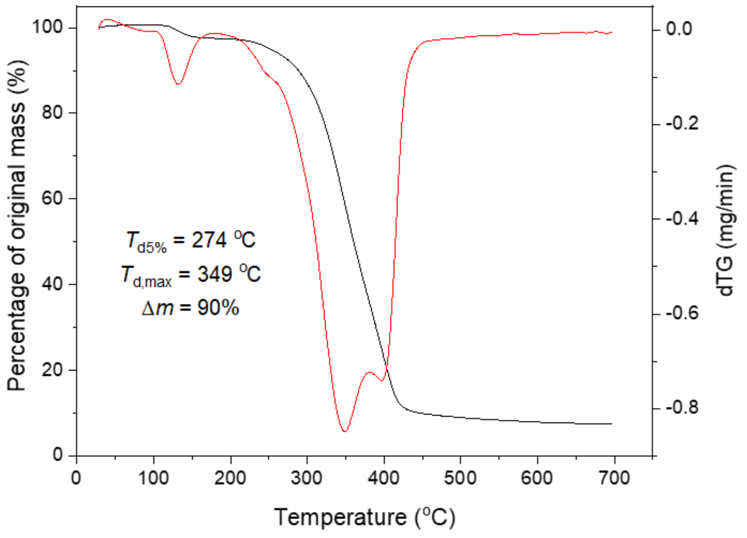

Figure S81. TGA trace of poly(**1**-*co*-MMA) (M_n 24,400 g mol^{-1} ($Đ_M$ 2.01), F_1/F_{MMA} = 16/84). The polymer was heated from 30 to 700 °C under argon at 10 °C min^{-1}. Obtained values: $T_{d5\%}$ = 274 °C, $T_{d,max}$ = 349 °C with 10 % char remaining at 700 °C.

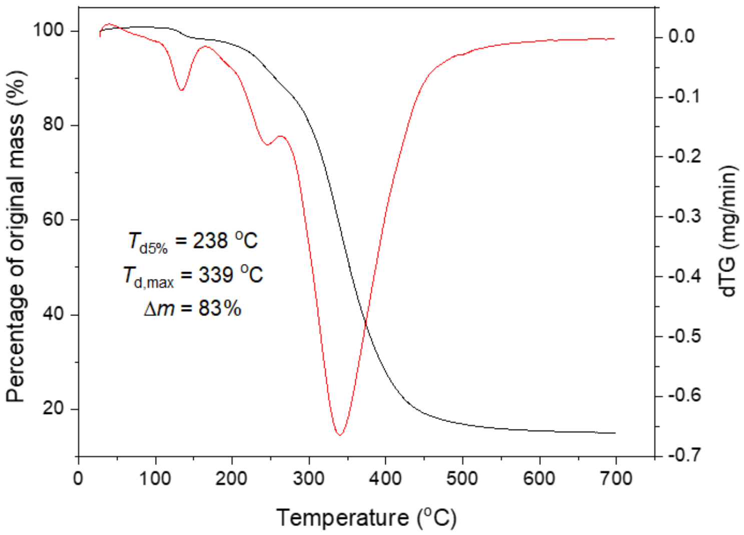

Figure S82. TGA trace of poly(**1**-*co*-MMA) (M_n 12,600 g mol^{-1} ($Đ_M$ 2.53), F_1/F_{MMA} = 33/67). The polymer was heated from 30 to 700 °C under argon at 10 °C min^{-1}. Obtained values: $T_{d5\%}$ = 238 °C, $T_{d,max}$ = 339 °C with 17 % char remaining at 700 °C.

NMR analysis of poly(1-*co*-MMA) supernatant

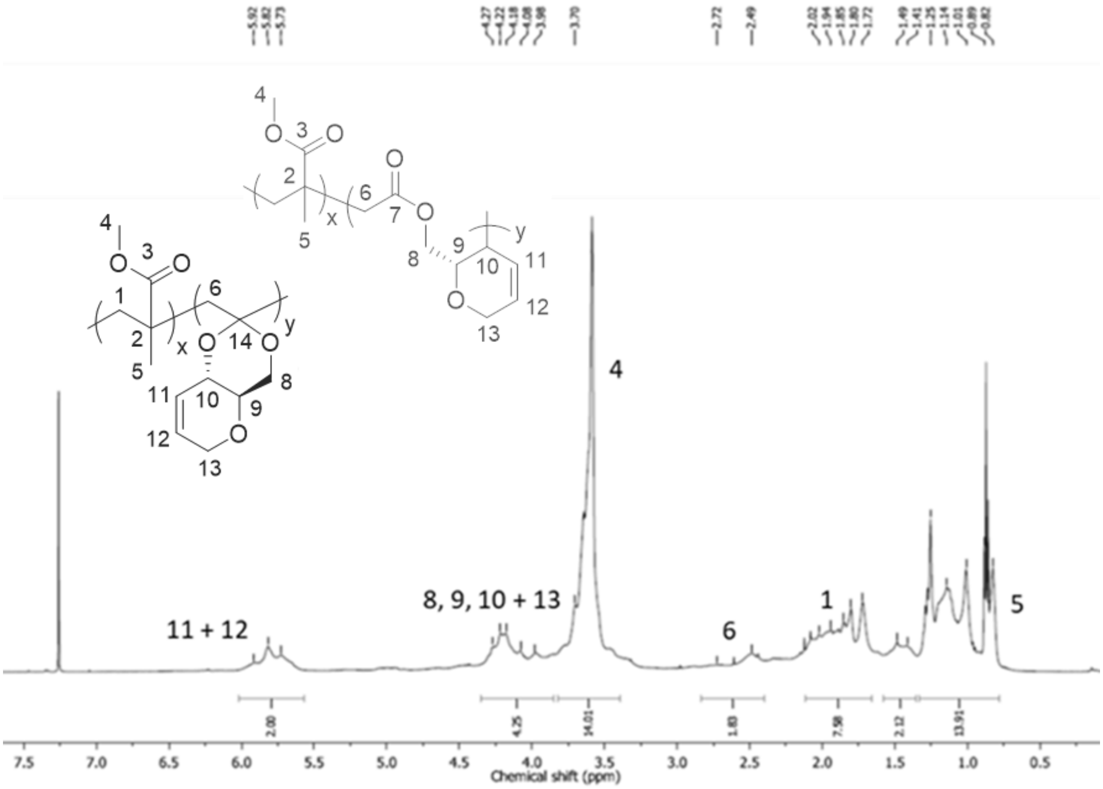

Figure S83. Annotated ^{1}H NMR spectrum ($CDCl_3$) of poly(**1**-*co*-MMA) supernatant (M_n 2,400 g mol^{-1} ($Đ_M$ 1.92), F_1/F_{MMA} = 21/79).

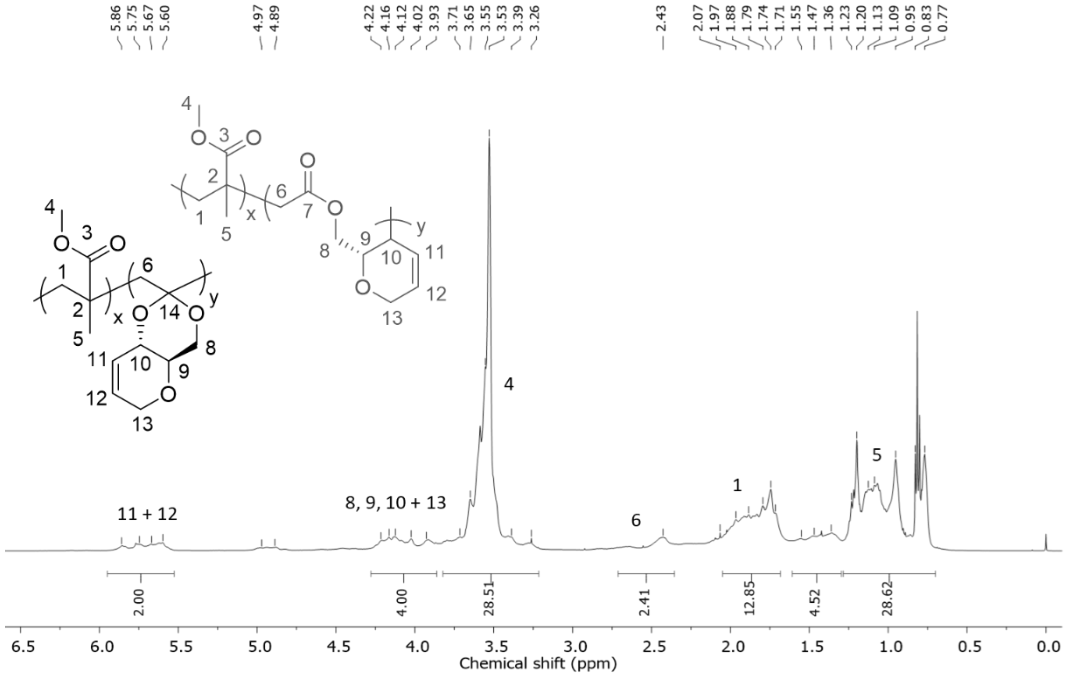

Figure S84. Annotated ^{1}H NMR spectrum ($CDCl_3$) of poly(**1**-*co*-MMA) supernatant (M_n 3,500 g mol^{-1} ($Đ_M$ 1.72), F_1/F_{MMA} = 14/86).

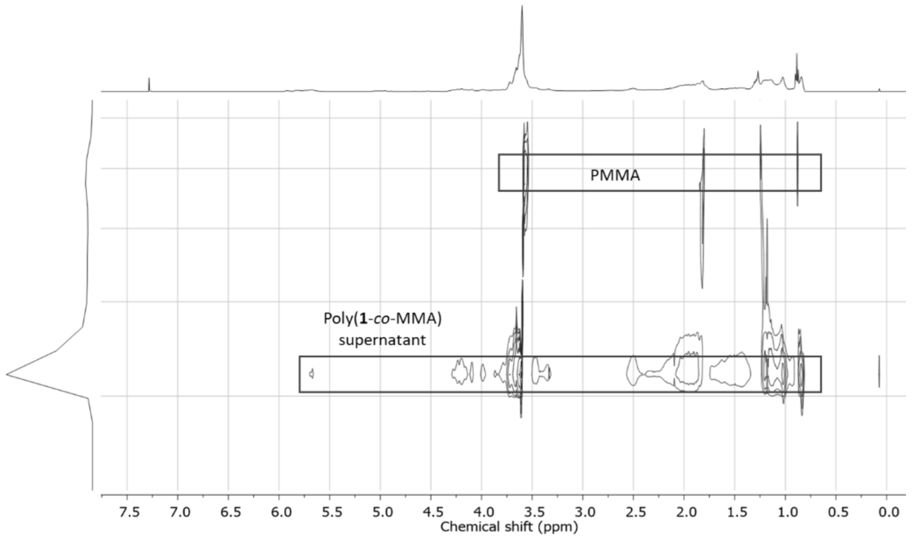

Figure S85. ^{1}H DOSY NMR spectrum ($CDCl_3$) of poly(**1**-*co*-MMA) supernatant (M_n 3,500 g mol^{-1} ($Đ_M$ 1.72), F_1/F_{MMA} = 14/86)

Representative size-exclusion chromatography analysis of poly(1-*co*-MMA) supernatant

Molecular Weight Averages

Peak	Mp (g/mol)	Mn (g/mol)	Mw (g/mol)	Mz (g/mol)	Mz+1 (g/mol)	Mv (g/mol)	PD
Peak 1	10950	5825	9248	12867	16023	12378	1.588

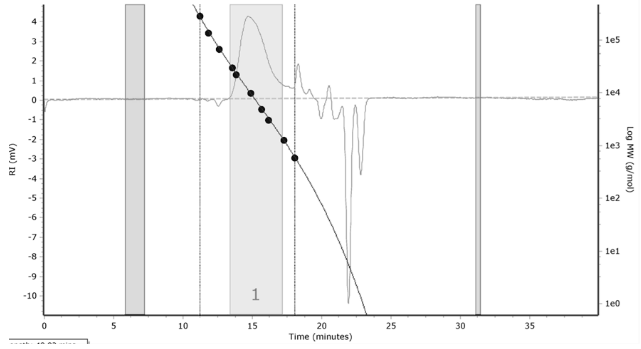

Figure S86. SEC trace of poly(**1**-*co*-MMA) supernatant (M_n 5,800 g mol^{-1} ($Đ_M$ 1.59), F_1/F_{MMA} = 7/93), in THF.

Molecular Weight Averages

Peak	Mp (g/mol)	Mn (g/mol)	Mw (g/mol)	Mz (g/mol)	Mz+1 (g/mol)	Mv (g/mol)	PD
Peak 1	6454	3466	5949	8553	10735	8210	1.716

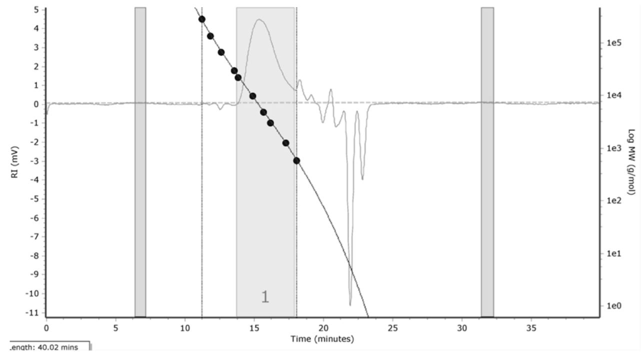

Figure S87. SEC trace of poly(**1**-*co*-MMA) supernatant (M_n 3,500 g mol^{-1} ($Đ_M$ 1.72), F_1/F_{MMA} = 14/86), in THF.

Molecular Weight Averages

Peak	Mp (g/mol)	Mn (g/mol)	Mw (g/mol)	Mz (g/mol)	Mz+1 (g/mol)	Mv (g/mol)	PD
Peak 1	4304	2414	4644	7188	9438	6842	1.924

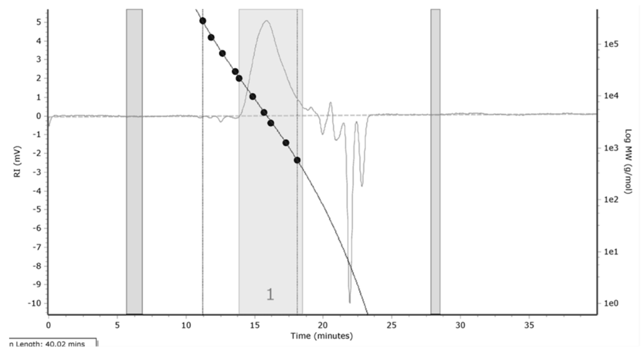

Figure S88. SEC trace of poly(**1**-*co*-MMA) supernatant (M_n 2,400 g mol^{-1} ($Đ_M$ 1.92), F_1/F_{MMA} = 21/79), in THF.

Thermal analysis of poly(1-*co*-MMA) supernatant

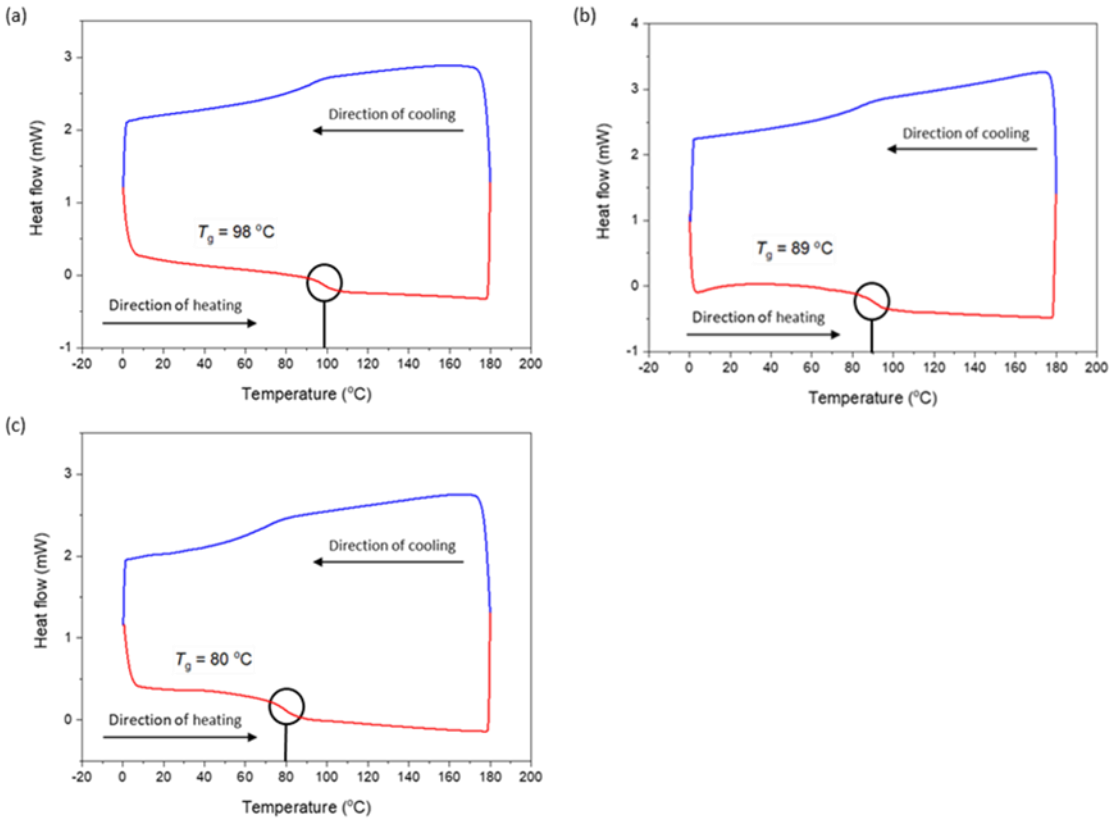

Figure S89. DSC traces (second heating and cooling cycle) of (a) poly(**1**-*co*-MMA) supernatant (M_n 5,800 g mol^{-1} ($Đ_M$ 1.59), F_1/F_{MMA} =7/93), T_g = 98 °C. (b) poly(**1**-*co*-MMA) supernatant (M_n 3,500 g mol^{-1} ($Đ_M$ 1.72), F_1/F_{MMA} = 14/86), T_g = 89 °C. (c) poly(**1**-*co*-MMA) supernatant (M_n 2,400 g mol^{-1} ($Đ_M$ 1.92), F_1/F_{MMA} =21/79), T_g = 80 °C. Exothermic and endothermic events are indicated by positive and negative heat flows, respectively.

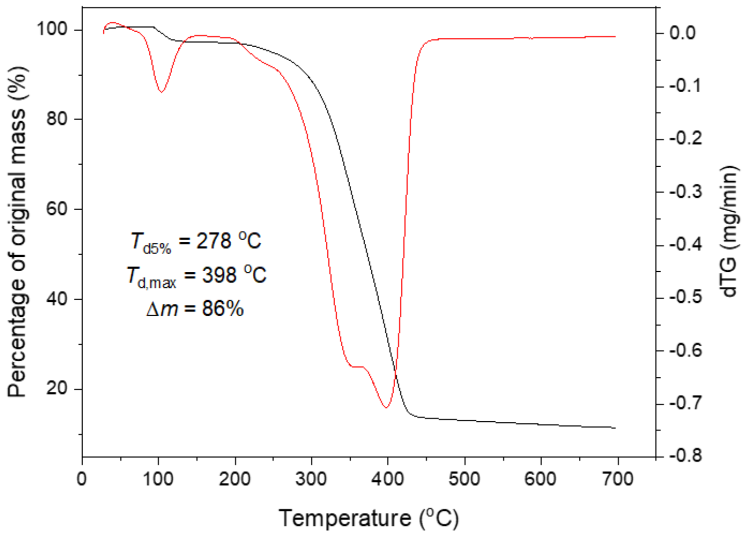

Figure S90. TGA trace of poly(**1**-*co*-MMA) supernatant (M_n 5,800 g mol^{-1} ($Đ_M$ 1.59), F_1/F_{MMA} = 7/97). The polymer was heated from 30 to 700 °C under argon at 10 °C min^{-1}. Obtained values: $T_{d5\%}$ = 278 °C, $T_{d,max}$ = 398 °C with 14 % char remaining at 700 °C.

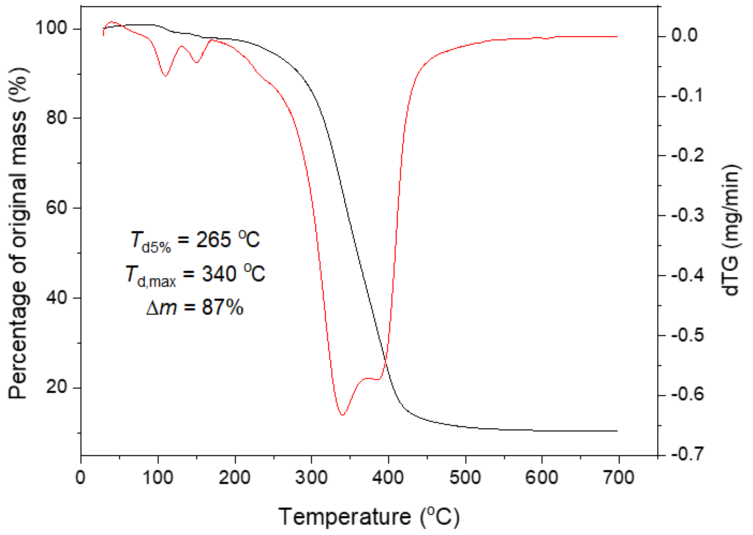

Figure S91. TGA trace of poly(**1**-*co*-MMA) supernatant (M_n 3,500 g mol^{-1} ($Đ_M$ 1.72), F_1/F_{MMA} = 14/86). The polymer was heated from 30 to 700 °C under argon at 10 °C min^{-1}. Obtained values: $T_{d5\%}$ = 265 °C and $T_{d,max}$ = 340 °C with 13 % char remaining at 700 °C.

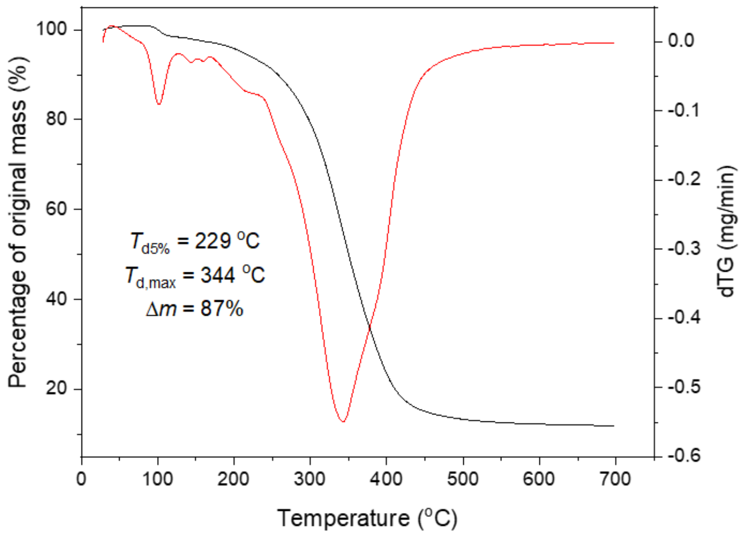

Figure S92. TGA trace of poly(**1**-*co*-MMA) supernatant (M_n 2,400 g mol^{-1} ($Đ_M$ 1.92), F_1/F_{MMA} = 21/79). The polymer was heated from 30 to 700 °C under argon at 10 °C min^{-1}. Obtained values: $T_{d5\%}$ = 229 °C, $T_{d,max}$ = 344 °C with 13 % char remaining at 700 °C.

Thermal analysis of PMMA

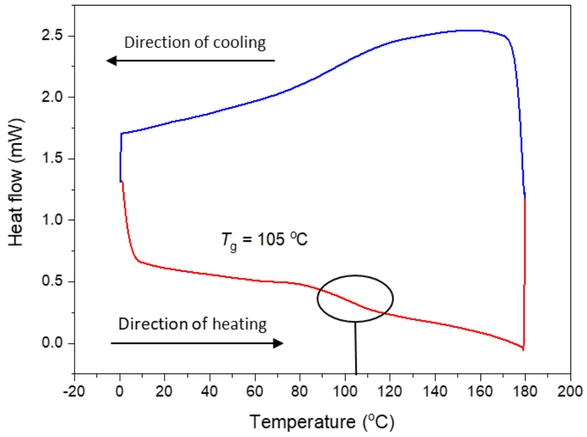

Figure S93. DSC trace of PMMA (M_n 75,700 g mol^{-1} ($Đ_M$ 1.82)), second heating and cooling cycle between 0 and 180 °C, T_g = 105 °C. Exothermic and endothermic events are indicated by positive and negative heat flows, respectively.

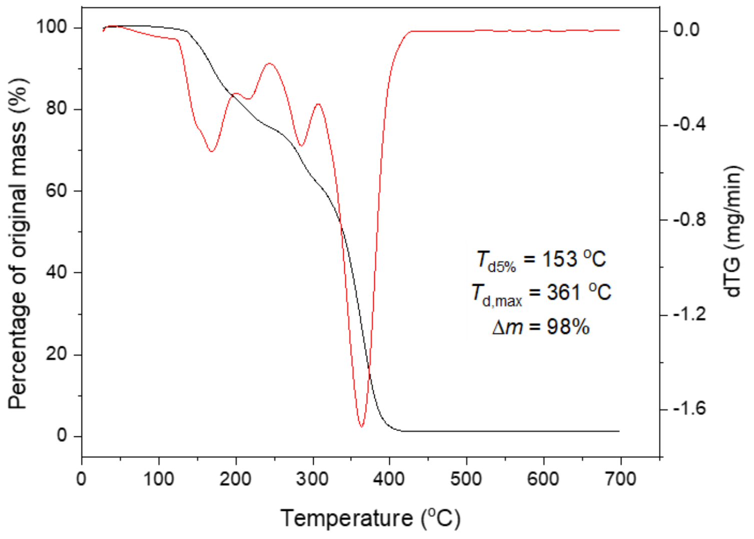

Figure S94. TGA trace of PMMA (M_n 75,700 g mol^{-1} ($Đ_M$ 1.82)). The polymer was heated from 30 to 700 °C under argon at 10 °C min^{-1}. Obtained values: $T_{d5\%}$ = 153 °C, $T_{d,max}$ = 361 °C with 2 % char at 700 °C.

8. Polymer degradation

Hydrolytic degradation of poly(1)

A solution of base (0.5 mL, 1 mol L^{-1} in H_2O/MeOH) was mixed with a solution of poly(**1**) (0.010 g) in THF (1 mL) and stirred at room temperature for 4 h. Once complete, the solvent was removed under reduced pressure. The crude solid was dissolved in $CHCl_3$ and filtered to remove residual base and concentrated in *vacuo. The degraded products were then subjected to* *^{1}H NMR spectroscopy and SEC analysis*.

Table S4. Hydrolytic degradation of poly(**1**).

Entry	F_E[a]	Base	Solvent	$M_{n,start}$[b] (g mol^{-1})	$Đ_{M,start}$[b]	$M_{n,end}$[b] (kg mol^{-1})	$Đ_{M,end}$[b]	%M_n change[c]
1	0.65	NaOH	H_2O	4,700	2.11	360	1.25	92
2	0.65	NaOH	MeOH	4,700	2.11	350	1.29	93
3	0.65	KOH	MeOH	4,700	2.11	300	1.30	94

[a]Fraction of ester linkages in poly(**1**); [b]Number-average molar mass and Dispersity ($M_{n,SEC}$, $Đ_M$), calculated by SEC relative to polystyrene standards in THF eluent; [c] Percentage variation compared to the polymer original $M_{n,SEC}$.

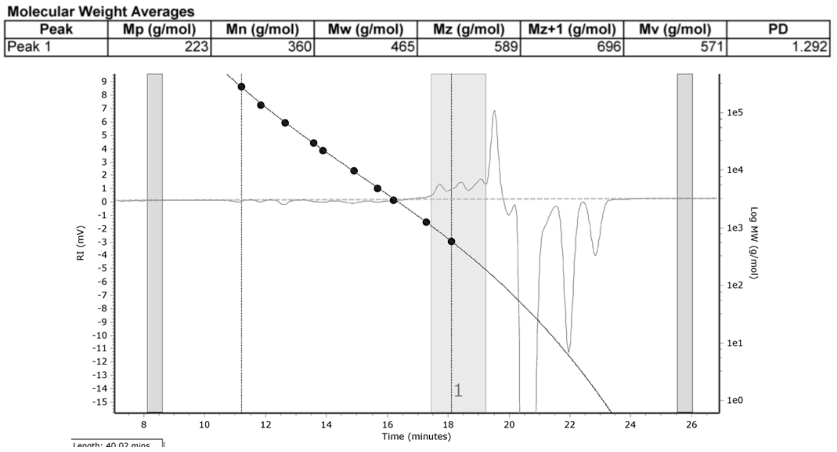

Figure S95. SEC trace of degraded products obtained from poly(**1**) (M_n 360 g mol^{-1} ($Đ_M$ 1.29)), in THF.

Hydrolytic degradation of poly(1-*co*-MMA)

A solution of base (0.5 mL, 1 mol L^{-1} in H_2O/MeOH) was mixed with a solution of poly(**1**-*co*-MMA) (0.010 g) in THF (1 mL) and stirred at room temperature for 4 h. Once complete, the solvent was removed under reduced pressure. The crude solid was dissolved in $CHCl_3$ and filtered to remove residual base and concentrated in *vacuo. The degraded products were then subjected to* *^{1}H NMR spectroscopy and SEC analysis*.

Table S5. Hydrolytic degradation of poly(**1**-*co*-MMA).

Entry	F_1[a]	Base	Solvent	$M_{n,start}$[b] (g mol^{-1})	$Đ_{M,start}$[b]	$M_{n,end}$[b] (kg mol^{-1})	$Đ_{M,end}$[b]	%M_n change[c]
1	0	NaOH	H_2O	75,700	1.82	77100	1.84	0
2	0	NaOH	MeOH	75,700	1.82	76700	1.83	0
3	0.16	NaOH	H_2O	24,400	2.01	19900	1.72	18
4	0.16	NaOH	MeOH	24,400	2.01	16200	2.72	34
5	0.16	KOH	MeOH	24,400	2.01	12100	1.76	50
6	0.33	NaOH	H_2O	12,600	2.43	6300	2.31	50
7	0.33	NaOH	MeOH	12,600	2.43	3000	1.67	78
8	0.33	KOH	MeOH	12,600	2.43	1300	1.88	90

[a]Fraction of **1** incorporated into poly(**1**-*co*-MMA); [b]Number-average molar mass and Dispersity ($M_{n,SEC}$, $Đ_M$), calculated by SEC relative to polystyrene standards in THF eluent; [c] Percentage variation compared to the co-polymer original $M_{n,SEC}$.

Table S6. Time monitored hydrolytic degradation of poly(**1**-*co*-MMA).

Entry	F_1[a]	Base	Solvent	Time (h)	$M_{n,start}$[b] (g mol^{-1})	$Đ_{M,start}$[b]	$M_{n,end}$[b] (kg mol^{-1})	$Đ_{M,end}$[b]	$\%M_n$[c]
1	0.33	NaOH	H_2O	0.5	12600	2.43	10500	3.05	83
2	0.33	NaOH	H_2O	1	12600	2.43	8900	2.50	70
3	0.33	NaOH	H_2O	4	12600	2.43	6300	2.31	50

[a]Fraction of **1** incorporated into poly(**1**-*co*-MMA); [b]Number-average molar mass and Dispersity ($M_{n,SEC}$, $Đ_M$), calculated by SEC relative to polystyrene standards in THF eluent; [c]Percentage variation compared to the co-polymer original $M_{n,SEC}$.

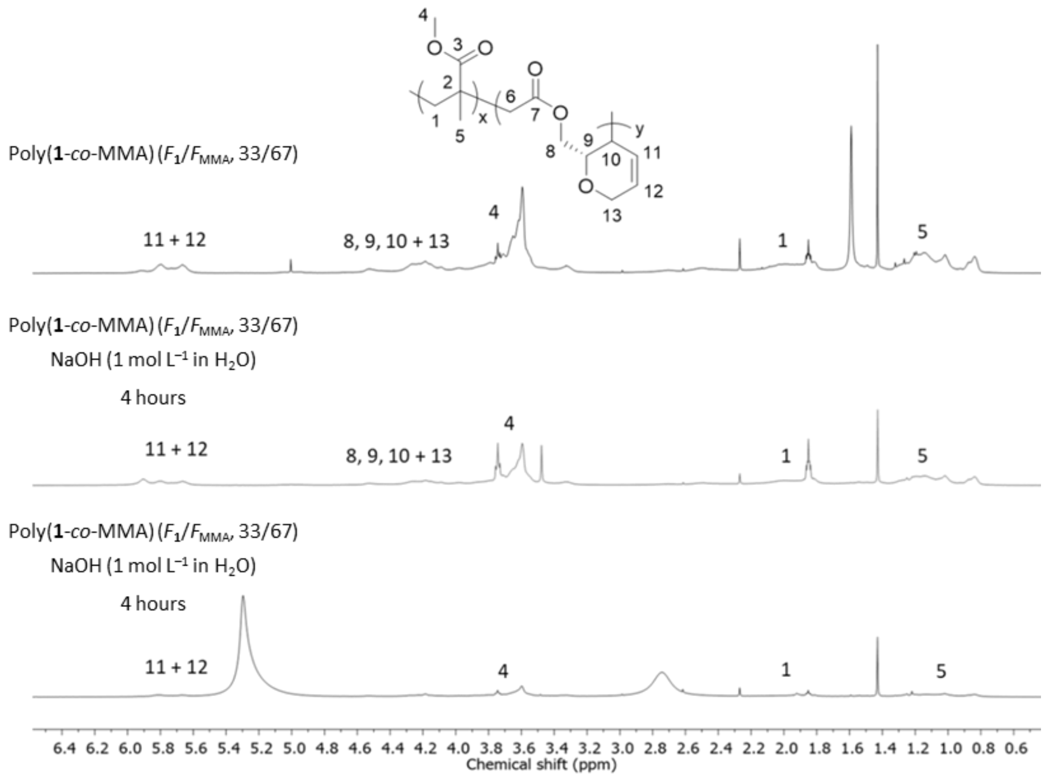

Figure S96. Stacked ^{1}H NMR spectra following the degradation of poly(**1**-*co*-MMA) (F_1/F_{MMA} = 33/67) over 4 hours. Carried out at rt in THF, in the presence of NaOH (1 mol L^{-1} in H_2O).

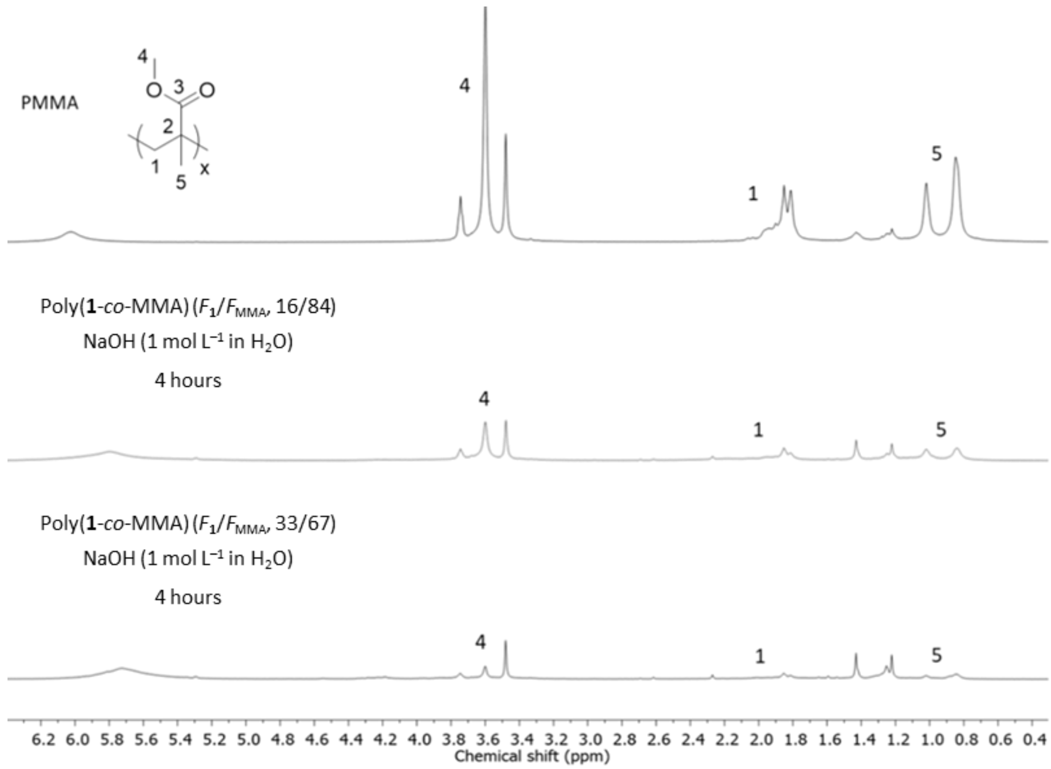

Figure S97. Stacked ^{1}H NMR spectra showing the degradation of poly(**1**-*co*-MMA), with varied F_1/F_{MMA} incorporation, after 4 hours *versus* PMMA. Carried out at rt in THF, in the presence of NaOH (1 mol L^{-1} in H_2O).

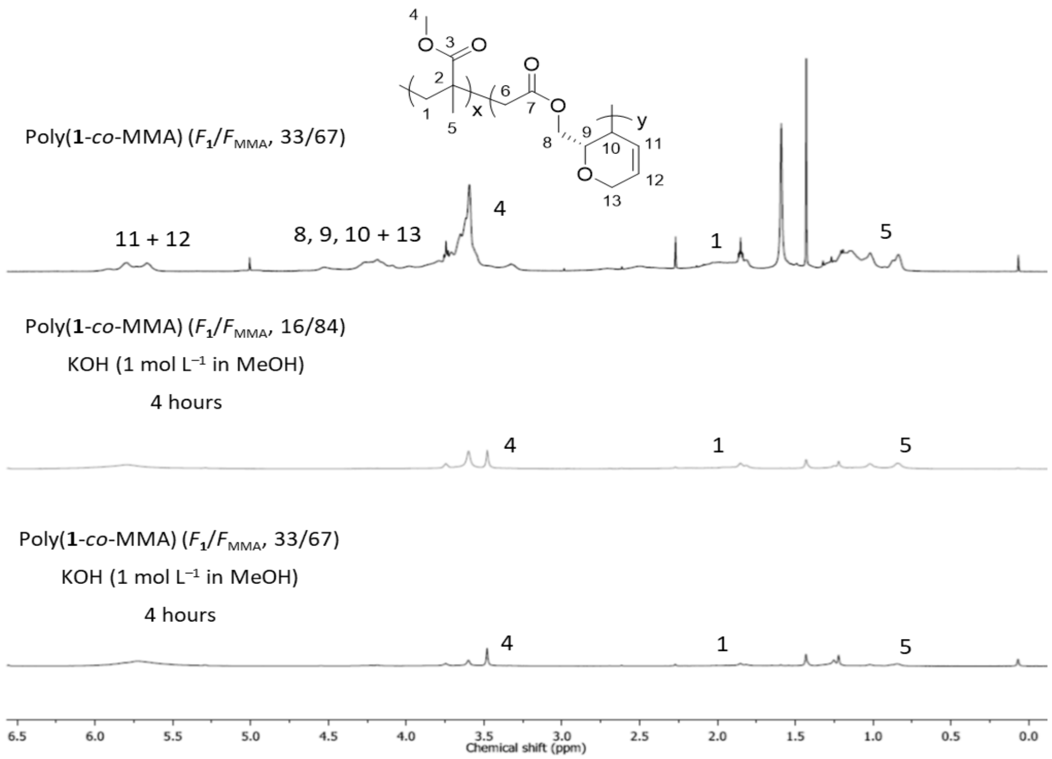

Figure S98. Stacked ^{1}H NMR spectra showing the degradation of poly(**1**-*co*-MMA), with varied F_1/F_{MMA} incorporation, after 4 hours. Carried out at rt in THF, in the presence of KOH (1 mol L^{-1} in MeOH).

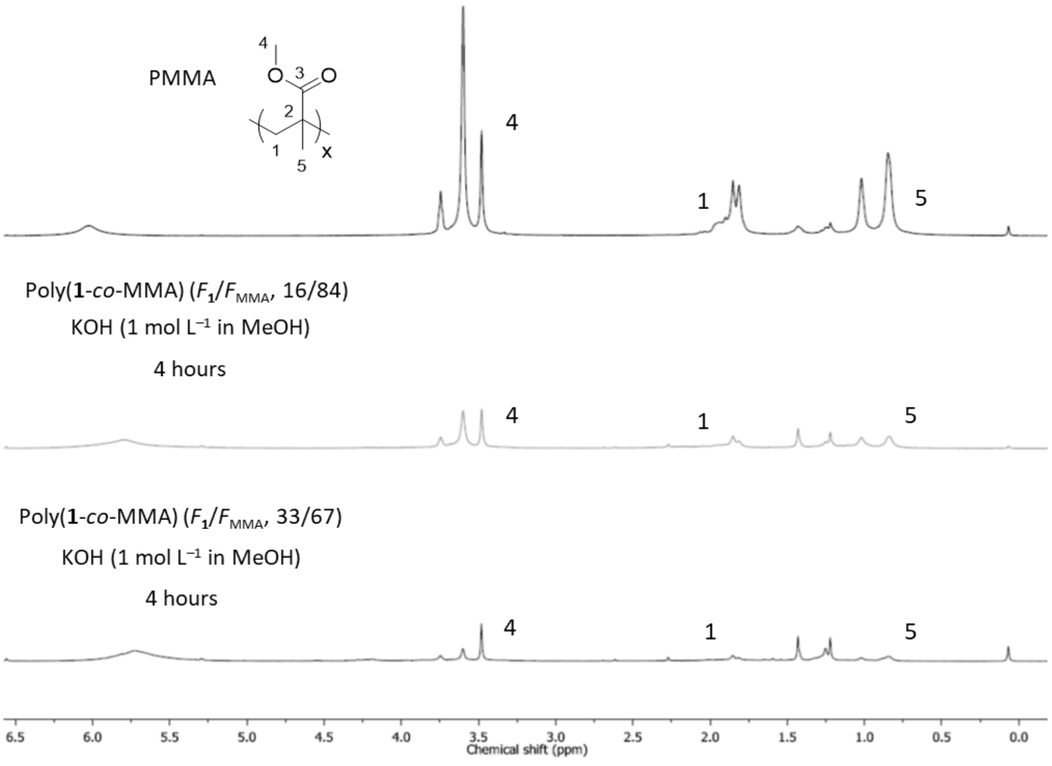

Figure S99. Stacked ^{1}H NMR spectra showing the degradation of poly(**1**-*co*-MMA), with varied F_1/F_{MMA} incorporation, after 4 hours *versus* PMMA. Carried out at rt in THF, in the presence of KOH (1 mol L^{-1} in MeOH).

Complementary data

Mechanistic considerations

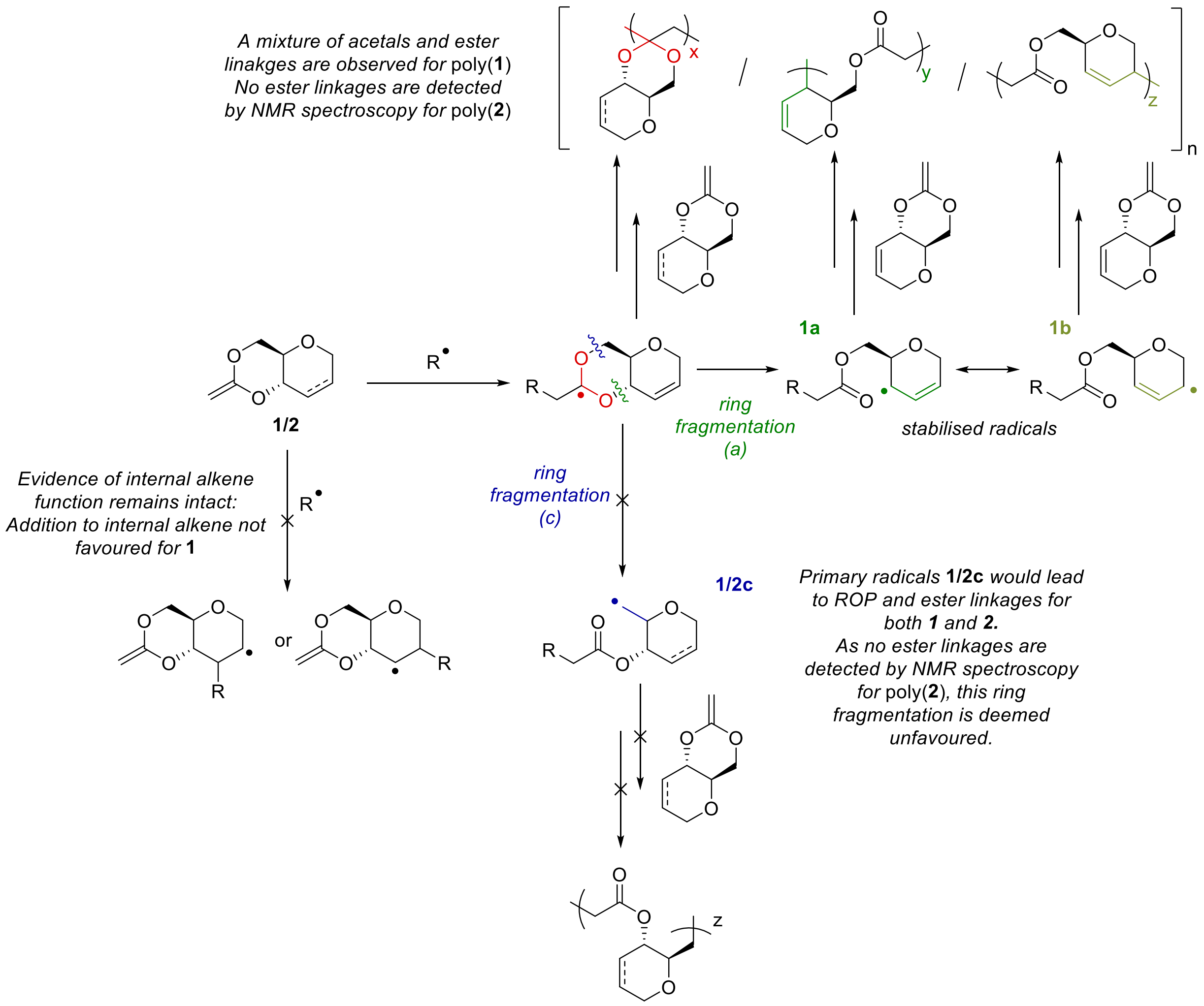

Scheme S3. Proposed mechanism for the free radical polymerization of **1** and **2.**

Selectivity towards ROP of common CKA monomers

Table S7. Ring-opening behavior of some common CKA monomers.

CKA Monomer	[*Ref.*]	Selectivity towards rROP and ester linkages (%)
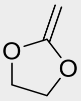	[3][4]	50–100
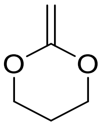	[3][5]	36–85
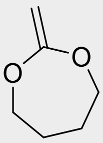 (MDO)	[6][7][8]	100
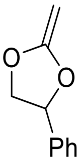	[7][9][10]	100
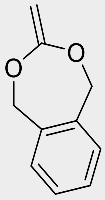 (BMDO)	[5][11]	100
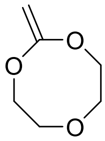	[12]	100
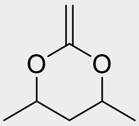	[13]	91
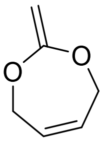	[5][8]	20–80

References

1. Hardy, C.; Kociok-Köhn, G.; Buchard, A., UV degradation of poly(lactic acid) materials through copolymerisation with a sugar-derived cyclic xanthate. *Chem. Commun.* **2022,** *58* (36), 5463-5466.

2. Hardy, C.; Kociok-Köhn, G.; Buchard, A., Variations around the presence and position of sulfur in sugar-derived cyclic monomers: influence on polymerisation thermodynamics, polymer sequence and thermal properties. *Polym. Chem.* **2023,** *14* (5), 623-632.

3. Bailey, W. J.; Ni, Z.; Wu, S.-R., Synthesis of poly-ϵ-caprolactone via a free radical mechanism. Free radical ring-opening polymerization of 2-methylene-1,3-dioxepane. *J. Polym. Sci.* **1982,** *20* (11), 3021-3030.

4. Bailey, W. J.; Chen, P. Y.; Chen, S.-C.; Chiao, W.-B.; Endo, T.; Gapud, B.; Kuruganti, V.; Lin, Y.-N.; Ni, Z.; Pan, C.-Y.; Shaffer, S. E.; Sidney, L.; Wu, S.-R.; Yamamoto, N.; Yamazaki, N.; Yonezawa, K.; Zhou, L.-L., Free radical ring-opening polymerization and its use to make biodegradable polymers and functionally terminated oligomers. *Makromol. Chem. Macromol. Symp.* **1986,** *6* (1), 81-100.

5. Tardy, A.; Nicolas, J.; Gigmes, D.; Lefay, C.; Guillaneuf, Y., Radical ring-opening polymerization: scope, limitations, and application to (bio)degradable materials. *Chem. Rev.* **2017,** *117* (3), 1319-1406.

6. Jin, S. R.; Gonsalves, K. E., A study of the mechanism of the free-radical ring-opening polymerization of 2-methylene-1,3-dioxepane. *Macromolecules* **1997,** *30* (10), 3104-3106.

7. Endo, T.; Yako, N.; Azuma, K.; Nate, K., Ring-opening polymerization of 2-methylene-4-phenyl-1,3-dioxolane. *Die Makromol. Chem.* **1985,** *186* (8), 1543-1548.

8. Plikk, P.; Tyson, T.; Finne-Wistrand, A.; Albertsson, A. C., Mapping the characteristics of the radical ring-opening polymerization of a cyclic ketene acetal towards the creation of a functionalized polyester. *J. Polym. Sci. Part A* **2009,** *47* (18), 4587-4601.

9. Cho, I.; Gong, M. S., Exploratory ring-opening polymerization. V. Radical ring-opening and cationic polymerization of 2-methylene-4-phenyl-1, 3-dioxolane. *J. Polym. Sci. Polym. Lett. Ed.* **1982,** *20* (7), 361-364.

10. Bailey, W. J.; Wu, S. R.; Ni, Z., Synthesis and free radical ring-opening polymerization of 2-methylene-4-phenyl-1, 3-dioxolane. *Makromol. Chem.* **1982,** *183* (8), 1913-1920.

11. Bailey, W. J.; Ni, Z.; Wu, S. R., Free radical ring-opening polymerization of 4, 7-dimethyl-2-methylene-1, 3-dioxepane and 5, 6-benzo-2-methylene-1, 3-dioxepane. *Macromolecules* **1982,** *15* (3), 711-714.

12. Hiracuri, Y.; Tokiwa, Y., Synthesis of copolymers composed of 2-methylene-1,3,6-trioxocane and vinyl monomers and their enzymatic degradation. *J. Polym. Sci. Part A* **1993,** *31* (12), 3159-3163.

13. Zhende, N.; Bailey, W. J., Synthesis and free radical polymerization of 2-methylene-1,3-dioxane derivatives. . *Acta Polym.* **1987,** *1* (5), 379-383.
